# Regulation of the Hepatitis B virus replication and gene expression by the multi-functional protein TARDBP

**DOI:** 10.1038/s41598-019-44934-5

**Published:** 2019-06-11

**Authors:** Grace Naswa Makokha, Hiromi Abe-Chayama, Sajeda Chowdhury, C. Nelson Hayes, Masataka Tsuge, Tadahiko Yoshima, Yuji Ishida, Yizhou Zhang, Takuro Uchida, Chise Tateno, Rie Akiyama, Kazuaki Chayama

**Affiliations:** 1 Department of Gastroenterology and Metabolism, Institute of Biomedical and Health Science, Hiroshima, Japan; 2 Liver Research Project Center, Hiroshima, Japan; 3 Natural Science Center for Basic Research and Development, Hiroshima, Japan; 4 Center for Medical Specialist Graduate Education and Research, Hiroshima, Japan; 5 Laboratory for Digestive Diseases, RIKEN Center for Integrative Medical Sciences Hiroshima University, 1-2-3 Kasumi, Minami-ku, Hiroshima-shi, 734-8551 Japan; 6 PhoenixBio Co., Ltd., 3-4-1 Kagamiyama, Higashihiroshima, 739-0046 Japan

**Keywords:** Hepatitis B virus, Virus-host interactions

## Abstract

Hepatitis B virus (HBV) infects the liver and is a key risk factor for hepatocellular carcinoma. Identification of host factors that support viral replication is important to understand mechanisms of viral replication and to develop new therapeutic strategies. We identified TARDBP as a host factor that regulates HBV. Silencing or knocking out the protein in HBV infected cells severely impaired the production of viral replicative intermediates, mRNAs, proteins, and virions, whereas ectopic expression of TARDBP rescued production of these products. Mechanistically, we found that the protein binds to the HBV core promoter, as shown by chromatin precipitation as well as mutagenesis and protein-DNA interaction assays. Using LC-MS/MS analysis, we also found that TARDBP binds to a number of other proteins known to support the HBV life cycle, including NPM1, PARP1, Hsp90, HNRNPC, SFPQ, PTBP1, HNRNPK, and PUF60. Interestingly, given its key role as a regulator of RNA splicing, we found that TARDBP has an inhibitory role on pregenomic RNA splicing, which might help the virus to export its non-canonical RNAs from the nucleus without being subjected to unwanted splicing, even though mRNA nuclear export is normally closely tied to RNA splicing. Taken together, our results demonstrate that TARDBP is involved in multiple steps of HBV replication via binding to both HBV DNA and RNA. The protein’s broad interactome suggests that TARDBP may function as part of a RNA-binding scaffold involved in HBV replication and that the interaction between these proteins might be a target for development of anti-HBV drugs.

## Introduction

Chronic hepatitis B virus (HBV) infection is a major risk factor for liver cirrhosis and hepatocellular carcinoma (HCC). Worldwide, it is estimated that more than 200 million people are chronic carriers of HBV^1^, with infection resulting in about one million deaths per year^2^. Control of HBV replication is a key strategy to reduce the morbidity and mortality associated with chronic HBV infection^3^. However, the molecular mechanisms underlying viral replication remain elusive. Currently, interferon and nucleo(t)side analog reverse transcriptase inhibitors (NAs) are used to suppress HBV replication, but problems with adverse effects and the risk of drug resistance remain^4^. Therefore, identifying cellular host factors that are required to support the HBV life cycle will be a major step forward in the process of developing new anti-HBV drugs.

The hepatitis B viral genome is present in infectious particles in the form of 3.2 kb partially double -stranded, relaxed circular DNA (rcDNA). Following infection, the virus gains entry into hepatocytes via its receptor, the human sodium taurocholate cotransporting polypeptide (NTCP)^5^. The viral genome is uncoated in the cytoplasm, then transported to the nucleus where the rcDNA is converted to covalently closed circular DNA (cccDNA). The cccDNA serves as the template for synthesis of genomic and subgenomic viral transcripts^6^. Replication of the virus occurs by reverse transcription of pregenomic RNA^7^. Four viral promoters, Core, Pre S1, Pre S2, and X, and two enhancers, enhancer I and enhancer II, control the transcription of HBV^8^. In addition, a vast number of host cellular transcription factors, including nuclear receptors, regulate HBV transcription by acting on the HBV promoters and enhancers^9^.

To further understand the molecular mechanisms behind the control of HBV replication and pathogenesis, we identified novel host factors that could play a role in the viral lifecycle using primary human hepatocytes derived from human hepatocyte transplanted chimeric mice, an infection system that we recently established^10^. One of the candidates we identified was a gene that codes for the trans-active response DNA binding protein (TARDBP). The protein was initially identified by Ignatius *et al*. as a factor that binds to TAR DNA of HIV^11^. TARDBP belongs to the family of heterogeneous nuclear ribonucleoproteins (hnRNPs) that serve multiple roles in the generation and processing of RNA, including transcription, splicing, transport, and mRNA stability^12,13^. TARDBP contains two RNA recognition motifs (RRMs), through which it binds to UG/TG repeats in RNA/DNA, respectively, and a C-terminal glycine-rich domain that is considered important for protein-protein interactions^14^. In disease, TARDBP is known to be frequently mutated in sporadic and familial amyotrophic lateral sclerosis (ALS), as well as in patients with frontotemporal lobar degeneration (FTLD), providing evidence of a direct link between TARDBP abnormalities and neurodegeneration^15^. The role of this protein in HBV pathogenesis remains unreported.

In this study, we demonstrate that TARDBP is a novel host factor that can enhance HBV gene expression via both transcriptional and post-transcriptional mechanisms. We show that silencing of the protein results in inhibition of HBV gene expression. We further provide evidence that TARDBP activates transcription from the core promoter of HBV and that TARDBP forms complexes with other proteins known to support the lifecycle of HBV. Moreover, we report that the protein has an inhibitory effect on splicing of pregenomic (pg) HBV RNA, which may help the virus export noncanonical unspliced RNAs from the nucleus despite the normally close association between splicing and nuclear export.

## Results

### Silencing of TARDBP inhibits HBV infection and gene expression in cell cultures

**Figure 1:**
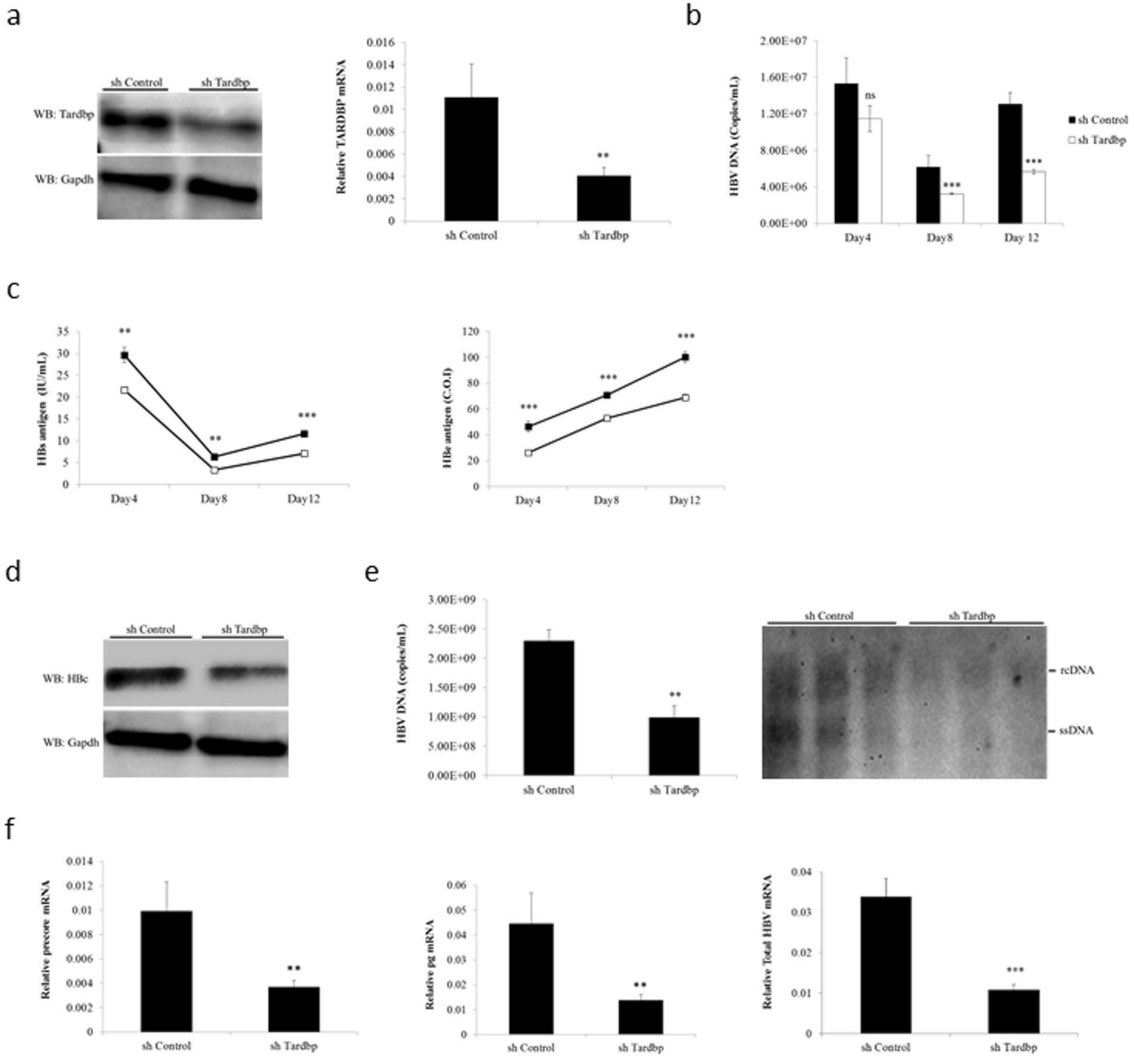
Endogenous TARDBP regulates HBV gene expression in a HBV infection model. (**a**) NTCP-HepG2 C4 cells were transduced with a lentiviral vector allowing the expression of a control shRNA (shControl) or shRNA directed against TARDBP (shTardbp). After selection with puromycin, reduction of TARDBP mRNA and protein level by shRNA was confirmed by qPCR and western blotting, respectively. (**b**) The cells in (**a**) were then infected with HBV in duplicate sets for 12 days. Supernatants were collected at the indicated time points for extracellular HBV DNA analysis. (**c**) A separate portion of supernatants from (**b**) were also analyzed by CLEIA for measurement of HBs and HBe antigen levels. (**d**) To detect the level of the HBV core protein, one set of the cells were harvested at the end of the 12-day infection period for protein lysis and subjected to western blotting using the anti-HBc antibody. (**e**) For detection of the core-associated HBV DNA, the lysates in (**d**) were subjected to immunoprecipitation by an anti-HBc antibody followed by qPCR (left panel) and Southern blotting (right panel). (**f**) Total RNA was extracted from the other set of cells from after the 12-day infection period in (**b**) and subjected to RT qPCR to detect HBV precore, pregenomic (pg) and total mRNAs. Gene expression was normalized to that of GAPDH. The data is shown as the mean ± SD (n = 3 per bar), **P < 0.01, ***P < 0.001 and ns, non-significant.

**Figure 2:**
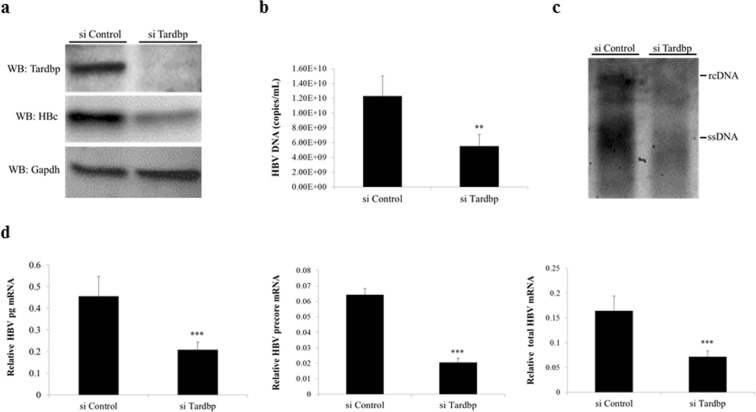
Silencing of TARDBP represses HBV transcription in a HBV-producing cell line. (**a**) The T23 cell line, which stably expresses HBV plasmid, was transfected with the negative control siRNA (siControl) or the TARDBP siRNA (siTARDBP) in duplicate sets for 7 days. One set of the cells was lysed, and the TARDBP protein and core protein were quantified by western blotting using specific antibodies. (**b**) The supernatants were harvested from one set of the cells in (**a**) and analyzed for extracellular HBV DNA. (**c**) For the intracellular HBV DNA, protein lysates harvested in A were immunoprecipated with an anti-HBc antibody, followed by Southern blotting. (**d**) The total RNA was extracted from the second set of cells and subjected to RT qPCR to detect the HBV mRNAs (pregenomic-pg, precore, and total). The mRNA values were normalized against the GAPDH RNA internal control. The data is shown as the mean ± SD (n = 3 per bar), **P < 0.01, ***P < 0.001.

To examine whether TARDBP plays a role in HBV gene expression, we performed gene silencing assays using the human hepatocyte cell line HepG2 with stably expressed NTCP (HepG2-hNTCP) as the HBV infection model^16^. The cells were transduced with lentiviruses containing shRNA targeting TARDBP or scramble control shRNA and selected in puromycin. Reduction in TARDBP expression was confirmed by western blotting and qPCR to be >70% (Fig. 1a). The effect of TARDBP silencing on HBV replication was examined by HBV infection of the cells for 12 days. Downregulation of TARDBP resulted in decreased levels of HBV secreted virions (Fig. 1b). Concordantly, knockdown of TARDBP also inhibited secretion of HBs and HBe proteins in the supernatant (Fig. 1c), production of the core protein (Fig. 1d), as demonstrated by CLEIA and western blotting, respectively, and production of HBV replicative intermediates as shown by both real-time PCR and Southern blotting (Fig. 1e). In addition, the 3.5-kb mRNA, which consists of precore and pregenomic (pg) mRNAs together with total HBV mRNAs were also remarkably reduced in TARDBP depleted cells (Fig. 1f). To further elucidate the role of TARDBP in HBV replication, we used T23 cells, a HepG2 cell line that is stably transfected with the 1.4x genome-length HBV expressing plasmid pTRE-HB-wt, and thus can constitutively produce HBV DNA in the supernatant^17^. Consistent with the results of the HBV infection model, gene silencing of TARDBP significantly inhibited expression of the core protein (Fig. 2a), extracellular DNA (Fig. 2b), intracellular DNA (Fig. 2c) and HBV mRNAs (Fig. 2d) in these cells. Taken together, these results indicate that silencing of TARDBP represses HBV transcription.

### TARDBP stimulates the activity of the HBV core promoter

**Figure 3:**
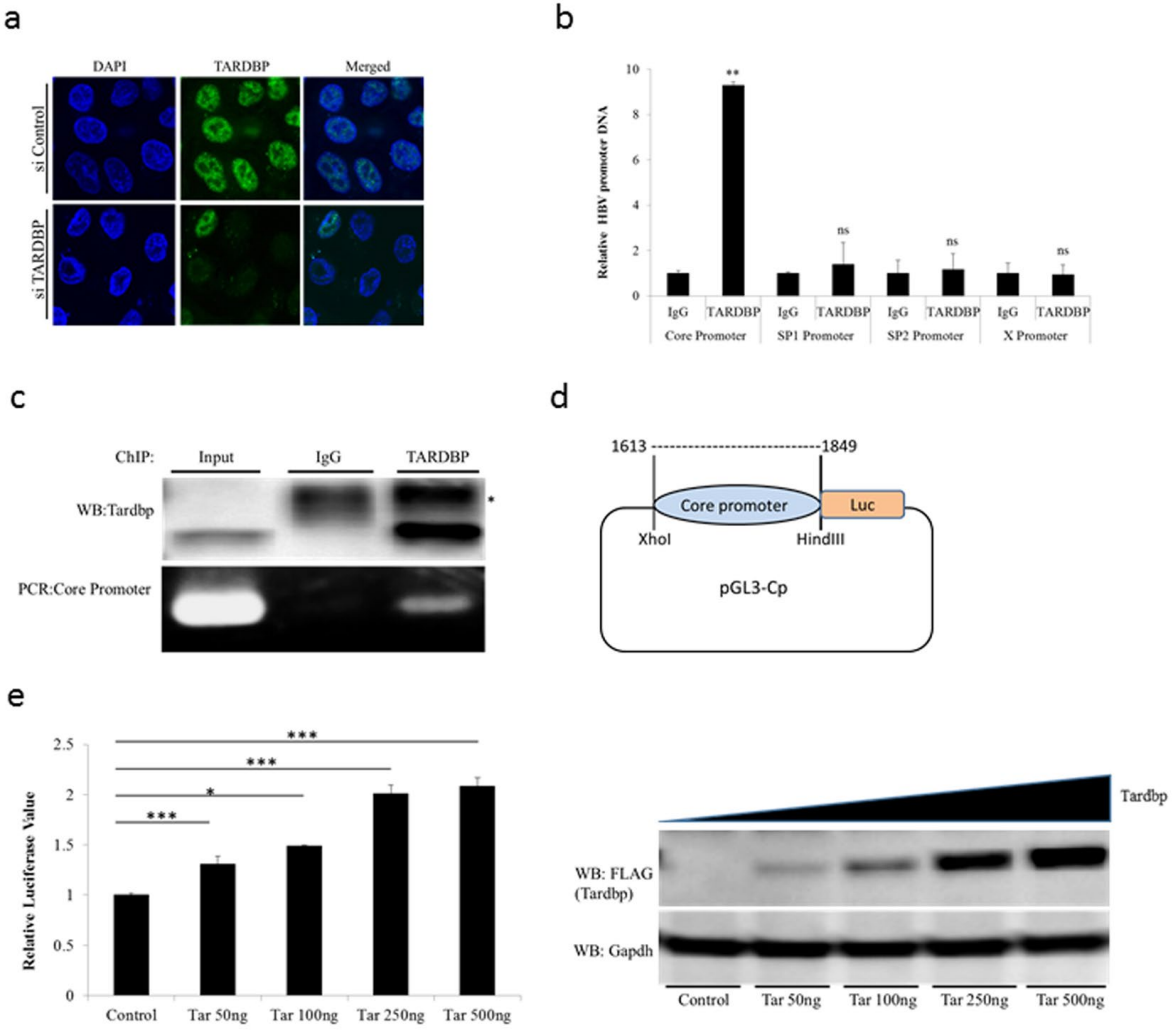
TARDBP activates the HBV core promoter. (**a**) Endogenous TARDBP was detected by immunofluorescence of the HBV expressing cell line T23. The protein was visualized using the anti-TARDBP polyclonal antibody and anti-Rabbit IgG (Alexa Fluor® 488) secondary antibody. Cells were treated with control siRNA or siRNA specific to TARDBP to confirm specificity of the antibody. (**b**) The T23 cells were harvested at confluency and analyzed by ChIP assay using the antibody against TARDBP or the control rabbit IgG. Immunoprecipitated DNA was analyzed in triplicate by qPCR with primers specific for each of the HBV promoter DNA sequences (Core, Sp1, Sp2 and X). The results are displayed as the ratio of the amount of DNA bound to the TARDBP antibody to that bound to the control antibody, with the amount bound to the control antibody set to one. (**c**) To confirm precipitation of TARDBP and core promoter by the antibody, western blotting was performed using the TARDBP antibody, whereas the presence of core promoter DNA was detected by PCR using the core specific primers. The asterisk (*) indicates the location of the IgG heavy chain bands. (**d**) A schematic representation of the luciferase reporter plasmid pGL3-Cp that contains the luciferase reporter whose expression is controlled by the core promoter. (**e**) The pGL3-CP plasmid was co-transfected with the control vector pcDNA3.1/FLAG or increasing concentrations of pcDNA3.1/TARDBP-FLAG into NTCP-HepG2 cells as indicated. The pRL-TK plasmid, which expresses Renilla luciferase, was also included to monitor the transfection efficiency. Cells were lysed at 48 hours after transfection for the luciferase assay. Firefly luciferase values were normalized with control Renilla luciferase activity. The results are expressed as relative luciferase value, which refers to differences (n-fold) from the control value, which is set at one. The protein expression of TARDBP in the lysates was confirmed by western blotting using the anti-FLAG antibody. Error bars represent the SD of three independent experiments. *P < 0.05, **P < 0.01, ***P < 0.001 and ns-non significant.

TARDBP is an RNA/DNA binding protein with predominantly nuclear localization; however, the protein has a nuclear export signal (NES) and a nuclear localization signal (NLS) through which it is believed to shuttle between the nucleus and the cytoplasm^18^. To study its role in HBV, we first investigated the localization of the protein in the stably transduced HBV cell line T23. As shown by immunofluorescence, TARDBP is primarily localized in the nucleus (Fig. 3a), hence its functions are expected to be restricted to the nucleus in our experimental setting. TARDBP was originally identified as a transcription factor that could repress the transcription of HIV-1^11^; moreover, the protein has been shown to bind to DNA sequences in the promoter regions of a number of genes and regulate their expression^19,20^. We hypothesized that it could interact with one or more of the HBV promoters in order to regulate HBV replication. To this end, chromatin was prepared from the T23 cells and subjected to quantitative chromatin immunoprecipitation (ChIP) using a TARDBP antibody. The precipitated DNA was then subjected to real-time qPCR using primers specific for each of the four HBV promoters (Core, Sp1, Sp2 and X). PCR analysis showed that a DNA sequence corresponding to the core promoter was enriched in the TARDBP-antibody precipitate compared to the control, whereas the amount of the other three promoters was unchanged (Fig. 3b). The existence of a band corresponding to enriched TARDBP protein and the core promoter in the anti-TARDBP precipitate was validated by western blotting and PCR, respectively (Fig. 3c). This finding implied that TARDBP could regulate HBV transcription by interacting with the core promoter. To further characterize the relevance of the TARDBP-core promoter interaction, we employed a luciferase reporter assay. The pGL3-Cp plasmid is a luciferase plasmid whose expression is driven from the core promoter (Fig. 3d). The pGL3-Cp was co-transfected with an empty vector or with increasing concentrations of a TARDBP expressing plasmid in NTCP-HepG2 cells. The transcriptional activity was examined in these cells. As shown, we observed a dose dependent enhancement of core promoter activity by TARDBP (Fig. 3e). At this point, we asked ourselves whether TARDBP could also bind to HBV cccDNA. Results from a ChIP assay demonstrated that TARDBP could not precipitate cccDNA, as opposed to HBV core protein, which has already been reported to bind to cccDNA^21^ (Fig. S5), implying that TARDBP binds to cccDNA during active transcription and is therefore easily nicked and degraded during the extraction process. Taken together, these findings suggest that TARDBP interacts with the core promoter, resulting in a positive regulatory effect.

### Establishment of TARDBP-knockout (KO) NTCP-HepG2 cells by the CRISPR/Cas9 system

**Figure 4:**
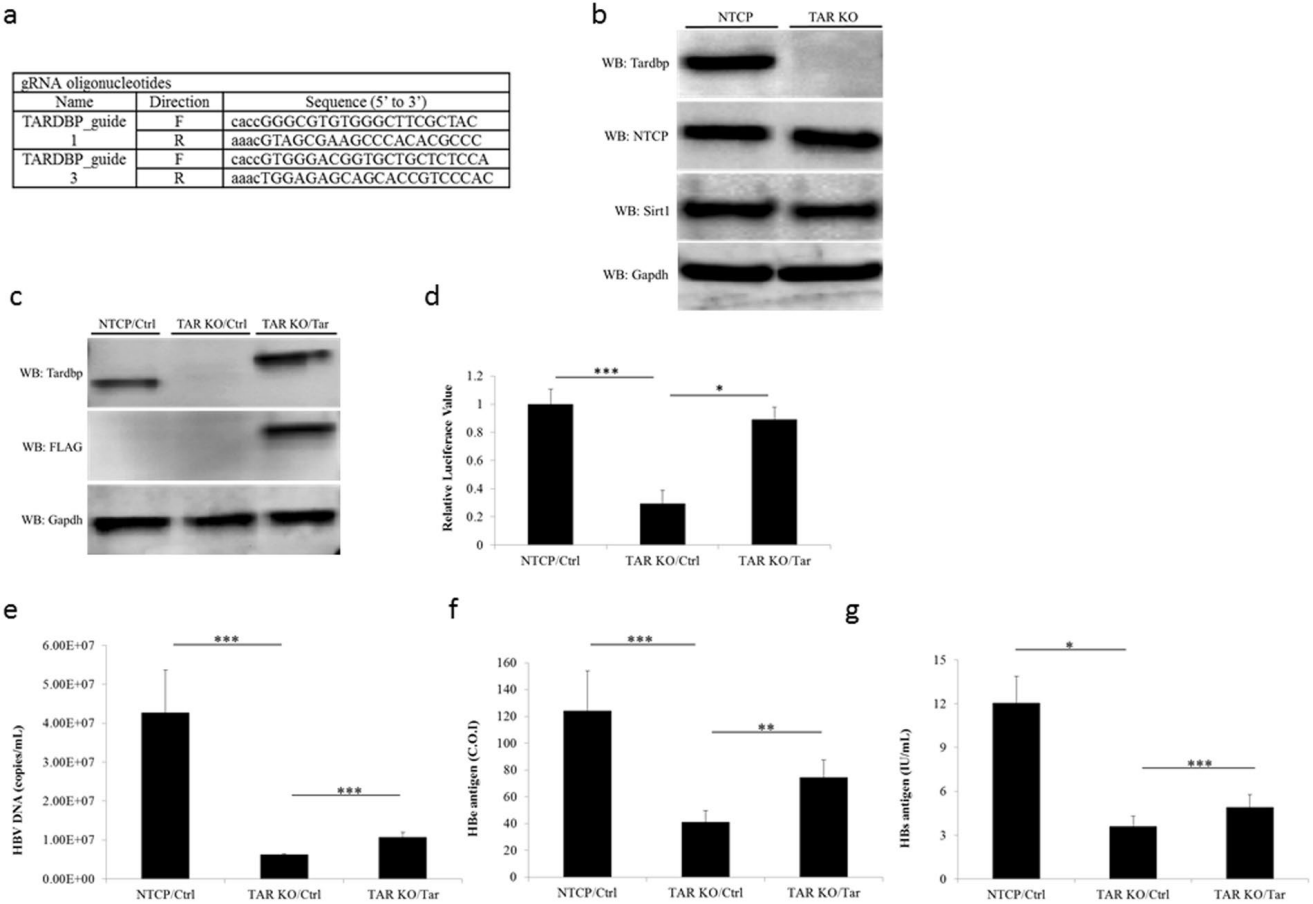
Establishment of TARDBP-knockout (KO) cells by a CRISPR/Cas9 system. (**a**) A TARDBP-deficient NTCP-HepG2 cell line (TARDBP KO) was established by the CRISPR/Cas9 system using the two RNA guide sequences shown. (**b**) Western blot analysis of the NTCP-HepG2 parent cell and the TARDBP knockout NTCP-HepG2 cell line using the antibodies against NTCP, TARDBP, SIRT1 and the control GAPDH is shown. (**c**) A TARDBP plasmid was transfected into the KO cells for exogenous protein expression. The expression was confirmed by western blotting using the anti-TARDBP and anti-FLAG antibodies. (**d**) NTCP-HepG2 parent cell, TARDBP KO and TARDBP KO cells complemented with exogenous TARDBP were transfected with the pGL3-CP plasmid for comparison of the core promoter activities. Cell lysates were harvested after 48 hours for the dual luciferase assay as in Fig. 3e. (**e**,**f**,**g**) HBV infection was carried out in the three cells treated as in (**c**) for 12 days, similar to the experiment in Fig. 1. The supernatants were harvested at the end of the infection period for measurement of extracellular HBV DNA, HBe antigen and HBs antigen levels, respectively. The data is shown as the mean ± SD, *P < 0.05 **P < 0.01, ***P < 0.001.

To further examine the roles of TARDBP on viral replication, we established a TARDBP-deficient NTCP cell line (TARDBP KO) by the CRISPR/Cas9 system using two guide RNAs (Fig. 4a). We confirmed that KO of TARDBP did not affect the expression levels of NTCP, the protein responsible for HBV infection in this cell line, nor another known HBV transcription factor, SIRT1 (Fig. 4b). At first, we examined the core promoter transcriptional activity in the KO cells versus the parental cells. The pGL3-Cp plasmid was transfected in equal amounts to the cells, and luciferase activity was determined 48 hours after transfection. Core promoter transcriptional activity was compromised in KO cells in comparison to the parental NTCP-HepG2 cells, but interestingly, the transcriptional activity was rescued upon ectopic expression of TARDBP (Fig. 4c,d). This observation implied that failure of the cells to activate the core promoter was due to the absence of TARDBP. We next evaluated HBV infection and replicative ability in the KO cells relative to the parental cells. The cells were infected with the HBV virus as in Fig. 1, and HBV-secreted virions and proteins were measured at the end of the infection period. Consistently, HBV gene expression was drastically reduced in the TARDBP KO cells in relation to cells expressing the protein but was partially rescued by the exogenous protein (Fig. 4e–g). Although complementation with ectopic TARDBP exhibited total rescue of the core promoter activity in KO cells, HBV gene expression after HBV infection was only partially rescued. This could be due to the challenge in re-expressing the protein in KO cells for the entire infection period. Whereas the luciferase transcriptional assays were performed 48 hours after transfection when protein expression was still intact, the longer period of time required for HBV infection may have compromised the expression of the transiently transfected plasmid. Indeed, a time series assessment confirmed that the protein was only detected until 72 hours after transfection, beyond which the expression slowly became undetectable (Fig. S2B). Our attempts to establish stably-transfected TARDBP KO cells also failed. The reason for this was not clear, but TARDBP is known to control its own homoeostasis in human cells, as ectopic expression of the protein promoted instability of both the endogenous and ectopic mRNAs^22^. Moreover, other members of the hnRNP family have been shown to possess these auto-regulatory mechanisms^23,24^. This could explain our difficulties in expressing the protein. However, it is still interesting to note that the initial presence of the protein alone was responsible for a significant restoration of the HBV gene expression in KO cells.

### The RNA Recognition Motifs (RRMs) of TARDBP are crucial to activate the core promoter

**Figure 5:**
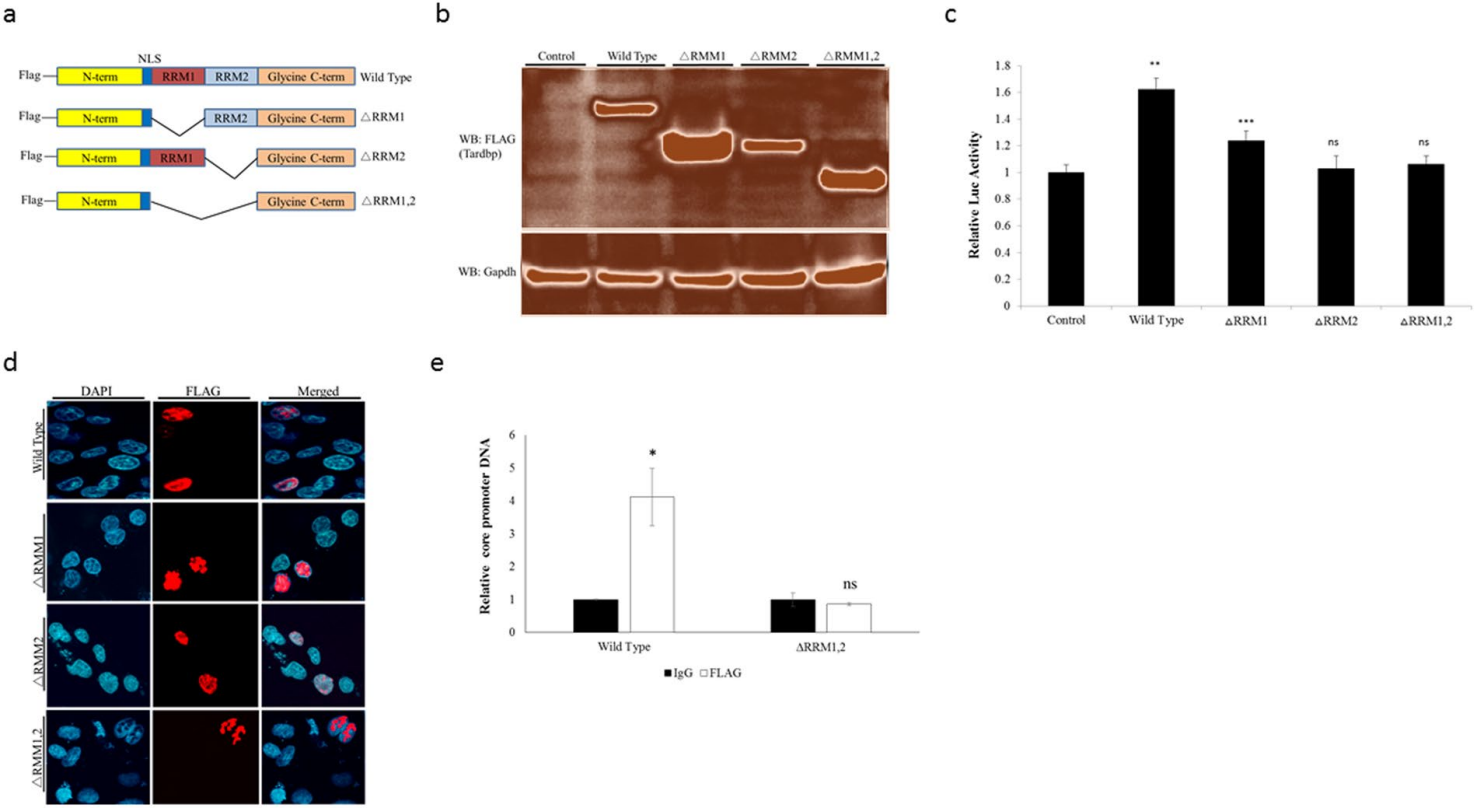
The RNA recognition motifs (RRMs) of TARDBP are crucial for activation of the core promoter. (**a**) A schematic structure of the wild type (WT) TARDBP showing the location of the FLAG tag, the two RRMs (RRM1 and RRM2) and the nuclear localization signal (NLS). Alongside are the three mutants with deletion of RRM1 (ΔRRM1), RRM2 (ΔRRM1) or both RRM1 and 2 (ΔRRM1, 2). (**b**) Each of the four plasmids or the control empty vector was transfected into NTCP-HepG2 cells, and after 48 hours protein expression was determined by western blotting using an anti-FLAG antibody. (**c**) To compare the effect of these proteins on the core promoter activity, the pGL3-CP plasmid was transfected alongside equal amounts of the control plasmid, WT TARDBP, or each of the three RRM mutant plasmids into NTCP-HepG2 cells. Luciferase activities were measured at 48 hours after transfections. Relative values were calculated as described in Fig. 3e. (**d**) For intracellular localization of the proteins, the plasmids were transfected as in (**b**) above. After 48 hours, the cells were treated with the anti-FLAG antibody and visualized by immunofluorescence microscopy. The nucleus was visualized with DAPI. (**e**) To confirm that the two RRMs play a role in interaction of TARDBP with the core promoter, the FLAG tagged WT and the ΔRRM1, 2 mutant proteins were expressed in T23 cells, followed by a ChIP assay as in Fig. 3b using control and anti-FLAG antibodies. The resulting DNA was analyzed with the core promoter primers by qPCR. The relative values were normalized to the amount of DNA precipitated by the control which, was set at one. Results are reported as mean ± SD, *P < 0.05, **P < 0.01, ***P < 0.001 and ns-non significant.

Our results to this point were not sufficient to determine whether the interaction of TARDBP and core promoter is direct or indirect. As noted earlier, TARDBP has two RNA Recognition Motifs (RRM1 and RRM2) through which it is able to bind to DNA and RNA targets. It is believed that both RRMs play a role in the nucleic acid binding by the protein^25^. In addition, the protein has a glycine rich C-terminal region through which it interacts with other proteins^14^. We therefore reasoned that TARDBP could either directly bind to the DNA sequences in the core promoter region or it could indirectly activate the core promoter through interaction with transcription factors that are known to bind and activate the core promoter. If TARDBP binds to the core promoter directly, then disruption of the RRMs could inhibit its transcriptional activity. To this end, we employed three TARDBP mutants in which either or both RRMs were deleted (Fig. 5a). Expression of these proteins in NTCP-HepG2 cells was confirmed by western blotting using the anti-FLAG antibody (Fig. 5b), as they all contain the FLAG tag at the N-terminus. As a next step, we used the luciferase reporter assay to compare core promoter transcriptional activation by the three RRM mutant proteins versus the wild type protein. The ability of TARDBP to activate transcription from the core promoter was partially disrupted by deletion of RRM1 but was totally abolished by deletion of RRM2 or both RRMs (Fig. 5c). Immunofluorescence microscopy confirmed that each of the proteins maintained nuclear localization (Fig. 5d), indicating that interference with the transcriptional effect was not due to nuclear translocation. To further confirm the significance of the two RRMs in HBV DNA binding, we used the TARDBP ΔRRM1, 2 mutant, in which both nucleic acid binding domains were deleted for the ChIP assay. We confirmed that deletion of the two RRMs abolished the ability of the protein to interact with the core promoter (Fig. 5e). Taken together, our results imply that the RRM2 plays a dominant role, with RRM1 having a supportive role in the protein’s transcriptional effect. Moreover, deletion of both RRMs totally abrogates the interacting ability of the protein and subsequent transcriptional effect, which confirms that the DNA binding ability of the protein is necessary for it to activate the core promoter.

### TARDBP binds to a sequence within the core promoter of HBV

**Figure 6:**
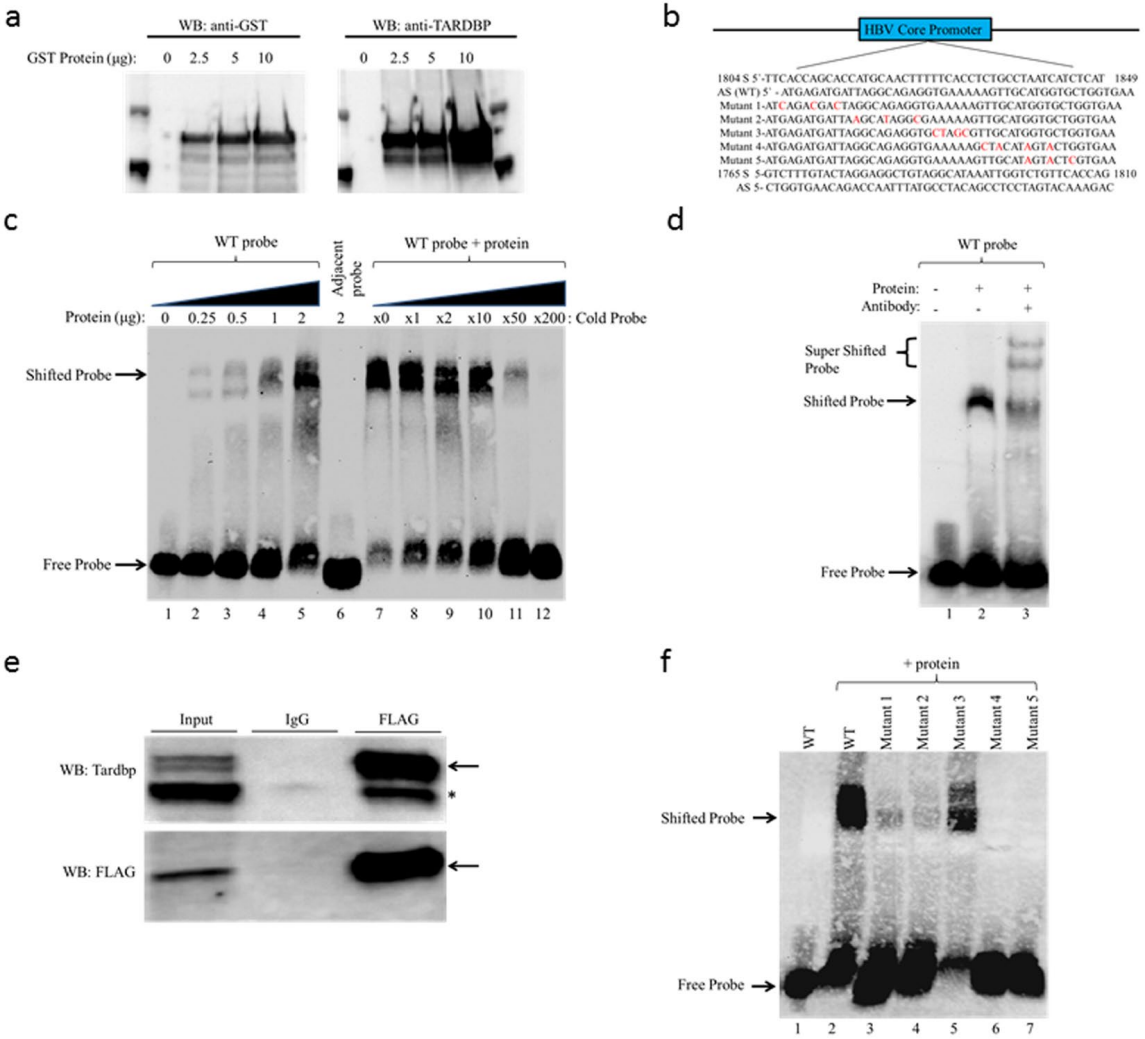
Detection of TARDBP-core promoter interaction in an electrophoretic mobility shift assay (EMSA). (**a**) Western blot analysis of the recombinant GST-fused TARDBP protein purified using glutathione agarose beads. The protein was detected using antibodies directed against the GST tag and TARDBP protein. (**b**) The nucleotide sequence of TARDBP binding site within the HBV core promoter region is shown (nt1804-nt1849). The sense (S) and antisense (AS) probes of the sequence, together with the five AS mutant probes (Mutant 1–5), and the S and AS sequence of the adjacent probe (nt1765-nt1810) used for the EMSA assay are indicated. The nucleotide changes in the mutants are marked in red. (**c**) The AS (WT) probe was incubated alone (lane 1) or with increasing concentrations of TARDBP protein (lanes 2–5); the adjacent AS probe was incubated with 2 μg of TARDBP (lane 6); the AS (WT) probe was incubated with 2 μg of TARDBP alongside increasing concentrations of an unlabeled specific competitor at 0, 1, 2, 10, 50 and 200 fold respectively, (lanes 7–12). (**d**) A supershift assay was performed with an anti-TARDBP antibody; lane 1, AS (WT) probe alone; lane 2, AS (WT) probe with TARDBP protein; lane 3 AS (WT) probe with TARDBP protein along with 2 μg rabbit anti-TARDBP antibody. The specific TARDBP-DNA complex (shifted probe arrow), and the antibody-TARDBP-DNA complex (super shifted probe arrow) is indicated. (**e**) The HBV-producing T23 cells were transfected with 5 μg of FLAG tagged TARDBP, immunoprecipated with an anti-FLAG antibody and blotted with anti-FLAG and anti-TARDBP antibodies. The arrow shows the location of the exogenous protein, and the asterisk (*) shows the endogenous protein which was also precipitated by the FLAG-antibody. (**f**) Starting at the N-terminus, mutations were introduced at five sites in the AS (WT) probe to make five different mutant probes, Mutants 1–5, as shown in (**b**). The WT probe was incubated alone (lane 1) or in the presence of 2 μg of TARDBP protein (lane 2), while each of the five mutant probes was also incubated with the equivalent amount of TARDBP protein (lanes 3–7). The location of the free probe and the specific TARDBP-DNA complex (shifted probe) are indicated by arrows.

TARDBP has been shown to bind to relatively long DNA sequences; one group demonstrated binding of the protein to a 38 bp sequence of the SP-10 gene promoter^26^, whereas another study showed that it binds to a 43 bp sequence of the HIV TAR DNA^11^, and yet another study confirmed that it binds to a 66 bp sequence of the TNF-α gene promoter^27^. The minimum sequence necessary for DNA binding by TARDBP remains unknown. Since the core promoter spans the region nt1613-nt1849 of the HBV viral genome^28^, we randomly selected an approximately 46 bp target length to create 6 overlapping DNA probes from the entire 237 bp region for the EMSA experiment. Because TARDBP has been shown to sometimes bind to the antisense sequence but not the corresponding sense sequence^26^, we also included the antisense probes for each of the 6 probes to make a total of 12 probes. The probes were labeled as sense (S) probes 1–6 and anti-sense (AS) probes 1–6 in a consecutive manner starting from the C-terminus (Fig. S3). To screen for sequences among these probes to which TARDBP could bind, EMSA was performed using a GST-purified TARDBP recombinant protein that was successfully detected by anti-GST and anti-TARDBP antibodies (Fig. 6a). In the EMSA experiment, two shifted bands, indicating a possible DNA-protein interaction, were observed for 2 of the 12 probes, namely S probe 3 and AS probe 1 (Fig. S4A, lanes 4 and 8). However, further experimentation indicated that binding to the S probe 3 was only dependent on the formation of a secondary structure whose intensity was reduced in the presence of the protein, suggesting that the protein bound to the secondary structure and not the intact probe (Fig. S4A, lane 15 and Fig. S4B lane 5). Henceforth, we decided to focus on the AS probe 1 that indicated direct protein binding (Fig. S4B, lane 2). The probe was the antisense strand of nt1804-nt1849 whose sequence is shown in Fig. 6b. Indeed we confirmed binding of TARDBP to the probe, as the intensity of the shifted band increased in proportion to the dose of the protein (Fig. 6c, lanes 1–5). A similar pattern was observed when the probe was incubated with decreasing concentrations of a specific competitor (Fig. 6c, lanes 7–12). To rule out the possibility of non-specific binding, we tested binding of TARDBP to the adjacent AS probe of nt1765-nt1810 whose sequence is also shown in Fig. 6b. The protein failed to bind to the probe from the adjacent sequence, showing that the interaction was specific (Fig. 6c, lane 6). To further confirm binding of TARDBP to the 46 bp element, the EMSA reaction was performed in the presence of a TARDBP antibody. Indeed the antibody did cause a supershift (Fig. 6d, lane 3). However, we noticed that two super-shifted bands were present. We reasoned that this could be due to the presence of homodimers, as TARDBP molecules are known to fold and homodimerize^14,29^. To resolve this point, we immunoprecipitated a FLAG-TARDBP cell lysate using an anti-FLAG antibody followed by immunoblotting with an anti-TARDBP antibody, as exogenous tagged-TARDBP is known to appear at a slightly higher molecular weight than the endogenous one^30^. Indeed we observed two bands, one corresponding to the exogenous (FLAG-tagged) protein and the other to the endogenous protein (Fig. 6e) confirming self-interaction of TARDBP molecules. Altogether, the observations confirm that TARDBP directly binds to the core promoter DNA.

### TARDBP has two critical binding sites on the core promoter of HBV

To identify the elements within the 46 bp probe of the core promoter region that represent the specific targets for TARDBP binding, we first tested binding to four overlapping truncated fragments of the probe, namely s1–s4 (Fig. S4C). The wild-type probe interacted with TARDBP as expected (Fig. S4D, lane 2); however, under the same conditions, none of the four fragments could interact with the TARDBP protein (Fig. S4D, lanes 4, 6, 8 and 10). As a next step, we used mutational analysis to scan the sequence for possible target regions based on previous reports. TARDBP has been shown to bind preferentially to DNA enriched for TT, TG or GT nucleotide pairs^31^. We identified these sequences at several locations within the probe. To study their significance, we generated a series of five mutant probes (mutant 1–5) where individual nucleotides were substituted, focusing specifically on locations with TT, TG or GT pairs (Fig. 6b). The mutants were subjected to an EMSA assay alongside the wild type probe. As shown, mutation of the central part containing a five A-nucleotide repeat did not affect binding of TARDBP to the probe (Fig. 6f, lane 5). However mutations of T or G nucleotides abolished the binding of the protein to the probe (Fig. 6f, lanes 3, 4, 6 and 7). Consistent with previous reports, TG and GT nucleotide pairs seem to play a central role in TARDBP binding to the core promoter. In addition, the results also show that TARDBP has two separate binding sites on the probe, as mutations in either the N-terminal or the C-terminal sequences abolished the interaction. Taken together, our results demonstrate that TARDBP has two binding sites on the core promoter and both of them are necessary for the interaction.

### TARDBP assembles protein complexes that support the HBV virus in the nucleus

**Figure 7:**
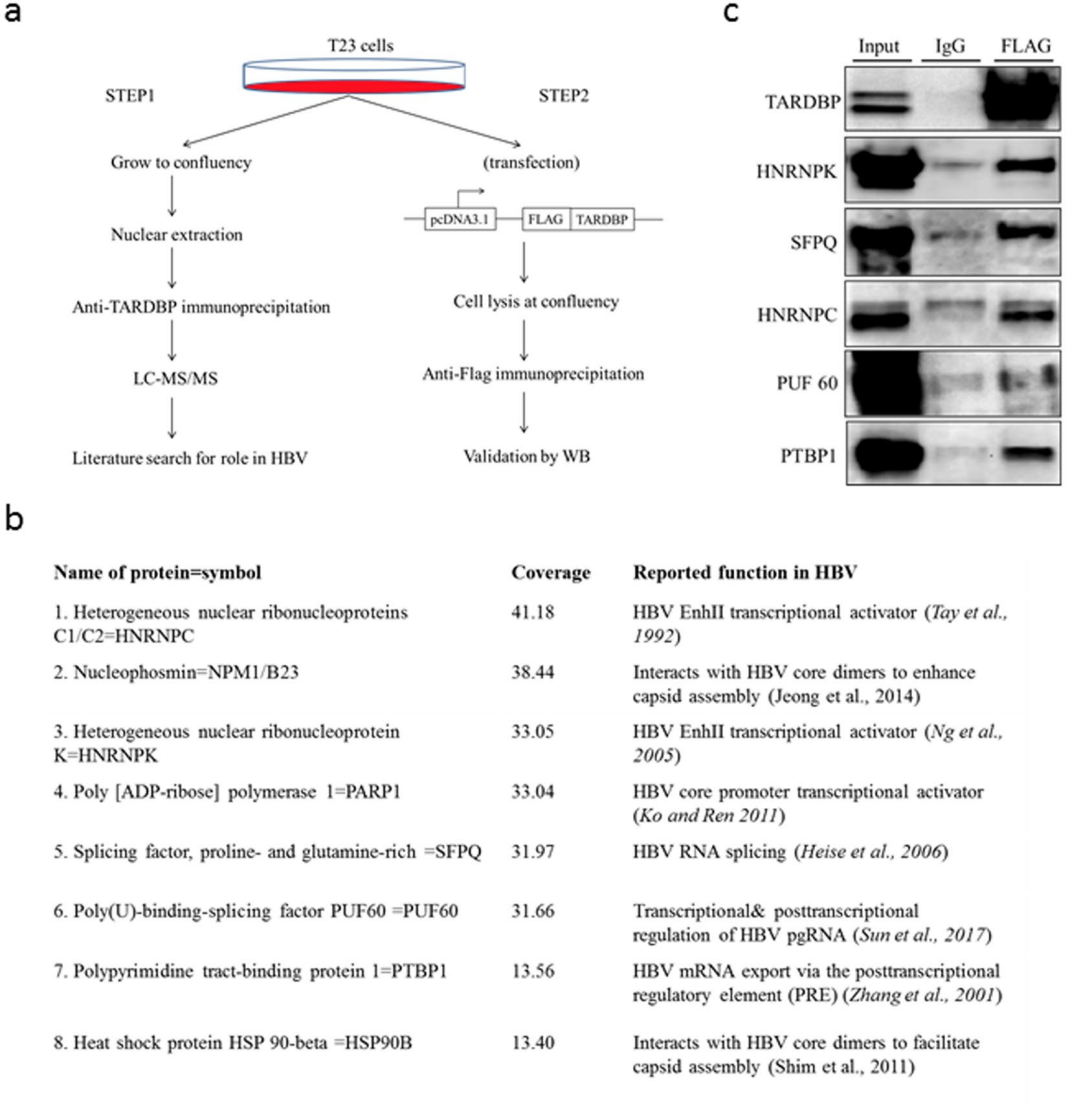
TARDBP assembles protein complexes that support the lifecycle of HBV. (**a**) An experimental design for the identification of TARDBP-interacting proteins. In the first step, nuclear lysates were prepared from the HBV producing cell line T23, immunoprecipitated by a TARDBP antibody and subjected to LC-MS/MS analysis. A literature search was done on the top scorers to ascertain which of them likely played a role in the HBV life cycle. In the second step, interactions of the candidate proteins *in vivo* were validated by western blotting using the same cell line as in (a) but with an exogenously expressed TARDBP protein. (**b**) The set of 8 proteins that scored a coverage of >10 on the LC/MS-MS analysis and were found from literature to have a role in HBV replication. The figure includes the specified role and the literature associated with each protein. (**c**) Nuclear lysates of the T23 cells expressing FLAG-tagged TARDBP were precipitated by the anti-FLAG antibody or the control mouse IgG and subjected to western blotting for each protein using their specific antibodies as shown.

As mentioned earlier, aside from the nucleic acid binding properties of TARDBP, another important aspect of the molecule is the ability to interact with other proteins via the glycine-rich domain at the C-terminus (Fig. 5a). We therefore wondered whether HBV may take advantage of this in addition to the nuclear localization of the protein to assemble protein complexes that favor viral replication. We first screened for proteins that interact with TARDBP in a HBV replicating cell line using LC-MS/MS (Fig. 7a) and identified 491 potential interaction partners (Table S3). Coverage ranged from 0.17 to 63.86 with a median of 8.45. The peak list included 25 transcription factors, 17 oncogenes, 3 protein kinases, and 3 cell differentiation markers. Gene set enrichment analysis of canonical pathways indicated significant enrichment of splicing, poly(A) RNA binding, and RNA processing pathways. To identify any proteins that could play a part in the HBV lifecycle, we selected the top proteins with coverage 10 or above and searched the literature using these proteins and HBV as search terms. Following this approach, 8 proteins were identified, namely PTBP1, PUF60, SFPQ, Hsp90, PARP1, HNRNPK, NPM1, and HNRNPC (Fig. 7b), which have been reported to play various roles in transcription and post-transcriptional and nucleocapsid assembly stages of the virus^32–39^. To verify these interactions *in vivo*, we immunoprecipitated a nuclear lysate of the same cells expressing FLAG-tagged TARDBP by an anti-FLAG antibody, followed by western blotting to detect proteins. Out of the 8 proteins, 5 were confirmed to interact with TARDBP (Fig. 7c). Altogether our results suggest that HBV benefits from the ability of TARDBP to assemble diverse proteins that support the viral life cycle in hepatocytes.

### TARDBP regulates HBV pgRNA splicing

**Figure 8:**
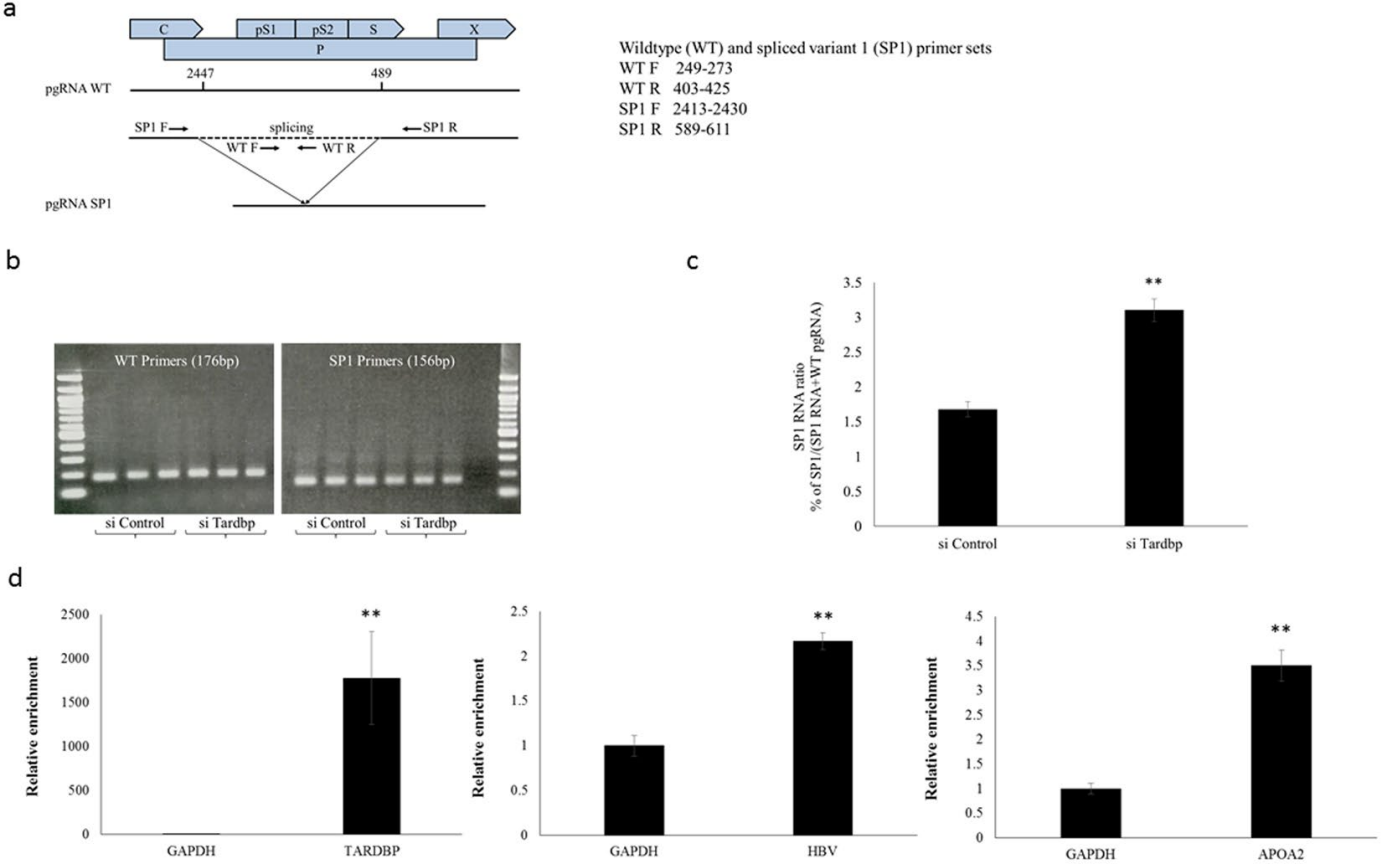
TARDBP regulates HBV pgRNA splicing. (**a**) A schematic representation of the HBV genome indicating the site of splicing for the major splice variant, SP1 (intron 2447/489), and the primers designed for PCR amplification of the wild type (WT) and SP1 pgRNA. (**b**) The T23 cells, which stably express HBV, were transfected in triplicate with siRNAs against TARDBP or control. Total RNA was extracted from the cells, followed by reverse transcription. The resulting cDNAs were analyzed by PCR followed by agarose gel electrophoresis with primers specific for WT pgRNA or SP1 RNA. The results confirmed that there was no non-specific amplification of WT products with SP1 primers and that the two products were at the expected sizes. (**c**) The primers in (**b**) were henceforth utilized in a quantitative PCR of the samples to determine the proportion of SP1 RNA, which was calculated as SP1 RNA/(SP1 pgRNA + WT pgRNA). (**d**) Whole cell lysate from FLAG-TARDBP or FLAG-transfected T23 cells was immunoprecipitated with an anti-FLAG antibody and subjected to isolation of RNA and qPCR analysis using primers for TARDBP, APOA2 and total HBV mRNAs. The amount of mRNA that was precipitated from the TARDBP transfected cells was calculated as a ratio relative to that bound to the control-transfected cells for each of the primers. Relative mRNA values are presented as a ratio with respect to the amount of GAPDH mRNA, which was set at one. Results are reported as mean ± SD, while **P < 0.01.

TARDBP is well known to be involved in RNA splicing^12^. In addition, most of the TARDBP-interacting proteins identified in Fig. 7 have been reported to be involved in mRNA splicing events^40–44^. We therefore investigated whether TARDBP could play a role in HBV mRNA splicing. HBV undergoes reverse transcription during its replication and only utilizes unspliced mRNA for viral gene expression^45^. In addition to the unspliced mRNAs, a series of spliced (SP) HBV RNAs have been widely described in model systems and in HBV-infected livers^46^. The most frequently detected variant is a 2.2 kb molecule termed SP1, which is generated through the removal of a 1.3 kb intron from the pgRNA at nt2447 and nt489^45^. To determine any role for TARDBP in HBV pgRNA splicing, we measured the ratio of SP1 to the WT pgRNA in HBV producing cells with or without silencing of TARDBP. We employed two sets of primers to recognize intron-internal sequences to detect the WT product and those across the exons to detect the SP form (Fig. 8a). Specificity of the primers was confirmed, as SP primers did not amplify the WT product (Fig. 8b). As shown, diminishing TARDBP in the cells resulted in 100% increase in splicing of pgRNA (Fig. 8c), which implies that TARDBP serves as an inhibitor of splicing, thereby enhancing the export of unspliced pgRNA into the cytoplasm. As a next step, we tested whether TARDBP could bind to HBV RNA. To this end, total lysates were obtained from HBV-producing cells that were over-expressing FLAG-tagged TARDBP. They were then subjected to an RNA immunoprecipitation assay using TARDBP antibody as the bait. The precipitated mRNAs were purified and subjected to qPCR analysis to detect HBV mRNA. TARDBP and APOA2 mRNAs served as positive controls since they are already known to bind to TARDBP^22,47^. GAPDH mRNA was detected as a negative control to exclude non-specific interactions. As expected, TARDBP and APOA2 mRNAs were enriched on the TARDBP antibody (Fig. 8d). In addition, total HBV mRNA was also shown to be enriched on the TARDBP precipitate, indicating that the mRNA was precipitated by the protein (Fig. 8d). As a next step, we attempted to identify potential TARDBP HBV RNA binding sites by examining the HBV genome for conserved TG-repeats. We downloaded aligned genome sequences from HBVdb and performed a regular expression search for (TG)+ repeats. While we found a number of clusters of repeated T or G nucleotides throughout the HBV genome, we found few conserved TG stretches with more than two repeats (Fig. S6A). However, experimentally confirmed TARDBP RNA binding sites typically indicate some degree of flexibility in this basic pattern (Fig. S6B). Therefore, we performed a fuzzy search in which gaps were penalized over mismatches for each of the 11 experimentally determined TARDBP RNA-binding motifs listed in the RBPDB database of RNA-binding protein specificities. We uploaded the highest scoring matches in BED format to the NCBI Graphics panel for the HBV reference sequence KR819180.1. We observed a number of matches within the X transcript overlapping the core promoter/enhancer II region and found other clusters within the S and core transcripts, as well as a match downstream of the polyadenylation site (Fig. S6C). These results suggest that TARDBP might interact with HBV transcripts within the 3′ non-coding regions shared by all transcripts and might interact with HBV RNA in the same region as the DNA binding site in the core promoter/enhancer II region. In the future, we will attempt to experimentally confirm these potential RNA interaction sites and determine their role in HBV replication. In all, our data shows that in addition to DNA, TARDBP is also able to interact with HBV mRNA and, consequently, plays a role in inhibition of pgRNA splicing to enhance HBV gene expression.

## Discussion

In this study we demonstrate for the first time that TARDBP binds both to HBV DNA as well as RNA and functions as a transcriptional activator through binding to the core promoter and as a splicing suppressor through binding to pgRNA. TARDBP was originally identified as a cellular factor that binds strongly to double-stranded HIV-1 TAR DNA to repress transcription from the HIV-1 LTR^11^. Subsequent studies confirmed this role for the protein as a transcriptional repressor^13,19,20^. However, more recent research has demonstrated that TARDBP can indeed also work as a transcriptional activator by binding to the LPS-responsive element in the TNF-α promoter to increase TNF-α expression^27^. Our study provides several lines of evidence that TARDBP acts as a positive regulator of HBV. First, silencing of TARDBP diminished HBV gene expression. Secondly, TARDBP could activate transcription from the HBV core promoter. Thirdly, HBV benefits from the nuclear localization of TARDBP and its ability to assemble protein complexes that are already known to support various stages of viral replication. Finally, the protein served an inhibitory role on pgRNA splicing, which is likely to facilitate nuclear export of the non-canonical unspliced viral RNAs for HBV gene expression.

Known HBV-associated transcription factors either bind to the core promoter directly or regulate transcription indirectly as co-activators or co-repressors via interaction with other molecules. For instance, several host transcription factors have been reported to indirectly activate the HBV core promoter, such as SIRT1, Cyclin D2 and jumonji C domain-containing 5 (JMJD5)^48–50^. On the other hand, many other transcription factors bind directly to the core promoter, including CAAT enhancer-binding protein alpha (CEBPA), hepatocyte nuclear factor 4 (HNF4), chicken ovalbumin upstream promoter transcription factor (COUP TF)^51^, among others. The present study adds a new transcription factor to this list, as we observed direct binding of TARDBP to the core promoter, resulting in a positive effect on transcription. Our findings confirm prior reports that TARDBP can function both as a transcriptional repressor and as an activator. This pattern has been reported for Spindlin1, a protein that could transcriptionally repress HBV in the context of infection by binding to HBV cccDNA, whereas previous research had focused on its role in transcriptional activation^52,53^. The molecular mechanisms responsible for both transcriptional activation and repression by TARDBP remain unreported. However, it is possible that the molecular switch between repressor and activator functions can be influenced by the infection model or cell type, post-translational modifications, or interactions with different proteins. It will therefore be important to characterize the conditions that favor one transcriptional function of TARDBP over the other.

By performing mutagenesis assays, we demonstrated that deletion of the RNA/DNA binding motifs abolished the transactivation effect of TARDBP on the core promoter. This observation is consistent with previous reports that both domains are necessary for nucleic acid interactions^14^. The motifs were also crucial for the ability of TARDBP to bind to HIV TAR DNA, as deletion of the glycine rich C-terminus had no effect, whereas deletion of the ribonucleoprotein binding motifs abolished the interaction^11^. In our case, we noted that deletion of RRM1 only partially reduced the effect, whereas deletion of RRM2 completely abolished the effect. This is contrary to previous reports in which RRM1 was found to play the dominant role, while RRM2 was dispensable or played only a supportive role^12,25,31^. It is conceivable that both domains are necessary for interaction with DNA, but their respective functions might depend on an unknown factor. On the other hand, while the glycine rich C-terminal domain of TARDBP was not necessary for binding to the core promoter, it still played a separate but important role in supporting HBV replication. As the motif is important for interaction with other proteins, we found a substantial number of factors that are already known to support the HBV viral lifecycle as interacting partners of TARDBP.

Whereas most studies have emphasized the preference of TARDBP for TG-rich DNA sequences^20,26,54^, it has recently become clear that the protein can also interact with other nucleotide motifs^31^. In agreement with Furukawa *et al*., we showed that regions containing TG, GG, TT, and GT nucleotide pairs play important roles in binding, whereas an intervening region with AA pairs was apparently dispensable, although we do not discount the possibility that this region could serve as a spacer sequence and that the nucleotide composition could allow the DNA molecule to flex in order to facilitate the formation of protein complexes. The initial report of TARDBP binding to HIV TAR DNA actually showed that the protein bound to pyrimidine-rich sequences but not to TG repeats^11^. They also demonstrated that two separate groups of pyrimidine-rich residues were crucial for TARDBP binding to TAR DNA, as mutations in either of the residues abolished the binding^11^. Accordingly, we observed that TARDBP failed to bind to the DNA sequence when either of the two target sequences was mutated. KLF15 is another protein which has been shown to bind to the core promoter via two binding sites, for which mutation of one of the sites abolished the binding^55^. While it is not yet known how TARDBP binds to two sites simultaneously, we speculate that two molecules of TARDBP bind to the core promoter in the form of homodimers, as TARDBP molecules are known to bind to one another^29^. In support of this model, our supershift assay demonstrated that two (instead of one) shifted bands existed in the same assay condition. In this scenario, we speculate that in the sample mixture, the antibody bound to either or both of the two TARDBP molecules in the protein-DNA complex, resulting in a single and double shift, respectively. This was further confirmed by an immunoprecipitation experiment where an anti-FLAG antibody precipitated both the FLAG-tagged (exogenous) and endogenous protein.

In this study we showed that DNA binding of TARDBP to the core promoter directly up-regulates HBV transcription. However, TARDBP is a multifunctional protein that can bind to either RNA or DNA and is well known to bind to 3′ UTR regulatory regions or splicing regulatory sites in RNA^56^. TARDBP is also known to interact with a number of proteins, most of which belong to two major functional categories, RNA processing in the nucleus and protein translation in the cytoplasm^57^. Our LC-MS/MS results revealed a large number of potential interaction partners (NPM1, PARP1, Hsp90, HNRNPC, SFPQ, PTBP1, HNRNPK and PUF60), several of which have already been reported to affect HBV replication via, e.g., transcriptional regulation, RNA processing, nuclear export, or nucleocapsid assembly^32–39^. HBV faces several RNA processing challenges, including avoiding RNA degradation, preventing unwanted splicing, and exporting the non-canonical viral transcripts from the nucleus. Export of mRNA from the nucleus normally involves formation of ribonucleoprotein particles and is tightly coupled to splicing; therefore, export of non-canonical unspliced HBV RNA transcripts and pgRNA from the nucleus poses a challenge for the virus and requires recruitment of adapter proteins to facilitate transfer through the nuclear pore. Given the ability of TARDBP to alter or silence RNA splicing and the protein’s capacity to interact with a large number of proteins involved in RNA metabolism, as well as the presence of both nuclear import and export signals, we speculated that TARDBP might play a role in RNA processing and export of HBV transcripts. In support of this hypothesis, we were able to detect HBV mRNA in TARDBP precipitate, suggesting an interaction between HBV RNA and TARDBP. Furthermore, in a recent study, Duriez *et al*. performed RNA pull-down assays followed by LC-MS analysis to detect pgRNA-interacting proteins and identified TARDBP as a potential interaction partner^58^. They observed that 15% of the RNA-interacting proteins were directly associated with splicing and noted that pgRNA splicing results in formation of HBV splicing-generated protein (HBSP), which confers a protective effect against immune-mediated hepatic injury through down-regulation of CCL2 expression. In this study, we demonstrated that TARDBP inhibits splicing of pgRNA in T23 cells and showed that TARDBP knockdown enhanced pgRNA splicing. Further research is necessary to confirm whether TARDBP plays a role in HBV splicing and/or nuclear export, but our current findings suggest that disruption of TARDBP-protein complex formation may yield seeds for anti-HBV drug development.

Briefly, we identified TARDBP as a host factor that facilitates HBV gene expression by stimulating transcription from the core promoter, assembly of protein complexes implicated in transcriptional and post-transcriptional stages of the virus life cycle, and suppressing pgRNA splicing during nuclear export. Our results provide valuable insight into the interaction between host factors and HBV. TARDBP may thus serve as an effective target for novel anti-HBV therapies.

## Materials and Methods

### Plasmids, Oligos, siRNA and shRNA

Full-length TARDBP was amplified from a human brain cDNA library (Clontech) and cloned into the BamHI-HindIII site of pcDNA3.1 (−) (Invitrogen) with a 5′ FLAG tag inserted through the NotI-EcoRI site. The RRM mutants of TARDBP were generated by site-directed mutagenesis and cloned into the same vector as the full length plasmid. The control pcDNA3.1/FLAG plasmid only contains the flag tag at the same location as the TARDBP plasmids. These plasmids were a kind gift from Dr. Philipp Kahle (University of Tübingen, Germany). The pGEX-TARDBP plasmid that was used for GST-tagged protein production and purification for the EMSA experiments was constructed from the above TARDBP plasmid. In brief, a fragment containing human TARDBP CDS was amplified from the pcDNA3.1/FLAG-TARDBP plasmid by PCR using the primers, 5′-TGGTTCCGCGTGGATCCATGTCTGAATATATTCGGGTAACC-3′ and 5′-AGTCGACCCGGGAATTCTACATTCCCCAGCCAGAAGACTT-3′ and ligated into the BamHI-EcoRI site of pGEX-4T-1 vector. For the luciferase experiments, the HBV core promoter (CP) plasmid was constructed by inserting the nucleotides 1613–1849 of the HBV genome (GenBank accession number KR819180) into the pGL3 basic vector (Promega) through the XhoI-HindIII site.

All oligonucleotides used as primers or probes for PCR, EMSA and RNA IP experiments were designed and ordered from Sigma-Aldrich-Genosys, Japan.

Control siRNAs and siGENOME SMART pool siRNAs directed against TARDBP (SMARTpool: siGENOME Human TARDBP siRNA M-012394-01-0005) were purchased from Dharmacon through GE healthcare, JAPAN. The TARDBP target sequences of the 4 siRNAs contained in the pool are: GGCCUUCGGUUCUGGAAAU, GCAAACUUCCUAAUUCUAA, CAAUAGCAAUAGACAGUUA and GCUCAAGCAUGGAUUCUAA).

Control shRNA and shRNA TARDBP (TARDBP MISSION shRNA TRCN0000016040: CCGGGCTTTGGCTCAAGCATGGATTCTCGAGAATCCATGCTTGAGCCAAAGCTTTTT) in the form of Lentiviral Transduction Particles were purchased from Sigma Aldrich, Japan.

Plasmids used in establishment of NTCP-HepG2 knock out (KO) cells were constructed as follows: for the knock-in donor plasmids, the left and right homology arms of the TARDBP gene (Fig. S1A) were amplified by PCR from NTCP-HepG2 C4 cells genomic DNA. Antibiotic resistance ORF (puromycin or blasticidin) together with the set sequence of P2A, DD-tag and 3xGGGGS, which was fully chemo-synthesized (GenScript) according to the published protocol^59^, was inserted between the left and right homology arms of TARDBP genomic sequence (Fig. S1B,C) preliminarily cloned into pBlueScript II SK(+) using In-Fusion (Clontech). The pX330, a human codon-optimized SpCas9 and chimeric guide RNA expression plasmid, was obtained from Addgene (catalog no. 42230). Two sgRNA sequences neighboring to the start codon of TARDBP gene (TARDBP_guide 1 and TARDBP_guide 3 (Fig. 4a) were selected by CRISPR design tool^60^. The pX330 was linearized with Bbs I digestion and two pairs of oligonucleotides for TARDBP targeting sites were annealed and ligated into the Bbs I site of pX330, respectively. Avoiding re-digestion by Cas9 nuclease after desired knock-in occurred on the genome, point mutations with no-change on the amino acid sequence of TARDBP protein were inserted within each PAM sequence of guide1 and guide 3 (Fig. S1B,C) by site-directed mutagenesis.

### Cell culture and treatments

The HepG2 cell line was derived from a human hepatoma cell line. The production of the T23 cell line from HBV-stably transfected HepG2 cells has previously been described^17^. Briefly, HepG2 cells were transfected with the plasmid pTRE-HB-wt by calcium precipitation and the transfected cells were selected. Colonies were isolated, and clones that were positive for both HBs and HBe antigens were selected. Finally, one cell line named T23 was identified and used for further experiments as it was able to continuously produce high copies of HBV DNA in supernatant. The T23 cells were grown in DMEM (Gibco) supplemented with 10% (v/v) fetal bovine serum and 400 μg/ml hygromycin (InvivoGen, CA, USA). NTCP-HepG2 is another cell line derived from the HepG2 line and is stably transfected with NTCP, the membrane transporter necessary for entry of the HBV virus into hepatocytes^5^. The NTCP-HepG2 C4 clone which was used in this study was a kind gift from Dr. Watashi and is highly susceptible to HBV infection^16^. The NTCP-HepG2 cells were cultured with DMEM/F-12 + GlutaMax (Gibco) supplemented with 10% FBS, 10 mM HEPES (Gibco) and 5 μg/ml insulin (Sigma) in the presence of 400 μg/ml G418 (Gibco). All plasmid transfections were carried out using the transit-IT-LT1 transfection medium according to the manufacturer’s recommended conditions (Mirus Bio LLC), while the siRNA transfections were performed using Lipofectamine RNAiMAX transfection reagent according to the manufacturer’s instructions (Invitrogen). Lentiviral particles were transduced into the cells in the presence of 10 μg/mL hexamethrine bromide according to the manufacturer’s recommended conditions (Sigma-Aldrich), followed by selection of positive colonies with 1 μg/ml puromycin (InvivoGen).

### HBV preparation and infection

HBV infection of NTCP-HepG2 C4 cells was performed using HBV genotype C derived from the serum of a chronic HBV patient with high viral load (≥10^9^ copies/ml). The patient agreed to provide blood samples for a viral hepatitis study through written informed consent. The study protocol conforms to the ethical guidelines of the 1975 Declaration of Helsinki and was approved *a priori* by the ethical committee of Hiroshima University. The infection of cells was performed at 200 genome equivalents (GEq)/cell in the presence of 4% PEG8000 at 37 °C and 5% CO_2_ for 16 h as previously described^61^. The infectious serum was then removed from the cells followed by washing three times with PBS and incubation in fresh medium. The subsequent culture medium contained 3% DMSO (Sigma) for the rest of the culture period, as DMSO has been shown to augment susceptibility of cells to HBV infection^16^. During the infection period, the medium was changed every four days. An aliquot of the supernatant was reserved for HBV gene expression analysis prior to changing of the culture medium at the indicated time points.

### Quantification of extracellular HBV DNA, HBe and HBs Antigen

DNA was extracted from 100 μL of supernatant from the HBV-infected NTCP-HepG2 C4 or T23 cells using the SMITEST EX-R&D Nucleic Acid Extraction Kit (Medical & Biological Laboratories Co, Ltd, Nagoya, Japan) and dissolved in 20 μL of H_2_O. A 1 μL volume of the DNA solution was amplified, and HBV DNA copy numbers were determined by quantitative real-time PCR (qPCR), as we reported previously^10^, using the primers used for detection of total HBV RNA described below. HBs and HBe antigen levels were assayed by Chemiluminescence enzyme immunoassay (CLEIA) using the HISCL HBsAg Assay Kit and the HISCL HBeAg Assay Kit, respectively. Quantification was achieved by the Automated Immunoassay System HISCL-5000 (All from Sysmex Corporation, Kobe, Japan) according to the manufacturer’s instructions.

### Antibodies

The following antibodies were used in the study: rabbit anti-TARDBP (MBL, Life Science), mouse anti-HBV core antibody HB91 (Advanced Life Science Institute Inc., Saitama, Japan), rabbit anti-NTCP/SLC10A1, mouse anti-SIRT1, rabbit anti-Hsp90, rabbit anti-NPM1, rabbit anti-hnRNPC1 + C2, and rabbit anti-hnRNPK (Abcam), goat anti-GAPDH, mouse anti-β-actin, and mouse anti-FLAG M2 (Sigma-Aldrich), rabbit anti-SFPQ, rabbit anti-PARP1, rabbit anti-PTBP1, and mouse anti-PUF60 (GeneTex). The above antibodies were used for western blotting experiments, whereas anti-TARDBP and anti-FLAG M2 were also used for the immunofluorescence assay. For immunoprecipitation of the core-associated HBV genomes, the mouse anti-HBV core monoclonal antibody 2A21 (Institute of Immunology, Tokyo, Japan) was used while the FLAG M2 antibody was used to precipitate FLAG-tagged TARDBP.

### Immunoblotting

Cultured cells were lysed with RIPA buffer consisting of 50 mM Tris-HCl (pH 7.4), 150 mM NaCl, 1% (v/v) NP40, 0.5% sodium deoxycholate, 0.1% SDS and protease inhibitor cocktail (Nacalai Tesque, Kyoto, Japan). The lysates were incubated for 30 min at 4 °C and centrifuged at 14,000 × g for 15 min at 4 °C. Supernatants were reserved and protein concentration determined by the Bradford Protein Assay (Bio-Rad, Japan). A total of 30 μg of the proteins were boiled at 95 °C for 5 min in the SDS sample buffer (Bio-Rad, Japan) and separated by SDS-PAGE. This was followed by a transfer to nitrocellulose membranes, blocking with phosphate-buffered saline (PBS) containing 0.05% Tween 20 (PBST) and 5% skim milk, and incubation with primary antibodies at 4 °C overnight. After three washes with PBST, the membranes were incubated with HRP-conjugated secondary antibodies at room temperature for 1 hour. The membranes were then washed three times with PBST and developed using the ECL western blot reagents (GE Healthcare, Japan). Detected proteins were visualized and analyzed by the ChemiDoc™ XRS+ System (Bio-Rad).

### Detection of intracellular HBV DNA

Core-associated HBV DNA was extracted from the cultured cells and subjected to qPCR and Southern blotting as we previously described^10^. Briefly, the cells were lysed with 250 μL of lysis buffer consisting of 10 mM Tris (pH 7.4), 140 mM NaCl, 0.5% (v/v) NP40 and protease inhibitor cocktail, followed by centrifugation for 2 minutes at 15,000 × g at 4 °C. The lysates were then immunoprecipated by incubation with the mouse anti-HBV core monoclonal antibody followed by extraction and purification of DNA from the precipitates. Quantitative analysis of the core-associated HBV genomes in the DNA was performed with SYBR Green (Life Technologies Japan) using the 7300 Real-Time PCR System (Life Technologies Japan). The primers used were those for total HBV RNA described below. For Southern blot analysis, the DNA equivalent of 10^7^ copies was electrophoresed in a 1% agarose gel and transferred onto a nylon membrane. The membrane was incubated with a full-length HBV DNA probe synthesized with the PCR digoxigein probe synthesis kit. Detection was performed by the DIG Nucleic Acid Detection kit and CSP-Star, ready-to-use (All three by Roche Diagnostics Japan, Tokyo) and visualized by the ChemiDoc XRS+ System (Bio-Rad).

### Immunoprecipitation

The HBV producing cell line T23 was transfected with 5 μg FLAG-TARDBP for 48 hours. The cells were lysed in ice cold Pierce IP lysis buffer consisting of 25 mM Tris/HCl pH 7.4, 150 mM NaCl, 1% NP-40, 1 mM EDTA, 5% glycerol (Thermo Scientific) supplemented with EDTA-free protease inhibitor cocktail (Nacalai), and incubated on ice for 5 minutes with periodic mixing. The lysates were transferred to a micro-centrifuge tube and centrifuged at 13k × g for 10 minutes to pellet the cell debris at 4 °C. The supernatant was immunoprecipitated overnight with 5 μg anti-FLAG M2 antibody (Sigma) in the presence of protein A Sepharose beads (GE Healthcare). As a control, half of the lysate was immunoprecipitated with normal mouse IgG (MBL, Life science). The beads were washed four times in ice cold lysis buffer, treated with SDS sample buffer before being subjected to SDS-PAGE electrophoresis to detect proteins.

### Immunocytochemistry

For immunostaining, NTCP-HepG2 or T23 cultured cells grown on glass slides were fixed with formalin for 10 minutes and permeabilized with 0.25% Triton X-100 (Merck, Darmstadt, Germany) in 10 mM PBS (pH 7.5) for 10 minutes at room temperature. After incubation in PBS containing 10% BSA for 30 minutes, cells were incubated with the primary antibodies, mouse anti-FLAG or rabbit anti-TARDBP diluted in PBS containing 10% BSA for 1 hour at 37 °C. The cells washed twice with PBST, then incubated with the secondary antibodies Alexa 568-conjugated antibody against mouse IgG and Alexa 488-conjugated antibody against rabbit IgG (Life Technologies Japan, Tokyo, Japan), respectively for 1 hour at 37 °C. The cells were shielded from light as from the secondary antibody incubation step. The unbound antibodies were washed off with PBST. Prior to detection, the nuclei were stained with 6-diamidino-2-phenylindole (DAPI) (Vector laboratories, Burlingame, CA). The stained cells were then examined with the Fluoview FV10i microscope (Olympus, Tokyo, Japan).

### Expression and purification of recombinant TARDBP

GST-tagged recombinant TARDBP was expressed in BL21 (DE3) ECOS^TM^-competent *E. coli* cells (Nippon Gene, Tokyo, Japan) from the pGEX-TARDBP plasmid. Induction of protein expression was carried out with 0.5 mm isopropyl β-d-thiogalactopyranoside (IPTG) and cells were grown at 37 °C for 3 hours. Cell pellets were resuspended in a GST binding buffer consisting of 150 mM NaCl, 25 mM Tris (pH 8.0), 0.05% (v/v) NP40, 1 mM EDTA (+protease inhibitors) and 0.25 mg/ml of lysozyme. Lysates were centrifuged for 20 min at 20,000 × g, 4 °C following sonication on ice. The GST protein was precipitated using Glutathione Sepharose 4B according to the manufacturer’s instructions (GE Healthcare Life Sciences). Elution of protein from the beads was achieved using a GST elution buffer containing 10 mM reduced glutathione (Sigma), pH 8.0 dissolved in 50 mM Tris-HCl.

### Real-time PCR

For mRNA quantification, RNA was extracted from cultured cells by the QuickGene RNA cultured cell kit S (RC-S) and a 50 μL volume of RNA was obtained using QuickGene-810 equipment, according to the manufacturer’s instructions (KURABO, Osaka, Japan). In a 20 μL reaction, total RNA (0.5 μg) was converted to cDNA using the reverse transcription Takara PrimeScript RT Master Mix (TaKaRa Bio, Shiga, Japan) and diluted using qPCR Takara EASY Dilution. The Brilliant III SYBR® MM with ROX PCR master mix (Agilent Technologies, CA) was used to prepare the diluted cDNA samples for quantification by the Agilent Mx3000P QPCR system. The following primer sets were used for mRNA quantification: TARDBP, 5′-CCCCAGATATTGCCAATATC-3′ and 5′-AAGTTTCCAATATGCTCAATTAAG-3′; Precore, 5′-GGTCTGTTCACCAGCACCAT-3′ and 5′-GGAAAGAAGTCAGAAGGCAA-3′; Core, 5′-CCGGAAAGCTTGAGCTCTTCTT-3′ and 5′-CACAGAATAGCTTGCCTGAGTG-3′; APOA2 5′-CAACTGTGCTACTCCTCACCAT-3′ and 5′-TGGAAGTACTGAGAAACCAGG C-3′; Total HBV RNA, 5′-GAGTGCTGTATGGTGAGGTG-3′ and 5′-TTTGGGGCATGGACATTGAC-3′. Quantification of HBV mRNAs (precore and pregenomic) was performed as we described recently^62^. Gene expression was normalized to that of GAPDH. Relative mRNA levels represent the level of the gene divided by the level of GAPDH mRNA.

### Generation of TARDBP-knockout NTCP-HepG2 cells

NTCP-HepG2 cells were subjected to a conditional knock-out of the TARDBP gene for functional analysis. We employed the CRISPR/Cas9-mediated destabilization domain (DD)-tag knock-in strategy, which allows rapid conditional knock-down of protein function^59^. To generate stable knock-in cell lines, NTCP-HepG2 cells were inoculated at 8 × 10^5^ cells/six-well plate with no-antibiotics medium, and after 24 hours, the cells were co-transfected with each 0.5 μg of TARDBP_guide 1/pX330, TARDBP_guide 3/pX330, TARDBP puro-DD knock-in plasmid and TARDBP bla-DD knock-in plasmid (Fig. S1), using Lipofectamine 3000 as per the manufacturer’s instructions (Life Technologies). At 24 hours post-transfection, 100 nM Shield-1 (Clontech) was added to the culture medium, and cells were subsequently always maintained in Shield-1. Beyond 24 hours, the medium was replaced with that containing 1.5 μg/mL puromycin, 1.5 μg/mL blasticidin and antibiotics. At ≥30 days post-transfection, colonies were isolated and expanded. Gene knockout was confirmed by western blotting. In the destabilizing domain (DD) knock-in strategy, the protein is fused to the DD tag such that, in the absence of the DD specific ligand (Shield-1), the protein is degraded by the proteasome. However, when Shield1 is added to the culture medium, the fusion protein should accumulate within 24 hours. Unfortunately, our attempts to add Shield-1 led to failure to detect any TARDBP protein (Fig. S2A). Henceforth, for the rescue experiments, TARDBP was expressed in TARDBP KO cells using a transiently transfected plasmid.

### Chromatin immunoprecipitation (ChIP) assays

For HBV promoter analysis, the ChIP assay was performed on genomic DNA samples from the HBV-producing cell line T23 using the ChIP-IT Express Enzymatic kit according to the manufacturer’s protocol (Active Motif, Tokyo, Japan). Briefly, T23 cells were grown to confluency in 10 cm dishes and cross-linked with formaldehyde. The reaction was stopped by glycine followed by cell lysis with the cold lysis buffer provided in the kit. Chromatin was sheared from the lysates with the enzymatic shearing cocktail and immunoprecipitated overnight with the magnetic beads in the presence of antibodies. The antibodies used for immunoprecipitation were the rabbit anti-TARDBP and the rabbit control IgG (MBL, Life science). Chromatin was eluted from the beads and crosslinking reversed with 5 M NaCl. The samples were treated with proteinase K and subjected to qPCR analysis by the CFX96^TM^ Real-Time PCR Detection system (Bio-Rad, Tokyo, Japan). The region corresponding to each of the four HBV promoters (X, Core, SP1 and SP2) was amplified using the following sets of primers, respectively:

X, 5′-CCGCTCGAGTGGCTCCTCTGCCGATCCATA-3′ (forward) 5′-CCCAAGCTTGGAAAGGAGGTGTATTTCCGA-3′ (reverse)

Core, 5′-CCGCTCGAGAACCACCGTGAACGCCCGCCA-3′ (forward) 5′-CCCAAGCTTACATGAGATGATTAGGCAGAG-3′ (reverse)

SP1, 5′-CCGCTCGAGAGATCTCAATCTCGGGAATCT-3′ (forward) 5′-CCCAAGCTTCCACTGCATGGCCTGAGGATG-3′ (reverse)

SP2, 5′-CCGCTCGAGGATCAGGGTTCACCCCACCAC-3′ (forward) 5′-CCCAAGCTTGAGATGGGAGTAGGCTGTCTC-3′ (reverse)

Each of the reagents used for chromatin extraction and purification were provided in the kit.

For HBV cccDNA ChIP assay, the nuclear extract from a chimeric mouse tissue was immunoprecipitated with control, TARDBP, or HBc antibodies using the Dynabeads Co-Immunoprecipitation Kit and Dynabeads Antibody Coupling Kit (Invitrogen) as per the manufacturer’s instructions. The DNA bound to the beads was extracted by the Smittest EX-R & D kit (MBL, Life Science) followed by rcDNA/nicked dsDNA digestion using T5 Exonuclease (NEB: M0363S). The DNA was purified by the MinElute Reaction Cleanup Kit (QIAGEN: 28204) followed by qPCR analysis (BIO-RAD CFX 96 Real Time System). The primers and probe used for detection of cccDNA were as follows:

Forward primer 1521–1545 GGGGCGCACCTCTCTTTACGCGGTC,

Reverse primer 1862–1886 CAAGGCACAGCTTGGAGGCTTGAAC,

FAM Probe 1685–1704 AACGACCGACCTTGAGGCAT.

### Dual luciferase assay

The luciferase reporter plasmid driven by the HBV core promoter, pGL3-Cp was co-transfected with the pcDNA3.1-FLAG (empty vector), the pcDNA3.1-FLAG/TARDBP or the pcDNA3.1-FLAG/TARDBP RRM mutant plasmids into NTCP-HepG2 cells. The pRL-TK plasmid (Promega, USA) was transfected with the reporter plasmid in all wells to normalize the transfection efficiency. At 48 hours after transfections, the cells were lysed in the Passive Lysis Buffer and assayed using the Dual-Luciferase Reporter assay system (both by Promega, USA) according to the manufacturer’s instructions. Firefly and renilla luciferase activity was determined using the Mithras LB 940 Multimode Microplate Reader (Berthold Technologies).

### Electrophoretic mobility shift assay (EMSA)

12 overlapping (6 sense and 6 anti-sense) probes of about 46 bp in size were prepared from the HBV core promoter spanning the region nt1613–1849 of the HBV genome. The probes were labelled with biotin using the Pierce Biotin 3′ End DNA Labeling Kit according to the manufacturer’s instructions. The recombinant GST-TARDBP protein used in EMSA was produced as described above. The EMSA reaction was carried out using the Pierce LightShift™ Chemiluminescent EMSA Kit. A 20 μl EMSA reaction was performed as per the manufacturer’s protocol with 2 μg (or as indicated) of TARDBP protein, 1% BSA, 100 fmol of labeled probe, and ×200 (or as indicated) amount of unlabeled oligonucleotide competitor. For the super-shift assays, the EMSA reaction was performed in the presence of 2 μg of the rabbit anti-TARDBP antibody. After 30 minutes incubation at room temperature, the reaction products were separated by electrophoresis in a 5% polyacrylamide gel containing 0.5 × TBE. The samples were then transferred to a positively charged Biodyne™ B Nylon Membrane and detected by the Pierce Chemiluminescent Nucleic Acid Detection Module Kit as per the manufacturer’s protocol. The three kits used for EMSA experiments were purchased from Thermoscientific, Japan.

### Mass spectrometry analysis

The HBV replicating cell line T23 was cultured in 10 cm dishes to confluency. Growth medium was removed and cells were rinsed with DPBS and centrifuged at 3000 rpm for 5 min at 4 °C. The supernatant was discarded and 2 ml (5 × PCV) of lysis buffer consisting of 100 mM HEPES/pH7.9, 15 mM MgCl_2_, 100 mM KCl, pure water, 0.1 M DTT, 100 × Protease Inhibitor was added to 400 μl PCV (packed cell volume). The pellet was gently re-suspended and placed on ice for 30 min. 10% NP 40 was added to a final concentration of 0.1%. Cells were disrupted using 7 strokes with a narrow-gauge (No. 27) hypodermic needle, and lysis to 80–90% was confirmed via microscopic examination. Lysed cells were centrifuged again at 2500 rpm for 5 min at 4 °C, and the supernatant (representing the cytoplasmic fraction) was discarded. The nuclear pellet was gently re-suspended with 500 μL of wash buffer (10 ml DPBS, 100 × Protease Inhibitor) and centrifuged again at 2500 rpm for 5 min at 4 °C. The supernatant was discarded and the pellet was rewashed until the background became clear under the microscope. The nuclear pellet was re-suspended in 270 μl (2/3 × PCV) of high salt extraction buffer consisting of 5xIP buffer [Dynabeads Co-Immunoprecipitation Kit, Novex], 5 M NaCl, 0.1 M DTT [CelLytic NuCLEAR Extraction Kit, Sigma], 100xProtease Inhibitor Cocktail [Nakalai tesque, Japan] and homogenized using a narrow-gauge hypodermic needle. 1/5 volume of RIPA Buffer was added, followed by homogenization with a narrow-gauge hypodermic needle and rotation for 1 hr at 4 °C. After centrifugation at 13,000 rpm for 15 min at 4 °C, the supernatant was transferred to a chilled tube. Co-immunoprecipitation of 2.2 mg of extracted nuclear proteins with anti-TARDBP antibody was performed using the Dynabeads Co-Immunoprecipitation Kit (Novex). The sample was prepared for mass spectrometry using the Pierce Mass Spec Sample Prep Kit for Cultured Cells (Thermo Fisher Scientific). LC-MS/MS was performed using the UltiMate 3000 RSLC Nano System (Thermo Fisher Scientific) with the LTQ Orbitrap XL (Thermo Fisher Scientific). The NIKKYO NANO HPLC CAPILLARY COLUMN (3 μm C18, 75 μm I.D. × 120 mm) (Nikkyo Technos) was used for the separation column, and C18 PepMap100 (ThermoFisher Scientific) (300 μm I.D × 5 mm) was used for the trap column. The flow rate was 200 nL/min (mobile phase: A = 0.1% formic acid-Water, B = 0.1% formic acid-Acetonitrile). The precursor scan was performed using the FTMS analyzer (Orbitrap) with a scan range of 300–1,500 m/z and a resolution of 60,000. The data-dependent MS/MS scan (Top 10) was performed using an Ion Trap analyzer with CID activation type and a normalization collision energy of 35% and an isolation width of 2.0 m/z. Analysis was performed using Proteome Discoverer 1.4 software (ThermoFisher Scientific) using the MASCOT search engine, and Swiss-Prot and an HBV protein database (LHBs/Poly/HBc/HBx) were used for annotation. Up to two missed cleavages were allowed, with carbidomethyl (C) as a fixed modification and oxidation (M) as a variable modification. Mass tolerance was set as follows: precursor ion: 10 ppm and fragment ion: 0.8 Da. FDR was set at 5%.

### RNA Immunoprecipitation

RNA precipitation was performed using the RNA Immunoprecipitation (RIP) Assay Kit (MBL) according to the manufacturer’s instruction. Briefly, the HBV producing T23 cell line was transfected with a plasmid expressing FLAG-tagged TARDBP or the empty vector as a control. After 48 hours, cell lysates were harvested and precipitated overnight with an anti-FLAG antibody. Messenger RNP complexes bound to protein were isolated from the precipitate and purified, followed by reverse transcription. The cDNA was then utilized for qPCR analysis using primers specific for TARDBP and APOA2 (positive controls), GAPDH (negative control), and total HBV RNA, as described above.

### Statistical analysis

All experiments were repeated at least three times. The bars in graphs represent means ± SD. Significance was determined using a two-tailed unpaired Student’s t-test. Values of P < 0.05 were considered to be statistically significant.

## Supplementary information

Supplementary Information
