# Publisher Correction: Regulation of the Hepatitis B virus replication and gene expression by the multi-functional protein TARDBP

**DOI:** 10.1038/s41598-020-58888-6

**Published:** 2020-02-05

**Authors:** Grace Naswa Makokha, Hiromi Abe-Chayama, Sajeda Chowdhury, C. Nelson Hayes, Masataka Tsuge, Tadahiko Yoshima, Yuji Ishida, Yizhou Zhang, Takuro Uchida, Chise Tateno, Rie Akiyama, Kazuaki Chayama

**Affiliations:** 1 Department of Gastroenterology and Metabolism, Institute of Biomedical and Health Science, Hiroshima, Japan; 2 Liver Research Project Center, Hiroshima, Japan; 3 Natural Science Center for Basic Research and Development, Hiroshima, Japan; 4 Center for Medical Specialist Graduate Education and Research, Hiroshima, Japan; 5 Laboratory for Digestive Diseases, RIKEN Center for Integrative Medical Sciences Hiroshima University, 1-2-3 Kasumi, Minami-ku, Hiroshima-shi, 734-8551 Japan; 6 PhoenixBio Co., Ltd., 3-4-1 Kagamiyama, Higashihiroshima, 739-0046 Japan

Correction to: *Scientific Reports* 10.1038/s41598-019-44934-5, published online 11 June 2019

In the original version of this Article, Figure 5 was published with an incorrect version of panel 5b. This has now been corrected in the HTML and PDF versions of the Article.

