# Supplementary material for: Regulation of the Hepatitis B virus replication and gene expression by the multi-functional protein TARDBP
Source: Sci Rep. 2019 Jun 11;9:8462. doi: 10.1038/s41598-019-44934-5 (PMC6560085; doi:10.1038/s41598-019-44934-5)
Supplement: Supplementary file 1 — Supplementary Information [file 41598_2019_44934_MOESM1_ESM.pdf]

**Regulation of the Hepatitis B virus replication and gene expression by the multi-functional protein TARDBP**

Grace Naswa Makokha, Hiromi Abe-Chayama, Sajeda Chowdhury, C. Nelson Hayes, Masataka Tsuge, Tadahiko Yoshima, Yuji Ishida, Yizhou Zhang, Takuro Uchida, Chise Tateno, Rie Akiyama, Kazuaki Chayama.

## SUPPLEMENTAL FIGURE LEGENDS

**Figure S1. TARDBP knock-in donor plasmids**. The sequences of left and right homology arm of TARDBP (A), TARDBP puro-DD knock-in cassette (B), and TARDBP bla-DD knock-in cassette (C).

**Figure S2. Over-expression of TARDBP in NTCP-HepG2/TARDBP knock-out (Tar KO) cells**. (A) Western blotting was performed on NTCP parent cells and NTCP Tar KO cells treated with or without Shield1 (500nM and 1000nM). (B) The pcDNA-FLAG TARDBP plasmid was transfected into NTCP-Tar KO cells for exogenous expression. The cells were harvested at days 0, 3, 6, and 9 after transfection for western blotting analysis. The amount of protein expressed at each time point was detected using anti-TARDBP antibody. GAPDH was used as a loading control.

**Figure S3. The EMSA probes from the HBV core promoter region**. The sequences of the 12 probes (6-sense and 6-antisense) constructed from the entire core promoter region of nt1613-1849 of the HBV genome. The probes were labeled as sense (S) probes 1-6 and anti-sense (AS) probes 1-6 in a consecutive manner, starting from the C-terminal region backwards to the N-terminus.

**Figure S4. TARDBP binds to a sequence within the HBV core promoter** (A) The 12 probes were subjected to EMSA with 2µg of a GST tagged TARDBP as follows: S probe 1 alone (lane 1), S probes 1-6 with TARDBP protein (lanes 2-7), AS probes 1-6 with protein (lanes 8-13), and a re-probe of S probe 3 (lanes 14-15). (B) The two probes that showed possible binding to TARDBP in (A) based on the presence of a shifted band (S probe 3 and AS probe 1) were re-subjected to EMSA in the presence or absence of protein together with the corresponding unlabeled probe as a specific competitor and detected by short exposure (left panel) and a long exposure (right panel). (C) Four short fragments of about 24-28 bp labeled s1-s4 were constructed from AS probe 1 (WT). The nucleotide sequence and position of each fragment in the HBV genome is shown. (D) Each of the probes in C was subjected to EMSA in the presence or absence

of the TARDBP protein as follows: WT alone (lane 1), WT plus protein (lane 2), s1 alone (lane 3), s1 plus protein (lane 4), s2 alone (lane 5), s2 plus protein (lane 6), s3 alone (lane 7), s3 plus protein (lane 8), s4 alone (lane 9), and s4 plus protein (lane 10). The location of the free probe, the TARDBP-DNA complex (shifted probe), and the putative secondary structure is indicated by an arrow.

**Figure S5. TARDBP failed to precipitate HBV cccDNA**. Immunoprecipitation was performed on nuclear protein derived from chimeric mice frozen tissue using the anti-rabbit (negative control), anti-TARDBP and anti-HBc (positive control) antibodies. The amount of cccDNA captured by the beads (antibody) was quantified. Prior to quantification, the DNA was pretreated with T5 exonuclease to digest all DNA other than cccDNA. The values are presented as a ratio (%) of DNA quantified in the pellet versus that detected in the supernatant of the immunoprecipitated samples.

**Figure S6. Prediction of TARDBP RNA-binding domains in the HBV transcriptome.** (A) Because TARDBP is well known to bind preferentially to UG-rich regions of RNA, we tried to identify potential TARDBP HBV RNA binding sites by examining the HBV genome for conserved TG-repeats. To identify potential TARDBP HBV RNA binding sites, HBV genotype C genome alignments were downloaded from HBVdb (https://hbvdb.ibcp.fr/HBVdb/; accessed on 2016/12/20). In this Jalview genome alignment, thymine was colored in green and guanine was colored in orange, indicating that the HBV genome contains a number of clusters of T or G nucleotides. However, when we performed a regular expression search for (TG)+ repeats, we found few conserved TG stretches with more than two repeats, e.g., the short pattern outlined in red, suggesting that it would be difficult to determine the TARDBP RNA binding site using only a regular expression search. (B) However, published TARDBP RNA binding site motifs suggest that while most patterns contain a core pattern of about 6 TG repeats, the nucleotide profile at each position is somewhat flexible. Therefore, we downloaded the 11 experimentally determined TARDBP RNA-binding motifs from the RBPDB database of RNA-binding protein specificities (http://rbpdb.ccbr.utoronto.ca/index.php; accessed on 2018/11/01) and performed a fuzzy search (http://www.bioinformatics.org/sms2/fuzzy_search_dna.html; reference sequence = KR819180.1, match = 5, mismatch = -1, and gap = -5) to find the highest scoring matches for each pattern without over-penalizing for mismatches. (C) Fuzzy matches were exported in BED format and uploaded to the NCBI Graphics panel for HBV reference sequence KR819180.1. We observed a number of matches within the X transcript overlapping the core promoter/enhancer II region (yellow) and found other clusters within the S and core transcripts, as well as a match is downstream of the polyadenylation site (blue). These results suggest that TARDBP might

interact with HBV transcripts within the 3’ non-coding regions shared by all transcripts and might interact with HBV RNA in the same region as the DNA binding site in the core promoter/enhancer II region.

**Figure S7-S14. Full length blots used to generate main figures.**

## SUPPLEMENTAL TABLES AND FIGURES

**Table S1. PCR primer sequences.**

| PCR primer | |
|---|---|
| Name | Sequence (5' to 3') |
| LHA1F | GTTCTTTACACCTGGTTCACTGCCC |
| RHA2R | GAGGCAGAGAGAAGGATAAGACCAG |
| LHA2F | GAGGAACAGAGGGAAACTTTTCTCCTG |
| RHA3R | CAGATCGACTACTAACAGCACACTACTTC |
| DDF | GAAGATGGAAAGAAAGTCGATTCCTC |
| DDR | GGCACCATAGGCATAATCTGGAG |
| puroF2 | ACCACCAGGGCAAGGGTCTG |
| puroR1 | AAGCCGAGCCGCTCGTAGAAG |
| blaF1 | GCTGGCAACCTGACTTGTATCGTC |
| blaR1 | CACTGTCCTTCACTATGGCTTTG |

**Table S2. PCR primer sets and product sizes.**

| PCR analysis of knock-in clones Genomic DNA | | |
|---|---|---|
| Forward primer | Reverse primer | product size (bp) |
| LHA2F | RHA3R | 2389 + 2188 |
| LHA2F | DDR | 1444 + 1243 |
| LHA2F | puroR1 | 1053 |
| LHA2F | blaR1 | 860 |
| DDF | RHA3R | 1106 |
| puroF2 | RHA3R | 1466 |
| blaF1 | RHA3R | 1453 |

**Table S3. List of candidate proteins co-immunoprecipitated with TARDBP and identified by mass spectrometry analysis.**

| Accession | Description | ΣCoverage | Σ# Proteins | Σ# Unique Peptides | MW [kDa] |
|---|---|---|---|---|---|
| P02545 | Prelamin-A/C OS=Homo sapiens GN=LMNA PE=1 SV=1 - [LMNA_HUMAN] | 63.86 | 4 | 43 | 74.1 |
| P08727 | Keratin, type I cytoskeletal 19 OS=Homo sapiens GN=KRT19 PE=1 SV=4 - [K1C19_HUMAN] | 63.50 | 14 | 18 | 44.1 |
| P05787 | Keratin, type II cytoskeletal 8 OS=Homo sapiens GN=KRT8 PE=1 SV=7 - [K2C8_HUMAN] | 61.70 | 14 | 30 | 53.7 |
| P52272 | Heterogeneous nuclear ribonucleoprotein M OS=Homo sapiens GN=HNRNPM PE=1 SV=3 - [HNRPM_H | 57.53 | 1 | 35 | 77.5 |
| Pierce | Digestion Indicator | 57.33 | 2 | 11 | 25.7 |
| P60709 | Actin, cytoplasmic 1 OS=Homo sapiens GN=ACTB PE=1 SV=1 - [ACTB_HUMAN] | 55.47 | 9 | 6 | 41.7 |
| Q9Y3B4 | Splicing factor 3B subunit 6 OS=Homo sapiens GN=SF3B6 PE=1 SV=1 - [SF3B6_HUMAN] | 53.60 | 3 | 4 | 14.6 |
| P22626 | Heterogeneous nuclear ribonucleoproteins A2/B1 OS=Homo sapiens GN=HNRNPA2B1 PE=1 SV=2 - [F | 52.97 | 1 | 15 | 37.4 |
| Q9UJV9 | Probable ATP-dependent RNA helicase DDX41 OS=Homo sapiens GN=DDX41 PE=1 SV=2 - [DDX41_H | 51.45 | 1 | 26 | 69.8 |
| P04264 | Keratin, type II cytoskeletal 1 OS=Homo sapiens GN=KRT1 PE=1 SV=6 - [K2C1_HUMAN] | 49.38 | 3 | 25 | 66.0 |
| P20700 | Lamin-B1 OS=Homo sapiens GN=LMNB1 PE=1 SV=2 - [LMNB1_HUMAN] | 47.95 | 1 | 25 | 66.4 |
| Q15233 | Non-POU domain-containing octamer-binding protein OS=Homo sapiens GN=NONO PE=1 SV=4 - [NC | 45.65 | 1 | 18 | 54.2 |
| P17844 | Probable ATP-dependent RNA helicase DDX5 OS=Homo sapiens GN=DDX5 PE=1 SV=1 - [DDX5_HUM | 45.28 | 1 | 17 | 69.1 |
| P35527 | Keratin, type I cytoskeletal 9 OS=Homo sapiens GN=KRT9 PE=1 SV=3 - [K1C9_HUMAN] | 44.14 | 1 | 19 | 62.0 |
| P05783 | Keratin, type I cytoskeletal 18 OS=Homo sapiens GN=KRT18 PE=1 SV=2 - [K1C18_HUMAN] | 43.26 | 11 | 21 | 48.0 |
| P09651 | Heterogeneous nuclear ribonucleoprotein A1 OS=Homo sapiens GN=HNRNPA1 PE=1 SV=5 - [ROA1_H | 42.74 | 2 | 12 | 38.7 |
| P07910 | Heterogeneous nuclear ribonucleoproteins C1/C2 OS=Homo sapiens GN=HNRNPC PE=1 SV=4 - [HNR | 41.18 | 4 | 16 | 33.6 |
| P62805 | Histone H4 OS=Homo sapiens GN=HIST1H4A PE=1 SV=2 - [H4_HUMAN] | 40.78 | 1 | 4 | 11.4 |
| P22087 | rRNA 2'-O-methyltransferase fibrillarin OS=Homo sapiens GN=FBL PE=1 SV=2 - [FBRL_HUMAN] | 40.19 | 2 | 9 | 33.8 |
| P55769 | NHP2-like protein 1 OS=Homo sapiens GN=SNU13 PE=1 SV=3 - [NH2L1_HUMAN] | 39.84 | 1 | 3 | 14.2 |
| P06748 | Nucleophosmin OS=Homo sapiens GN=NPM1 PE=1 SV=2 - [NPM_HUMAN] | 38.44 | 1 | 10 | 32.6 |
| Q01082 | Spectrin beta chain, non-erythrocytic 1 OS=Homo sapiens GN=SPTBN1 PE=1 SV=2 - [SPTB2_HUMAN | 37.90 | 6 | 70 | 274.4 |
| P31943 | Heterogeneous nuclear ribonucleoprotein H OS=Homo sapiens GN=HNRNPH1 PE=1 SV=4 - [HNRH1_ | 37.86 | 1 | 4 | 49.2 |
| P84103 | Serine/arginine-rich splicing factor 3 OS=Homo sapiens GN=SRSF3 PE=1 SV=1 - [SRSF3_HUMAN] | 37.80 | 1 | 2 | 19.3 |
| Q03252 | Lamin-B2 OS=Homo sapiens GN=LMNB2 PE=1 SV=4 - [LMNB2_HUMAN] | 37.42 | 1 | 17 | 69.9 |
| P62316 | Small nuclear ribonucleoprotein Sm D2 OS=Homo sapiens GN=SNRPD2 PE=1 SV=1 - [SMD2_HUMAN] | 37.29 | 1 | 4 | 13.5 |
| P0CG48 | Polyubiquitin-C OS=Homo sapiens GN=UBC PE=1 SV=3 - [UBC_HUMAN] | 36.79 | 4 | 2 | 77.0 |
| Q07955 | Serine/arginine-rich splicing factor 1 OS=Homo sapiens GN=SRSF1 PE=1 SV=2 - [SRSF1_HUMAN] | 36.69 | 1 | 9 | 27.7 |
| P16403 | Histone H1.2 OS=Homo sapiens GN=HIST1H1C PE=1 SV=2 - [H12_HUMAN] | 36.15 | 4 | 9 | 21.4 |
| P60660 | Myosin light polypeptide 6 OS=Homo sapiens GN=MYL6 PE=1 SV=2 - [MYL6_HUMAN] | 35.76 | 2 | 5 | 16.9 |
| Q13242 | Serine/arginine-rich splicing factor 9 OS=Homo sapiens GN=SRSF9 PE=1 SV=1 - [SRSF9_HUMAN] | 35.75 | 1 | 3 | 25.5 |
| O60814 | Histone H2B type 1-K OS=Homo sapiens GN=HIST1H2BK PE=1 SV=3 - [H2B1K_HUMAN] | 34.92 | 17 | 5 | 13.9 |
| P25705 | ATP synthase subunit alpha, mitochondrial OS=Homo sapiens GN=ATP5A1 PE=1 SV=1 - [ATPA_HUM | 34.90 | 1 | 14 | 59.7 |
| P68032 | Actin, alpha cardiac muscle 1 OS=Homo sapiens GN=ACTC1 PE=1 SV=1 - [ACTC_HUMAN] | 34.75 | 6 | 1 | 42.0 |
| P84090 | Enhancer of rudimentary homolog OS=Homo sapiens GN=ERH PE=1 SV=1 - [ERH_HUMAN] | 34.62 | 1 | 3 | 12.3 |
| Q9NWB6 | Arginine and glutamate-rich protein 1 OS=Homo sapiens GN=ARGLU1 PE=1 SV=1 - [ARGL1_HUMAN] | 34.43 | 1 | 9 | 33.2 |
| Q13148 | TAR DNA-binding protein 43 OS=Homo sapiens GN=TARDBP PE=1 SV=1 - [TADBP_HUMAN] | 34.30 | 2 | 14 | 44.7 |
| Q13813 | Spectrin alpha chain, non-erythrocytic 1 OS=Homo sapiens GN=SPTAN1 PE=1 SV=3 - [SPTN1_HUMAI | 34.14 | 1 | 59 | 284.4 |
| Q92841 | Probable ATP-dependent RNA helicase DDX17 OS=Homo sapiens GN=DDX17 PE=1 SV=2 - [DDX17_H | 34.02 | 1 | 14 | 80.2 |

| | | | | | |
|---|---|---|---|---|---|
| P55795 | Heterogeneous nuclear ribonucleoprotein H2 OS=Homo sapiens GN=HNRNPH2 PE=1 SV=1 - [HNRH2 | 33.63 | 1 | 4 | 49.2 |
| P62906 | 60S ribosomal protein L10a OS=Homo sapiens GN=RPL10A PE=1 SV=2 - [RL10A_HUMAN] | 33.18 | 1 | 3 | 24.8 |
| Q86V81 | THO complex subunit 4 OS=Homo sapiens GN=ALYREF PE=1 SV=3 - [THOC4_HUMAN] | 33.07 | 1 | 6 | 26.9 |
| P61978 | Heterogeneous nuclear ribonucleoprotein K OS=Homo sapiens GN=HNRNPK PE=1 SV=1 - [HNRPK_HU | 33.05 | 1 | 14 | 50.9 |
| P42167 | Lamina-associated polypeptide 2, isoforms beta/gamma OS=Homo sapiens GN=TMPO PE=1 SV=2 - [I | 33.04 | 2 | 9 | 50.6 |
| P09874 | Poly [ADP-ribose] polymerase 1 OS=Homo sapiens GN=PARP1 PE=1 SV=4 - [PARP1_HUMAN] | 33.04 | 1 | 20 | 113.0 |
| P35579 | Myosin-9 OS=Homo sapiens GN=MYH9 PE=1 SV=4 - [MYH9_HUMAN] | 32.91 | 2 | 42 | 226.4 |
| Q16629 | Serine/arginine-rich splicing factor 7 OS=Homo sapiens GN=SRSF7 PE=1 SV=1 - [SRSF7_HUMAN] | 32.35 | 1 | 5 | 27.4 |
| P11142 | Heat shock cognate 71 kDa protein OS=Homo sapiens GN=HSPA8 PE=1 SV=1 - [HSP7C_HUMAN] | 32.20 | 6 | 13 | 70.9 |
| P23246 | Splicing factor, proline- and glutamine-rich OS=Homo sapiens GN=SFPQ PE=1 SV=2 - [SFPQ_HUMAN | 31.97 | 1 | 20 | 76.1 |
| Q13151 | Heterogeneous nuclear ribonucleoprotein A0 OS=Homo sapiens GN=HNRNPA0 PE=1 SV=1 - [ROA0_H | 31.80 | 1 | 4 | 30.8 |
| P62318 | Small nuclear ribonucleoprotein Sm D3 OS=Homo sapiens GN=SNRPD3 PE=1 SV=1 - [SMD3_HUMAN] | 31.75 | 1 | 3 | 13.9 |
| P39019 | 40S ribosomal protein S19 OS=Homo sapiens GN=RPS19 PE=1 SV=2 - [RS19_HUMAN] | 31.72 | 1 | 3 | 16.1 |
| Q9UHX1 | Poly(U)-binding-splicing factor PUF60 OS=Homo sapiens GN=PUF60 PE=1 SV=1 - [PUF60_HUMAN] | 31.66 | 1 | 14 | 59.8 |
| P07305 | Histone H1.0 OS=Homo sapiens GN=H1F0 PE=1 SV=3 - [H10_HUMAN] | 31.44 | 1 | 4 | 20.9 |
| P51991 | Heterogeneous nuclear ribonucleoprotein A3 OS=Homo sapiens GN=HNRNPA3 PE=1 SV=2 - [ROA3_H | 31.22 | 3 | 13 | 39.6 |
| P62701 | 40S ribosomal protein S4, X isoform OS=Homo sapiens GN=RPS4X PE=1 SV=2 - [RS4X_HUMAN] | 31.18 | 2 | 5 | 29.6 |
| O43390 | Heterogeneous nuclear ribonucleoprotein R OS=Homo sapiens GN=HNRNPR PE=1 SV=1 - [HNRPR_HU | 30.81 | 1 | 9 | 70.9 |
| P14866 | Heterogeneous nuclear ribonucleoprotein L OS=Homo sapiens GN=HNRNPL PE=1 SV=2 - [HNRPL_HU | 30.73 | 1 | 12 | 64.1 |
| Q9Y6A4 | Cilia- and flagella-associated protein 20 OS=Homo sapiens GN=CFAP20 PE=1 SV=1 - [CFA20_HUMAN | 30.57 | 1 | 2 | 22.8 |
| Q01130 | Serine/arginine-rich splicing factor 2 OS=Homo sapiens GN=SRSF2 PE=1 SV=4 - [SRSF2_HUMAN] | 30.32 | 2 | 7 | 25.5 |
| Q9NR30 | Nucleolar RNA helicase 2 OS=Homo sapiens GN=DDX21 PE=1 SV=5 - [DDX21_HUMAN] | 30.27 | 2 | 15 | 87.3 |
| P30050 | 60S ribosomal protein L12 OS=Homo sapiens GN=RPL12 PE=1 SV=1 - [RL12_HUMAN] | 29.70 | 1 | 3 | 17.8 |
| P38159 | RNA-binding motif protein, X chromosome OS=Homo sapiens GN=RBMX PE=1 SV=3 - [RBMX_HUMAN | 29.67 | 4 | 9 | 42.3 |
| P11387 | DNA topoisomerase 1 OS=Homo sapiens GN=TOP1 PE=1 SV=2 - [TOP1_HUMAN] | 29.41 | 2 | 14 | 90.7 |
| P62263 | 40S ribosomal protein S14 OS=Homo sapiens GN=RPS14 PE=1 SV=3 - [RS14_HUMAN] | 29.14 | 1 | 3 | 16.3 |
| P13645 | Keratin, type I cytoskeletal 10 OS=Homo sapiens GN=KRT10 PE=1 SV=6 - [K1C10_HUMAN] | 29.11 | 12 | 8 | 58.8 |
| P05141 | ADP/ATP translocase 2 OS=Homo sapiens GN=SLC25A5 PE=1 SV=7 - [ADT2_HUMAN] | 28.86 | 2 | 2 | 32.8 |
| P62820 | Ras-related protein Rab-1A OS=Homo sapiens GN=RAB1A PE=1 SV=3 - [RAB1A_HUMAN] | 28.78 | 24 | 5 | 22.7 |
| O00422 | Histone deacetylase complex subunit SAP18 OS=Homo sapiens GN=SAP18 PE=1 SV=1 - [SAP18_HUM | 28.76 | 1 | 4 | 17.5 |
| P61247 | 40S ribosomal protein S3a OS=Homo sapiens GN=RPS3A PE=1 SV=2 - [RS3A_HUMAN] | 28.03 | 1 | 4 | 29.9 |
| P55081 | Microfibrillar-associated protein 1 OS=Homo sapiens GN=MFAP1 PE=1 SV=2 - [MFAP1_HUMAN] | 27.79 | 1 | 4 | 51.9 |
| Q9UKM9 | RNA-binding protein Raly OS=Homo sapiens GN=RALY PE=1 SV=1 - [RALY_HUMAN] | 27.78 | 1 | 8 | 32.4 |
| P48047 | ATP synthase subunit O, mitochondrial OS=Homo sapiens GN=ATP5O PE=1 SV=1 - [ATPO_HUMAN] | 27.23 | 1 | 4 | 23.3 |
| Q08211 | ATP-dependent RNA helicase A OS=Homo sapiens GN=DHX9 PE=1 SV=4 - [DHX9_HUMAN] | 27.09 | 3 | 27 | 140.9 |
| P62269 | 40S ribosomal protein S18 OS=Homo sapiens GN=RPS18 PE=1 SV=3 - [RS18_HUMAN] | 26.97 | 1 | 3 | 17.7 |
| Q14103 | Heterogeneous nuclear ribonucleoprotein D0 OS=Homo sapiens GN=HNRNPD PE=1 SV=1 - [HNRPD_ | 26.48 | 2 | 5 | 38.4 |
| P11021 | 78 kDa glucose-regulated protein OS=Homo sapiens GN=HSPA5 PE=1 SV=2 - [GRP78_HUMAN] | 26.30 | 1 | 8 | 72.3 |
| Q15459 | Splicing factor 3A subunit 1 OS=Homo sapiens GN=SF3A1 PE=1 SV=1 - [SF3A1_HUMAN] | 26.10 | 3 | 14 | 88.8 |
| Q15365 | Poly(rC)-binding protein 1 OS=Homo sapiens GN=PCBP1 PE=1 SV=2 - [PCBP1_HUMAN] | 25.84 | 3 | 7 | 37.5 |
| P46783 | 40S ribosomal protein S10 OS=Homo sapiens GN=RPS10 PE=1 SV=1 - [RS10_HUMAN] | 25.45 | 2 | 2 | 18.9 |
| Q00059 | Transcription factor A, mitochondrial OS=Homo sapiens GN=TFAM PE=1 SV=1 - [TFAM_HUMAN] | 25.20 | 1 | 4 | 29.1 |
| O43707 | Alpha-actinin-4 OS=Homo sapiens GN=ACTN4 PE=1 SV=2 - [ACTN4_HUMAN] | 25.03 | 4 | 15 | 104.8 |
| A8MWD9 | Putative small nuclear ribonucleoprotein G-like protein 15 OS=Homo sapiens GN=SNRPGP15 PE=5 SV | 25.00 | 2 | 2 | 8.5 |
| P12956 | X-ray repair cross-complementing protein 6 OS=Homo sapiens GN=XRCC6 PE=1 SV=2 - [XRCC6_HUM | 24.96 | 1 | 9 | 69.8 |
| Q12906 | Interleukin enhancer-binding factor 3 OS=Homo sapiens GN=ILF3 PE=1 SV=3 - [ILF3_HUMAN] | 24.72 | 2 | 14 | 95.3 |
| P12236 | ADP/ATP translocase 3 OS=Homo sapiens GN=SLC25A6 PE=1 SV=4 - [ADT3_HUMAN] | 24.16 | 3 | 1 | 32.8 |
| P62873 | Guanine nucleotide-binding protein G(I)/G(S)/G(T) subunit beta-1 OS=Homo sapiens GN=GNB1 PE=1 | 23.53 | 16 | 6 | 37.4 |
| P37108 | Signal recognition particle 14 kDa protein OS=Homo sapiens GN=SRP14 PE=1 SV=2 - [SRP14_HUMAN | 23.53 | 1 | 3 | 14.6 |
| P09327 | Villin-1 OS=Homo sapiens GN=VIL1 PE=1 SV=4 - [VILI_HUMAN] | 23.46 | 2 | 15 | 92.6 |
| O75964 | ATP synthase subunit g, mitochondrial OS=Homo sapiens GN=ATP5L PE=1 SV=3 - [ATP5L_HUMAN] | 23.30 | 1 | 1 | 11.4 |
| Q9P0L0 | Vesicle-associated membrane protein-associated protein A OS=Homo sapiens GN=VAPA PE=1 SV=3 - | 23.29 | 1 | 1 | 27.9 |
| P43243 | Matrin-3 OS=Homo sapiens GN=MATR3 PE=1 SV=2 - [MATR3_HUMAN] | 23.26 | 3 | 13 | 94.6 |
| P49458 | Signal recognition particle 9 kDa protein OS=Homo sapiens GN=SRP9 PE=1 SV=2 - [SRP09_HUMAN] | 23.26 | 1 | 1 | 10.1 |
| P62826 | GTP-binding nuclear protein Ran OS=Homo sapiens GN=RAN PE=1 SV=3 - [RAN_HUMAN] | 23.15 | 1 | 4 | 24.4 |
| Q12905 | Interleukin enhancer-binding factor 2 OS=Homo sapiens GN=ILF2 PE=1 SV=2 - [ILF2_HUMAN] | 23.08 | 1 | 6 | 43.0 |
| Q13435 | Splicing factor 3B subunit 2 OS=Homo sapiens GN=SF3B2 PE=1 SV=2 - [SF3B2_HUMAN] | 22.68 | 4 | 11 | 100.2 |
| O43809 | Cleavage and polyadenylation specificity factor subunit 5 OS=Homo sapiens GN=NUDT21 PE=1 SV=1 | 22.47 | 1 | 5 | 26.2 |
| P18754 | Regulator of chromosome condensation OS=Homo sapiens GN=RCC1 PE=1 SV=1 - [RCC1_HUMAN] | 22.09 | 1 | 5 | 44.9 |
| O75533 | Splicing factor 3B subunit 1 OS=Homo sapiens GN=SF3B1 PE=1 SV=3 - [SF3B1_HUMAN] | 22.09 | 1 | 22 | 145.7 |
| Q9Y2W1 | Thyroid hormone receptor-associated protein 3 OS=Homo sapiens GN=THRAP3 PE=1 SV=2 - [TR150_ | 22.09 | 5 | 16 | 108.6 |
| Q14978 | Nucleolar and coiled-body phosphoprotein 1 OS=Homo sapiens GN=NOLC1 PE=1 SV=2 - [NOLC1_HU | 21.32 | 1 | 10 | 73.6 |
| P55084 | Trifunctional enzyme subunit beta, mitochondrial OS=Homo sapiens GN=HADHB PE=1 SV=3 - [ECHB_ | 21.31 | 1 | 9 | 51.3 |
| P53999 | Activated RNA polymerase II transcriptional coactivator p15 OS=Homo sapiens GN=SUB1 PE=1 SV=3 | 21.26 | 1 | 3 | 14.4 |
| P35908 | Keratin, type II cytoskeletal 2 epidermal OS=Homo sapiens GN=KRT2 PE=1 SV=2 - [K22E_HUMAN] | 21.13 | 8 | 6 | 65.4 |
| O43684 | Mitotic checkpoint protein BUB3 OS=Homo sapiens GN=BUB3 PE=1 SV=1 - [BUB3_HUMAN] | 21.04 | 1 | 4 | 37.1 |
| Q562R1 | Beta-actin-like protein 2 OS=Homo sapiens GN=ACTBL2 PE=1 SV=2 - [ACTBL_HUMAN] | 21.01 | 3 | 1 | 42.0 |
| P49756 | RNA-binding protein 25 OS=Homo sapiens GN=RBM25 PE=1 SV=3 - [RBM25_HUMAN] | 21.00 | 1 | 13 | 100.1 |
| P46782 | 40S ribosomal protein S5 OS=Homo sapiens GN=RPS5 PE=1 SV=4 - [RS5_HUMAN] | 20.59 | 1 | 2 | 22.9 |
| O00567 | Nucleolar protein 56 OS=Homo sapiens GN=NOP56 PE=1 SV=4 - [NOP56_HUMAN] | 20.03 | 1 | 8 | 66.0 |
| P08579 | U2 small nuclear ribonucleoprotein B'' OS=Homo sapiens GN=SNRPB2 PE=1 SV=1 - [RU2B_HUMAN] | 20.00 | 2 | 3 | 25.5 |
| P24539 | ATP synthase F(0) complex subunit B1, mitochondrial OS=Homo sapiens GN=ATP5F1 PE=1 SV=2 - [A | 19.92 | 1 | 4 | 28.9 |
| Q00839 | Heterogeneous nuclear ribonucleoprotein U OS=Homo sapiens GN=HNRNPU PE=1 SV=6 - [HNRPU_H | 19.76 | 1 | 14 | 90.5 |
| P51571 | Translocon-associated protein subunit delta OS=Homo sapiens GN=SSR4 PE=1 SV=1 - [SSRD_HUMAN | 19.65 | 1 | 3 | 19.0 |
| Q01081 | Splicing factor U2AF 35 kDa subunit OS=Homo sapiens GN=U2AF1 PE=1 SV=3 - [U2AF1_HUMAN] | 19.58 | 2 | 4 | 27.9 |
| P67870 | Casein kinase II subunit beta OS=Homo sapiens GN=CSNK2B PE=1 SV=1 - [CSK2B_HUMAN] | 19.53 | 1 | 2 | 24.9 |
| P63000 | Ras-related C3 botulinum toxin substrate 1 OS=Homo sapiens GN=RAC1 PE=1 SV=1 - [RAC1_HUMAN | 19.27 | 3 | 1 | 21.4 |
| O14979 | Heterogeneous nuclear ribonucleoprotein D-like OS=Homo sapiens GN=HNRNPDL PE=1 SV=3 - [HNR | 19.05 | 8 | 7 | 46.4 |
| P06753 | Tropomyosin alpha-3 chain OS=Homo sapiens GN=TPM3 PE=1 SV=2 - [TPM3_HUMAN] | 18.95 | 4 | 2 | 32.9 |
| P52597 | Heterogeneous nuclear ribonucleoprotein F OS=Homo sapiens GN=HNRNPF PE=1 SV=3 - [HNRPF_HU | 18.55 | 1 | 5 | 45.6 |

| | | | | | |
|---|---|---|---|---|---|
| Q08945 | FACT complex subunit SSRP1 OS=Homo sapiens GN=SSRP1 PE=1 SV=1 - [SSRP1_HUMAN] | 18.48 | 1 | 9 | 81.0 |
| P19338 | Nucleolin OS=Homo sapiens GN=NCL PE=1 SV=3 - [NUCL_HUMAN] | 18.31 | 2 | 11 | 76.6 |
| P27487 | Dipeptidyl peptidase 4 OS=Homo sapiens GN=DPP4 PE=1 SV=2 - [DPP4_HUMAN] | 18.15 | 1 | 9 | 88.2 |
| Q96I25 | Splicing factor 45 OS=Homo sapiens GN=RBM17 PE=1 SV=1 - [SPF45_HUMAN] | 17.96 | 1 | 5 | 44.9 |
| P40939 | Trifunctional enzyme subunit alpha, mitochondrial OS=Homo sapiens GN=HADHA PE=1 SV=2 - [ECHA | 17.82 | 1 | 9 | 82.9 |
| Q13247 | Serine/arginine-rich splicing factor 6 OS=Homo sapiens GN=SRSF6 PE=1 SV=2 - [SRSF6_HUMAN] | 17.73 | 1 | 3 | 39.6 |
| P06576 | ATP synthase subunit beta, mitochondrial OS=Homo sapiens GN=ATP5B PE=1 SV=3 - [ATPB_HUMAN | 17.58 | 1 | 7 | 56.5 |
| Q12874 | Splicing factor 3A subunit 3 OS=Homo sapiens GN=SF3A3 PE=1 SV=1 - [SF3A3_HUMAN] | 17.37 | 1 | 4 | 58.8 |
| Q07666 | KH domain-containing, RNA-binding, signal transduction-associated protein 1 OS=Homo sapiens GN=I | 17.16 | 3 | 6 | 48.2 |
| Q07020 | 60S ribosomal protein L18 OS=Homo sapiens GN=RPL18 PE=1 SV=2 - [RL18_HUMAN] | 17.02 | 1 | 2 | 21.6 |
| O94906 | Pre-mRNA-processing factor 6 OS=Homo sapiens GN=PRPF6 PE=1 SV=1 - [PRP6_HUMAN] | 17.00 | 1 | 10 | 106.9 |
| P10809 | 60 kDa heat shock protein, mitochondrial OS=Homo sapiens GN=HSPD1 PE=1 SV=2 - [CH60_HUMAN | 16.93 | 1 | 4 | 61.0 |
| Q15393 | Splicing factor 3B subunit 3 OS=Homo sapiens GN=SF3B3 PE=1 SV=4 - [SF3B3_HUMAN] | 16.68 | 3 | 14 | 135.5 |
| Q15029 | 116 kDa U5 small nuclear ribonucleoprotein component OS=Homo sapiens GN=EFTUD2 PE=1 SV=1 - | 16.67 | 1 | 11 | 109.4 |
| Q9H307 | Pinin OS=Homo sapiens GN=PNN PE=1 SV=4 - [PININ_HUMAN] | 16.60 | 1 | 8 | 81.6 |
| O60506 | Heterogeneous nuclear ribonucleoprotein Q OS=Homo sapiens GN=SYNCRIP PE=1 SV=2 - [HNRPQ_H | 16.37 | 1 | 3 | 69.6 |
| P13010 | X-ray repair cross-complementing protein 5 OS=Homo sapiens GN=XRCC5 PE=1 SV=3 - [XRCC5_HUN | 16.12 | 2 | 8 | 82.7 |
| O43143 | Putative pre-mRNA-splicing factor ATP-dependent RNA helicase DHX15 OS=Homo sapiens GN=DHX15 | 16.10 | 1 | 11 | 90.9 |
| Q13595 | Transformer-2 protein homolog alpha OS=Homo sapiens GN=TRA2A PE=1 SV=1 - [TRA2A_HUMAN] | 15.96 | 1 | 4 | 32.7 |
| Q15717 | ELAV-like protein 1 OS=Homo sapiens GN=ELAVL1 PE=1 SV=2 - [ELAV1_HUMAN] | 15.95 | 1 | 4 | 36.1 |
| Q6P2Q9 | Pre-mRNA-processing-splicing factor 8 OS=Homo sapiens GN=PRPF8 PE=1 SV=2 - [PRP8_HUMAN] | 15.93 | 1 | 21 | 273.4 |
| Q15287 | RNA-binding protein with serine-rich domain 1 OS=Homo sapiens GN=RNPS1 PE=1 SV=1 - [RNPS1_H | 15.74 | 1 | 2 | 34.2 |
| P13987 | CD59 glycoprotein OS=Homo sapiens GN=CD59 PE=1 SV=1 - [CD59_HUMAN] | 15.63 | 1 | 1 | 14.2 |
| O96019 | Actin-like protein 6A OS=Homo sapiens GN=ACTL6A PE=1 SV=1 - [ACL6A_HUMAN] | 15.62 | 2 | 5 | 47.4 |
| P35659 | Protein DEK OS=Homo sapiens GN=DEK PE=1 SV=1 - [DEK_HUMAN] | 15.47 | 1 | 3 | 42.6 |
| P35232 | Prohibitin OS=Homo sapiens GN=PHB PE=1 SV=1 - [PHB_HUMAN] | 15.44 | 1 | 4 | 29.8 |
| P62277 | 40S ribosomal protein S13 OS=Homo sapiens GN=RPS13 PE=1 SV=2 - [RS13_HUMAN] | 15.23 | 1 | 2 | 17.2 |
| Q9NYF8 | Bcl-2-associated transcription factor 1 OS=Homo sapiens GN=BCLAF1 PE=1 SV=2 - [BCLF1_HUMAN] | 15.22 | 1 | 9 | 106.1 |
| P68104 | Elongation factor 1-alpha 1 OS=Homo sapiens GN=EEF1A1 PE=1 SV=1 - [EF1A1_HUMAN] | 15.15 | 3 | 6 | 50.1 |
| P05023 | Sodium/potassium-transporting ATPase subunit alpha-1 OS=Homo sapiens GN=ATP1A1 PE=1 SV=1 - | 15.05 | 5 | 9 | 112.8 |
| P17096 | High mobility group protein HMG-I/HMG-Y OS=Homo sapiens GN=HMGA1 PE=1 SV=3 - [HMGA1_HUI | 14.95 | 1 | 1 | 11.7 |
| P05386 | 60S acidic ribosomal protein P1 OS=Homo sapiens GN=RPLP1 PE=1 SV=1 - [RLA1_HUMAN] | 14.91 | 2 | 1 | 11.5 |
| Q9UBB9 | Tuftelin-interacting protein 11 OS=Homo sapiens GN=TFIP11 PE=1 SV=1 - [TFP11_HUMAN] | 14.81 | 1 | 6 | 96.8 |
| Q13185 | Chromobox protein homolog 3 OS=Homo sapiens GN=CBX3 PE=1 SV=4 - [CBX3_HUMAN] | 14.75 | 1 | 1 | 20.8 |
| P68363 | Tubulin alpha-1B chain OS=Homo sapiens GN=TUBA1B PE=1 SV=1 - [TBA1B_HUMAN] | 14.63 | 7 | 3 | 50.1 |
| P09661 | U2 small nuclear ribonucleoprotein A' OS=Homo sapiens GN=SNRPA1 PE=1 SV=2 - [RU2A_HUMAN] | 14.51 | 1 | 3 | 28.4 |
| P38919 | Eukaryotic initiation factor 4A-III OS=Homo sapiens GN=EIF4A3 PE=1 SV=4 - [IF4A3_HUMAN] | 14.36 | 1 | 2 | 46.8 |
| P04843 | Dolichyl-diphosphooligosaccharide--protein glycosyltransferase subunit 1 OS=Homo sapiens GN=RPN1 | 14.33 | 1 | 9 | 68.5 |
| P62273 | 40S ribosomal protein S29 OS=Homo sapiens GN=RPS29 PE=1 SV=2 - [RS29_HUMAN] | 14.29 | 1 | 1 | 6.7 |
| Q13838 | Spliceosome RNA helicase DDX39B OS=Homo sapiens GN=DDX39B PE=1 SV=1 - [DX39B_HUMAN] | 14.25 | 2 | 3 | 49.0 |
| P62995 | Transformer-2 protein homolog beta OS=Homo sapiens GN=TRA2B PE=1 SV=1 - [TRA2B_HUMAN] | 14.24 | 1 | 3 | 33.6 |
| Q9Y5B9 | FACT complex subunit SPT16 OS=Homo sapiens GN=SUPT16H PE=1 SV=1 - [SP16H_HUMAN] | 14.23 | 1 | 9 | 119.8 |
| Q9Y2X3 | Nucleolar protein 58 OS=Homo sapiens GN=NOP58 PE=1 SV=1 - [NOP58_HUMAN] | 14.18 | 1 | 4 | 59.5 |
| Q6NXT2 | Histone H3.3C OS=Homo sapiens GN=H3F3C PE=1 SV=3 - [H3C_HUMAN] | 14.07 | 5 | 1 | 15.2 |
| O60264 | SWI/SNF-related matrix-associated actin-dependent regulator of chromatin subfamily A member 5 OS= | 14.07 | 1 | 5 | 121.8 |
| O75643 | U5 small nuclear ribonucleoprotein 200 kDa helicase OS=Homo sapiens GN=SNRNP200 PE=1 SV=2 - [ | 14.00 | 1 | 27 | 244.4 |
| P35637 | RNA-binding protein FUS OS=Homo sapiens GN=FUS PE=1 SV=1 - [FUS_HUMAN] | 13.88 | 2 | 7 | 53.4 |
| A0A0B4J2D9 | Immunoglobulin kappa variable 1D-13 OS=Homo sapiens GN=IGKV1D-13 PE=3 SV=1 - [KVD13_HUM | 13.68 | 1 | 1 | 12.6 |
| P35580 | Myosin-10 OS=Homo sapiens GN=MYH10 PE=1 SV=3 - [MYH10_HUMAN] | 13.61 | 1 | 8 | 228.9 |
| P62829 | 60S ribosomal protein L23 OS=Homo sapiens GN=RPL23 PE=1 SV=1 - [RL23_HUMAN] | 13.57 | 1 | 2 | 14.9 |
| P26599 | Polypyrimidine tract-binding protein 1 OS=Homo sapiens GN=PTBP1 PE=1 SV=1 - [PTBP1_HUMAN] | 13.56 | 5 | 7 | 57.2 |
| P17480 | Nucleolar transcription factor 1 OS=Homo sapiens GN=UBTF PE=1 SV=1 - [UBF1_HUMAN] | 13.48 | 1 | 2 | 89.4 |
| Q99459 | Cell division cycle 5-like protein OS=Homo sapiens GN=CDC5L PE=1 SV=2 - [CDC5L_HUMAN] | 13.47 | 1 | 7 | 92.2 |
| P26368 | Splicing factor U2AF 65 kDa subunit OS=Homo sapiens GN=U2AF2 PE=1 SV=4 - [U2AF2_HUMAN] | 13.47 | 7 | 6 | 53.5 |
| P08238 | Heat shock protein HSP 90-beta OS=Homo sapiens GN=HSP90AB1 PE=1 SV=4 - [HS90B_HUMAN] | 13.40 | 8 | 8 | 83.2 |
| P68400 | Casein kinase II subunit alpha OS=Homo sapiens GN=CSNK2A1 PE=1 SV=1 - [CSK21_HUMAN] | 13.30 | 4 | 2 | 45.1 |
| P31942 | Heterogeneous nuclear ribonucleoprotein H3 OS=Homo sapiens GN=HNRNPH3 PE=1 SV=2 - [HNRH3 | 13.29 | 1 | 1 | 36.9 |
| P08754 | Guanine nucleotide-binding protein G(k) subunit alpha OS=Homo sapiens GN=GNAI3 PE=1 SV=3 - [G | 13.28 | 11 | 1 | 40.5 |
| P16401 | Histone H1.5 OS=Homo sapiens GN=HIST1H1B PE=1 SV=3 - [H15_HUMAN] | 13.27 | 1 | 1 | 22.6 |
| Q08170 | Serine/arginine-rich splicing factor 4 OS=Homo sapiens GN=SRSF4 PE=1 SV=2 - [SRSF4_HUMAN] | 13.16 | 1 | 3 | 56.6 |
| P31930 | Cytochrome b-c1 complex subunit 1, mitochondrial OS=Homo sapiens GN=UQCRC1 PE=1 SV=3 - [QC | 13.13 | 1 | 3 | 52.6 |
| Q99623 | Prohibitin-2 OS=Homo sapiens GN=PHB2 PE=1 SV=2 - [PHB2_HUMAN] | 13.04 | 1 | 2 | 33.3 |
| P62304 | Small nuclear ribonucleoprotein E OS=Homo sapiens GN=SNRPE PE=1 SV=1 - [RUXE_HUMAN] | 13.04 | 1 | 1 | 10.8 |
| O96000 | NADH dehydrogenase [ubiquinone] 1 beta subcomplex subunit 10 OS=Homo sapiens GN=NDUFB10 P | 12.79 | 1 | 2 | 20.8 |
| P49755 | Transmembrane emp24 domain-containing protein 10 OS=Homo sapiens GN=TMED10 PE=1 SV=2 - [ | 12.79 | 1 | 1 | 25.0 |
| Q8TA86 | Retinitis pigmentosa 9 protein OS=Homo sapiens GN=RP9 PE=1 SV=2 - [RP9_HUMAN] | 12.67 | 1 | 2 | 26.1 |
| P07437 | Tubulin beta chain OS=Homo sapiens GN=TUBB PE=1 SV=2 - [TBB5_HUMAN] | 12.61 | 4 | 2 | 49.6 |
| P08621 | U1 small nuclear ribonucleoprotein 70 kDa OS=Homo sapiens GN=SNRNP70 PE=1 SV=2 - [RU17_HUN | 12.59 | 1 | 4 | 51.5 |
| Q14498 | RNA-binding protein 39 OS=Homo sapiens GN=RBM39 PE=1 SV=2 - [RBM39_HUMAN] | 12.45 | 1 | 5 | 59.3 |
| P19105 | Myosin regulatory light chain 12A OS=Homo sapiens GN=MYL12A PE=1 SV=2 - [ML12A_HUMAN] | 12.28 | 2 | 2 | 19.8 |
| Q9Y6C9 | Mitochondrial carrier homolog 2 OS=Homo sapiens GN=MTCH2 PE=1 SV=1 - [MTCH2_HUMAN] | 12.21 | 1 | 2 | 33.3 |
| Q9H0D6 | 5'-3' exoribonuclease 2 OS=Homo sapiens GN=XRN2 PE=1 SV=1 - [XRN2_HUMAN] | 12.21 | 1 | 4 | 108.5 |
| Q07065 | Cytoskeleton-associated protein 4 OS=Homo sapiens GN=CKAP4 PE=1 SV=2 - [CKAP4_HUMAN] | 12.13 | 1 | 3 | 66.0 |
| Q00610 | Clathrin heavy chain 1 OS=Homo sapiens GN=CLTC PE=1 SV=5 - [CLH1_HUMAN] | 12.12 | 2 | 12 | 191.5 |
| Q01780 | Exosome component 10 OS=Homo sapiens GN=EXOSC10 PE=1 SV=2 - [EXOSX_HUMAN] | 12.09 | 1 | 7 | 100.8 |
| P36542 | ATP synthase subunit gamma, mitochondrial OS=Homo sapiens GN=ATP5C1 PE=1 SV=1 - [ATPG_HU | 12.08 | 1 | 1 | 33.0 |
| Q96EU6 | Ribosomal RNA processing protein 36 homolog OS=Homo sapiens GN=RRP36 PE=1 SV=1 - [RRP36_H | 11.97 | 1 | 2 | 29.8 |
| P62140 | Serine/threonine-protein phosphatase PP1-beta catalytic subunit OS=Homo sapiens GN=PPP1CB PE=1 | 11.93 | 3 | 3 | 37.2 |
| P02533 | Keratin, type I cytoskeletal 14 OS=Homo sapiens GN=KRT14 PE=1 SV=4 - [K1C14_HUMAN] | 11.86 | 14 | 1 | 51.5 |
| O75400 | Pre-mRNA-processing factor 40 homolog A OS=Homo sapiens GN=PRPF40A PE=1 SV=2 - [PR40A_HU | 11.70 | 1 | 8 | 108.7 |

| | | | | | |
|---|---|---|---|---|---|
| P62249 | 40S ribosomal protein S16 OS=Homo sapiens GN=RPS16 PE=1 SV=2 - [RS16_HUMAN] | 11.64 | 1 | 2 | 16.4 |
| P61106 | Ras-related protein Rab-14 OS=Homo sapiens GN=RAB14 PE=1 SV=4 - [RAB14_HUMAN] | 11.63 | 16 | 1 | 23.9 |
| Q86X95 | Corepressor interacting with RBPJ 1 OS=Homo sapiens GN=CIR1 PE=1 SV=1 - [CIR1_HUMAN] | 11.56 | 1 | 3 | 52.3 |
| P45880 | Voltage-dependent anion-selective channel protein 2 OS=Homo sapiens GN=VDAC2 PE=1 SV=2 - [VD | 11.22 | 2 | 1 | 31.5 |
| P11166 | Solute carrier family 2, facilitated glucose transporter member 1 OS=Homo sapiens GN=SLC2A1 PE=1 | 11.18 | 1 | 4 | 54.0 |
| Q9Y265 | RuvB-like 1 OS=Homo sapiens GN=RUVBL1 PE=1 SV=1 - [RUVB1_HUMAN] | 11.18 | 1 | 4 | 50.2 |
| P04899 | Guanine nucleotide-binding protein G(i) subunit alpha-2 OS=Homo sapiens GN=GNAI2 PE=1 SV=3 - [ | 10.99 | 11 | 1 | 40.4 |
| Q8WWI1 | LIM domain only protein 7 OS=Homo sapiens GN=LMO7 PE=1 SV=3 - [LMO7_HUMAN] | 10.99 | 1 | 10 | 192.6 |
| P62314 | Small nuclear ribonucleoprotein Sm D1 OS=Homo sapiens GN=SNRPD1 PE=1 SV=1 - [SMD1_HUMAN] | 10.92 | 1 | 1 | 13.3 |
| Q9ULV4 | Coronin-1C OS=Homo sapiens GN=CORO1C PE=1 SV=1 - [COR1C_HUMAN] | 10.76 | 3 | 3 | 53.2 |
| O95292 | Vesicle-associated membrane protein-associated protein B/C OS=Homo sapiens GN=VAPB PE=1 SV=3 | 10.70 | 1 | 1 | 27.2 |
| Q13243 | Serine/arginine-rich splicing factor 5 OS=Homo sapiens GN=SRSF5 PE=1 SV=1 - [SRSF5_HUMAN] | 10.66 | 1 | 1 | 31.2 |
| Q13547 | Histone deacetylase 1 OS=Homo sapiens GN=HDAC1 PE=1 SV=1 - [HDAC1_HUMAN] | 10.58 | 2 | 2 | 55.1 |
| P55265 | Double-stranded RNA-specific adenosine deaminase OS=Homo sapiens GN=ADAR PE=1 SV=4 - [DSR/ | 10.52 | 1 | 9 | 136.0 |
| Q9Y3T9 | Nucleolar complex protein 2 homolog OS=Homo sapiens GN=NOC2L PE=1 SV=4 - [NOC2L_HUMAN] | 10.28 | 1 | 4 | 84.9 |
| Q8N1F7 | Nuclear pore complex protein Nup93 OS=Homo sapiens GN=NUP93 PE=1 SV=2 - [NUP93_HUMAN] | 10.26 | 1 | 6 | 93.4 |
| Q9BY77 | Polymerase delta-interacting protein 3 OS=Homo sapiens GN=POLDIP3 PE=1 SV=2 - [PDIP3_HUMAN | 10.21 | 1 | 2 | 46.1 |
| P78527 | DNA-dependent protein kinase catalytic subunit OS=Homo sapiens GN=PRKDC PE=1 SV=3 - [PRKDC_ | 10.17 | 2 | 30 | 468.8 |
| P35268 | 60S ribosomal protein L22 OS=Homo sapiens GN=RPL22 PE=1 SV=2 - [RL22_HUMAN] | 10.16 | 1 | 1 | 14.8 |
| Q8NCQ2 | Uncharacterized protein CSNK1G2-AS1 OS=Homo sapiens GN=CSNK1G2-AS1 PE=2 SV=2 - [CNAS1_H | 10.14 | 1 | 1 | 16.1 |
| P63244 | Guanine nucleotide-binding protein subunit beta-2-like 1 OS=Homo sapiens GN=GNB2L1 PE=1 SV=3 - | 10.09 | 1 | 2 | 35.1 |
| Q9UKV3 | Apoptotic chromatin condensation inducer in the nucleus OS=Homo sapiens GN=ACIN1 PE=1 SV=2 - | 10.07 | 1 | 10 | 151.8 |
| P63173 | 60S ribosomal protein L38 OS=Homo sapiens GN=RPL38 PE=1 SV=2 - [RL38_HUMAN] | 10.00 | 1 | 1 | 8.2 |
| Q14690 | Protein RRP5 homolog OS=Homo sapiens GN=PDCD11 PE=1 SV=3 - [RRP5_HUMAN] | 9.99 | 1 | 14 | 208.6 |
| P04350 | Tubulin beta-4A chain OS=Homo sapiens GN=TUBB4A PE=1 SV=2 - [TBB4A_HUMAN] | 9.91 | 3 | 1 | 49.6 |
| P21796 | Voltage-dependent anion-selective channel protein 1 OS=Homo sapiens GN=VDAC1 PE=1 SV=2 - [VD | 9.89 | 2 | 2 | 30.8 |
| Q9Y5B6 | PAX3- and PAX7-binding protein 1 OS=Homo sapiens GN=PAXBP1 PE=1 SV=2 - [PAXB1_HUMAN] | 9.71 | 1 | 5 | 104.7 |
| Q00325 | Phosphate carrier protein, mitochondrial OS=Homo sapiens GN=SLC25A3 PE=1 SV=2 - [MPCP_HUMA | 9.67 | 1 | 3 | 40.1 |
| P49916 | DNA ligase 3 OS=Homo sapiens GN=LIG3 PE=1 SV=2 - [DNLI3_HUMAN] | 9.61 | 1 | 5 | 112.8 |
| Q8N684 | Cleavage and polyadenylation specificity factor subunit 7 OS=Homo sapiens GN=CPSF7 PE=1 SV=1 - | 9.55 | 1 | 4 | 52.0 |
| P42677 | 40S ribosomal protein S27 OS=Homo sapiens GN=RPS27 PE=1 SV=3 - [RS27_HUMAN] | 9.52 | 2 | 1 | 9.5 |
| Q9BZE4 | Nucleolar GTP-binding protein 1 OS=Homo sapiens GN=GTPBP4 PE=1 SV=3 - [NOG1_HUMAN] | 9.46 | 1 | 3 | 73.9 |
| O75934 | Pre-mRNA-splicing factor SPF27 OS=Homo sapiens GN=BCAS2 PE=1 SV=1 - [SPF27_HUMAN] | 9.33 | 1 | 2 | 26.1 |
| P62306 | Small nuclear ribonucleoprotein F OS=Homo sapiens GN=SNRPF PE=1 SV=1 - [RUXF_HUMAN] | 9.30 | 1 | 1 | 9.7 |
| Q14151 | Scaffold attachment factor B2 OS=Homo sapiens GN=SAFB2 PE=1 SV=1 - [SAFB2_HUMAN] | 9.23 | 7 | 2 | 107.4 |
| Q07021 | Complement component 1 Q subcomponent-binding protein, mitochondrial OS=Homo sapiens GN=C1 | 9.22 | 1 | 1 | 31.3 |
| O00541 | Pescadillo homolog OS=Homo sapiens GN=PES1 PE=1 SV=1 - [PESC_HUMAN] | 9.18 | 1 | 3 | 68.0 |
| Q15424 | Scaffold attachment factor B1 OS=Homo sapiens GN=SAFB PE=1 SV=4 - [SAFB1_HUMAN] | 9.18 | 7 | 1 | 102.6 |
| Q9P013 | Spliceosome-associated protein CWC15 homolog OS=Homo sapiens GN=CWC15 PE=1 SV=2 - [CWC1 | 9.17 | 1 | 1 | 26.6 |
| O95235 | Kinesin-like protein KIF20A OS=Homo sapiens GN=KIF20A PE=1 SV=1 - [KI20A_HUMAN] | 8.99 | 13 | 5 | 100.2 |

| | | | | | |
|---|---|---|---|---|---|
| P62913 | 60S ribosomal protein L11 OS=Homo sapiens GN=RPL11 PE=1 SV=2 - [RL11_HUMAN] | 8.99 | 1 | 1 | 20.2 |
| P28370 | Probable global transcription activator SNF2L1 OS=Homo sapiens GN=SMARCA1 PE=1 SV=2 - [SMCA1 | 8.92 | 1 | 1 | 122.5 |
| O43795 | Unconventional myosin-Ib OS=Homo sapiens GN=MYO1B PE=1 SV=3 - [MYO1B_HUMAN] | 8.89 | 2 | 5 | 131.9 |
| P13647 | Keratin, type II cytoskeletal 5 OS=Homo sapiens GN=KRT5 PE=1 SV=3 - [K2C5_HUMAN] | 8.81 | 8 | 1 | 62.3 |
| Q96DI7 | U5 small nuclear ribonucleoprotein 40 kDa protein OS=Homo sapiens GN=SNRNP40 PE=1 SV=1 - [SN | 8.68 | 5 | 2 | 39.3 |
| Q13573 | SNW domain-containing protein 1 OS=Homo sapiens GN=SNW1 PE=1 SV=1 - [SNW1_HUMAN] | 8.58 | 1 | 5 | 61.5 |
| Q9UMS4 | Pre-mRNA-processing factor 19 OS=Homo sapiens GN=PRPF19 PE=1 SV=1 - [PRP19_HUMAN] | 8.53 | 1 | 3 | 55.1 |
| P27824 | Calnexin OS=Homo sapiens GN=CANX PE=1 SV=2 - [CALX_HUMAN] | 8.45 | 1 | 4 | 67.5 |
| O43172 | U4/U6 small nuclear ribonucleoprotein Prp4 OS=Homo sapiens GN=PRPF4 PE=1 SV=2 - [PRP4_HUMA | 8.43 | 5 | 1 | 58.4 |
| Q8WXF1 | Paraspeckle component 1 OS=Homo sapiens GN=PSPC1 PE=1 SV=1 - [PSPC1_HUMAN] | 8.41 | 1 | 1 | 58.7 |
| P30049 | ATP synthase subunit delta, mitochondrial OS=Homo sapiens GN=ATP5D PE=1 SV=2 - [ATPD_HUMAI | 8.33 | 1 | 1 | 17.5 |
| Q15428 | Splicing factor 3A subunit 2 OS=Homo sapiens GN=SF3A2 PE=1 SV=2 - [SF3A2_HUMAN] | 8.19 | 1 | 3 | 49.2 |
| Q9NV31 | U3 small nucleolar ribonucleoprotein protein IMP3 OS=Homo sapiens GN=IMP3 PE=1 SV=1 - [IMP3_H | 8.15 | 1 | 1 | 21.8 |
| Q13263 | Transcription intermediary factor 1-beta OS=Homo sapiens GN=TRIM28 PE=1 SV=5 - [TIF1B_HUMAN | 8.02 | 2 | 4 | 88.5 |
| Q13123 | Protein Red OS=Homo sapiens GN=IK PE=1 SV=3 - [RED_HUMAN] | 7.90 | 1 | 2 | 65.6 |
| P23284 | Peptidyl-prolyl cis-trans isomerase B OS=Homo sapiens GN=PPIB PE=1 SV=2 - [PPIB_HUMAN] | 7.87 | 1 | 1 | 23.7 |
| P42285 | Superkiller viralicidic activity 2-like 2 OS=Homo sapiens GN=SKIV2L2 PE=1 SV=3 - [SK2L2_HUMAN] | 7.87 | 1 | 5 | 117.7 |
| Q9BQG0 | Myb-binding protein 1A OS=Homo sapiens GN=MYBBP1A PE=1 SV=2 - [MBB1A_HUMAN] | 7.61 | 1 | 5 | 148.8 |
| Q9HD64 | X antigen family member 1 OS=Homo sapiens GN=XAGE1A PE=2 SV=3 - [XAGE1_HUMAN] | 7.41 | 2 | 1 | 9.1 |
| Q9Y383 | Putative RNA-binding protein Luc7-like 2 OS=Homo sapiens GN=LUC7L2 PE=1 SV=2 - [LC7L2_HUMAN | 7.40 | 2 | 2 | 46.5 |
| P52434 | DNA-directed RNA polymerases I, II, and III subunit RPABC3 OS=Homo sapiens GN=POLR2H PE=1 S | 7.33 | 1 | 1 | 17.1 |
| Q9NVP1 | ATP-dependent RNA helicase DDX18 OS=Homo sapiens GN=DDX18 PE=1 SV=2 - [DDX18_HUMAN] | 7.31 | 1 | 4 | 75.4 |
| P35222 | Catenin beta-1 OS=Homo sapiens GN=CTNNB1 PE=1 SV=1 - [CTNB1_HUMAN] | 7.30 | 2 | 3 | 85.4 |
| P62899 | 60S ribosomal protein L31 OS=Homo sapiens GN=RPL31 PE=1 SV=1 - [RL31_HUMAN] | 7.20 | 1 | 1 | 14.5 |
| P15880 | 40S ribosomal protein S2 OS=Homo sapiens GN=RPS2 PE=1 SV=2 - [RS2_HUMAN] | 7.17 | 1 | 1 | 31.3 |
| A6NIZ1 | Ras-related protein Rap-1b-like protein OS=Homo sapiens PE=2 SV=1 - [RP1BL_HUMAN] | 7.07 | 2 | 1 | 20.9 |
| P0C0S5 | Histone H2A.Z OS=Homo sapiens GN=H2AFZ PE=1 SV=2 - [H2AZ_HUMAN] | 7.03 | 15 | 1 | 13.5 |
| P42704 | Leucine-rich PPR motif-containing protein, mitochondrial OS=Homo sapiens GN=LRPPRC PE=1 SV=3 - | 6.96 | 1 | 8 | 157.8 |
| P62244 | 40S ribosomal protein S15a OS=Homo sapiens GN=RPS15A PE=1 SV=2 - [RS15A_HUMAN] | 6.92 | 1 | 1 | 14.8 |
| Q5JTV8 | Torsin-1A-interacting protein 1 OS=Homo sapiens GN=TOR1AIP1 PE=1 SV=2 - [TOIP1_HUMAN] | 6.86 | 1 | 3 | 66.2 |
| Q9Y230 | RuvB-like 2 OS=Homo sapiens GN=RUVBL2 PE=1 SV=3 - [RUVB2_HUMAN] | 6.70 | 1 | 2 | 51.1 |
| P20042 | Eukaryotic translation initiation factor 2 subunit 2 OS=Homo sapiens GN=EIF2S2 PE=1 SV=2 - [IF2B_ | 6.61 | 1 | 2 | 38.4 |
| O43818 | U3 small nucleolar RNA-interacting protein 2 OS=Homo sapiens GN=RRP9 PE=1 SV=1 - [U3IP2_HUM/ | 6.53 | 1 | 2 | 51.8 |
| P13073 | Cytochrome c oxidase subunit 4 isoform 1, mitochondrial OS=Homo sapiens GN=COX4I1 PE=1 SV=1 | 6.51 | 1 | 1 | 19.6 |
| O75396 | Vesicle-trafficking protein SEC22b OS=Homo sapiens GN=SEC22B PE=1 SV=4 - [SC22B_HUMAN] | 6.51 | 1 | 1 | 24.6 |
| O95232 | Luc7-like protein 3 OS=Homo sapiens GN=LUC7L3 PE=1 SV=2 - [LC7L3_HUMAN] | 6.48 | 1 | 1 | 51.4 |
| P21127 | Cyclin-dependent kinase 11B OS=Homo sapiens GN=CDK11B PE=1 SV=3 - [CD11B_HUMAN] | 6.42 | 2 | 3 | 92.7 |
| P53985 | Monocarboxylate transporter 1 OS=Homo sapiens GN=SLC16A1 PE=1 SV=3 - [MOT1_HUMAN] | 6.40 | 1 | 1 | 53.9 |
| O15160 | DNA-directed RNA polymerases I and III subunit RPAC1 OS=Homo sapiens GN=POLR1C PE=1 SV=1 - | 6.36 | 1 | 2 | 39.2 |
| Q16643 | Drebrin OS=Homo sapiens GN=DBN1 PE=1 SV=4 - [DREB_HUMAN] | 6.32 | 1 | 2 | 71.4 |

| | | | | | |
|---|---|---|---|---|---|
| Q9H5V9 | UPF0428 protein CXorf56 OS=Homo sapiens GN=CXorf56 PE=1 SV=1 - [CX056_HUMAN] | 6.31 | 1 | 1 | 25.6 |
| P14678 | Small nuclear ribonucleoprotein-associated proteins B and B' OS=Homo sapiens GN=SNRPB PE=1 SV= | 6.25 | 2 | 1 | 24.6 |
| P52292 | Importin subunit alpha-1 OS=Homo sapiens GN=KPNA2 PE=1 SV=1 - [IMA1_HUMAN] | 6.24 | 1 | 2 | 57.8 |
| P61586 | Transforming protein RhoA OS=Homo sapiens GN=RHOA PE=1 SV=1 - [RHOA_HUMAN] | 6.22 | 1 | 1 | 21.8 |
| P53007 | Tricarboxylate transport protein, mitochondrial OS=Homo sapiens GN=SLC25A1 PE=1 SV=2 - [TXTP_I | 6.11 | 1 | 1 | 34.0 |
| O14519 | Cyclin-dependent kinase 2-associated protein 1 OS=Homo sapiens GN=CDK2AP1 PE=1 SV=1 - [CDKA | 6.09 | 2 | 1 | 12.4 |
| P38646 | Stress-70 protein, mitochondrial OS=Homo sapiens GN=HSPA9 PE=1 SV=2 - [GRP75_HUMAN] | 6.04 | 1 | 2 | 73.6 |
| Q86YZ3 | Hornerin OS=Homo sapiens GN=HRNR PE=1 SV=2 - [HORN_HUMAN] | 6.04 | 1 | 4 | 282.2 |
| A0A0C4DH42 | Immunoglobulin heavy variable 3-66 OS=Homo sapiens GN=IGHV3-66 PE=3 SV=1 - [HV366_HUMAN] | 6.03 | 23 | 1 | 12.7 |
| P00387 | NADH-cytochrome b5 reductase 3 OS=Homo sapiens GN=CYB5R3 PE=1 SV=3 - [NB5R3_HUMAN] | 5.98 | 1 | 2 | 34.2 |
| Q9UJS0 | Calcium-binding mitochondrial carrier protein Aralar2 OS=Homo sapiens GN=SLC25A13 PE=1 SV=2 - | 5.93 | 2 | 2 | 74.1 |
| P39656 | Dolichyl-diphosphooligosaccharide--protein glycosyltransferase 48 kDa subunit OS=Homo sapiens GN= | 5.92 | 1 | 2 | 50.8 |
| Q9H6R4 | Nucleolar protein 6 OS=Homo sapiens GN=NOL6 PE=1 SV=2 - [NOL6_HUMAN] | 5.85 | 1 | 4 | 127.5 |
| Q2TAY7 | WD40 repeat-containing protein SMU1 OS=Homo sapiens GN=SMU1 PE=1 SV=2 - [SMU1_HUMAN] | 5.85 | 1 | 2 | 57.5 |
| P25490 | Transcriptional repressor protein YY1 OS=Homo sapiens GN=YY1 PE=1 SV=2 - [TYY1_HUMAN] | 5.80 | 2 | 1 | 44.7 |
| Q13601 | KRR1 small subunit processome component homolog OS=Homo sapiens GN=KRR1 PE=1 SV=4 - [KRR | 5.77 | 1 | 1 | 43.6 |
| P62760 | Visinin-like protein 1 OS=Homo sapiens GN=VSNL1 PE=1 SV=2 - [VISL1_HUMAN] | 5.76 | 1 | 1 | 22.1 |
| P22695 | Cytochrome b-c1 complex subunit 2, mitochondrial OS=Homo sapiens GN=UQCRC2 PE=1 SV=3 - [QC | 5.74 | 1 | 2 | 48.4 |
| O60832 | H/ACA ribonucleoprotein complex subunit 4 OS=Homo sapiens GN=DKC1 PE=1 SV=3 - [DKC1_HUMA | 5.64 | 1 | 1 | 57.6 |
| Q15392 | Delta(24)-sterol reductase OS=Homo sapiens GN=DHCR24 PE=1 SV=2 - [DHC24_HUMAN] | 5.62 | 1 | 3 | 60.1 |
| P10092 | Calcitonin gene-related peptide 2 OS=Homo sapiens GN=CALCB PE=1 SV=1 - [CALCB_HUMAN] | 5.51 | 2 | 1 | 13.7 |
| P11940 | Polyadenylate-binding protein 1 OS=Homo sapiens GN=PABPC1 PE=1 SV=2 - [PABP1_HUMAN] | 5.50 | 2 | 1 | 70.6 |
| P41235 | Hepatocyte nuclear factor 4-alpha OS=Homo sapiens GN=HNF4A PE=1 SV=3 - [HNF4A_HUMAN] | 5.49 | 2 | 2 | 52.8 |
| Q9BXP5 | Serrate RNA effector molecule homolog OS=Homo sapiens GN=SRRT PE=1 SV=1 - [SRRT_HUMAN] | 5.48 | 1 | 3 | 100.6 |
| Q9H0A0 | N-acetyltransferase 10 OS=Homo sapiens GN=NAT10 PE=1 SV=2 - [NAT10_HUMAN] | 5.46 | 1 | 3 | 115.7 |
| O00515 | Ladinin-1 OS=Homo sapiens GN=LAD1 PE=1 SV=2 - [LAD1_HUMAN] | 5.42 | 1 | 2 | 57.1 |
| Q3ZCQ8 | Mitochondrial import inner membrane translocase subunit TIM50 OS=Homo sapiens GN=TIMM50 PE= | 5.38 | 1 | 2 | 39.6 |
| P27482 | Calmodulin-like protein 3 OS=Homo sapiens GN=CALML3 PE=1 SV=2 - [CALL3_HUMAN] | 5.37 | 2 | 1 | 16.9 |
| P35998 | 26S protease regulatory subunit 7 OS=Homo sapiens GN=PSMC2 PE=1 SV=3 - [PRS7_HUMAN] | 5.31 | 1 | 1 | 48.6 |
| P11169 | Solute carrier family 2, facilitated glucose transporter member 3 OS=Homo sapiens GN=SLC2A3 PE=2 | 5.24 | 2 | 2 | 53.9 |
| P51532 | Transcription activator BRG1 OS=Homo sapiens GN=SMARCA4 PE=1 SV=2 - [SMCA4_HUMAN] | 5.22 | 3 | 5 | 184.5 |
| P18887 | DNA repair protein XRCC1 OS=Homo sapiens GN=XRCC1 PE=1 SV=2 - [XRCC1_HUMAN] | 5.21 | 1 | 3 | 69.4 |
| P51654 | Glypican-3 OS=Homo sapiens GN=GPC3 PE=1 SV=1 - [GPC3_HUMAN] | 5.17 | 1 | 1 | 65.5 |
| P36578 | 60S ribosomal protein L4 OS=Homo sapiens GN=RPL4 PE=1 SV=5 - [RL4_HUMAN] | 5.15 | 13 | 1 | 47.7 |
| Q9H5K3 | Protein O-mannose kinase OS=Homo sapiens GN=POMK PE=1 SV=1 - [SG196_HUMAN] | 5.14 | 1 | 1 | 40.0 |
| Q9H5Z1 | Probable ATP-dependent RNA helicase DHX35 OS=Homo sapiens GN=DHX35 PE=1 SV=2 - [DHX35_H | 5.12 | 1 | 2 | 78.9 |
| Q99578 | GTP-binding protein Rit2 OS=Homo sapiens GN=RIT2 PE=1 SV=1 - [RIT2_HUMAN] | 5.07 | 1 | 1 | 24.7 |
| Q9H0S4 | Probable ATP-dependent RNA helicase DDX47 OS=Homo sapiens GN=DDX47 PE=1 SV=1 - [DDX47_H | 5.05 | 1 | 2 | 50.6 |
| O94905 | Erlin-2 OS=Homo sapiens GN=ERLIN2 PE=1 SV=1 - [ERLN2_HUMAN] | 5.01 | 2 | 1 | 37.8 |
| Q14684 | Ribosomal RNA processing protein 1 homolog B OS=Homo sapiens GN=RRP1B PE=1 SV=3 - [RRP1B_ | 5.01 | 1 | 2 | 84.4 |
| Q9P2I0 | Cleavage and polyadenylation specificity factor subunit 2 OS=Homo sapiens GN=CPSF2 PE=1 SV=2 - | 4.99 | 1 | 2 | 88.4 |
| Q7L014 | Probable ATP-dependent RNA helicase DDX46 OS=Homo sapiens GN=DDX46 PE=1 SV=2 - [DDX46_H | 4.95 | 1 | 2 | 117.3 |
| O75489 | NADH dehydrogenase [ubiquinone] iron-sulfur protein 3, mitochondrial OS=Homo sapiens GN=NDUFS | 4.92 | 1 | 1 | 30.2 |
| O15523 | ATP-dependent RNA helicase DDX3Y OS=Homo sapiens GN=DDX3Y PE=1 SV=2 - [DDX3Y_HUMAN] | 4.85 | 2 | 1 | 73.1 |
| Q9UJZ1 | Stomatin-like protein 2, mitochondrial OS=Homo sapiens GN=STOML2 PE=1 SV=1 - [STML2_HUMAN] | 4.78 | 1 | 1 | 38.5 |
| P04156 | Major prion protein OS=Homo sapiens GN=PRNP PE=1 SV=1 - [PRIO_HUMAN] | 4.74 | 1 | 1 | 27.6 |
| Q01844 | RNA-binding protein EWS OS=Homo sapiens GN=EWSR1 PE=1 SV=1 - [EWS_HUMAN] | 4.73 | 1 | 2 | 68.4 |
| P50402 | Emerin OS=Homo sapiens GN=EMD PE=1 SV=1 - [EMD_HUMAN] | 4.72 | 1 | 1 | 29.0 |
| Q09028 | Histone-binding protein RBBP4 OS=Homo sapiens GN=RBBP4 PE=1 SV=3 - [RBBP4_HUMAN] | 4.71 | 2 | 1 | 47.6 |
| Q8WXX5 | DnaJ homolog subfamily C member 9 OS=Homo sapiens GN=DNAJC9 PE=1 SV=1 - [DNJC9_HUMAN] | 4.62 | 1 | 1 | 29.9 |
| P84098 | 60S ribosomal protein L19 OS=Homo sapiens GN=RPL19 PE=1 SV=1 - [RL19_HUMAN] | 4.59 | 1 | 1 | 23.5 |
| P56747 | Claudin-6 OS=Homo sapiens GN=CLDN6 PE=1 SV=2 - [CLD6_HUMAN] | 4.55 | 1 | 1 | 23.3 |
| Q92922 | SWI/SNF complex subunit SMARCC1 OS=Homo sapiens GN=SMARCC1 PE=1 SV=3 - [SMRC1_HUMAN | 4.52 | 2 | 3 | 122.8 |
| P0DOX5 | Immunoglobulin gamma-1 heavy chain OS=Homo sapiens PE=1 SV=1 - [IGG1_HUMAN] | 4.45 | 5 | 1 | 49.3 |
| Q8N163 | Cell cycle and apoptosis regulator protein 2 OS=Homo sapiens GN=CCAR2 PE=1 SV=2 - [CCAR2_HUM | 4.44 | 1 | 2 | 102.8 |
| Q9BRX9 | WD repeat domain-containing protein 83 OS=Homo sapiens GN=WDR83 PE=1 SV=1 - [WDR83_HUM | 4.44 | 1 | 1 | 34.3 |
| Q96J88 | Epithelial-stromal interaction protein 1 OS=Homo sapiens GN=EPSTI1 PE=2 SV=2 - [ESIP1_HUMAN] | 4.40 | 1 | 1 | 36.8 |
| P11388 | DNA topoisomerase 2-alpha OS=Homo sapiens GN=TOP2A PE=1 SV=3 - [TOP2A_HUMAN] | 4.38 | 2 | 4 | 174.3 |
| P19784 | Casein kinase II subunit alpha' OS=Homo sapiens GN=CSNK2A2 PE=1 SV=1 - [CSK22_HUMAN] | 4.29 | 3 | 1 | 41.2 |
| Q14980 | Nuclear mitotic apparatus protein 1 OS=Homo sapiens GN=NUMA1 PE=1 SV=2 - [NUMA1_HUMAN] | 4.26 | 1 | 2 | 238.1 |
| Q9GZL7 | Ribosome biogenesis protein WDR12 OS=Homo sapiens GN=WDR12 PE=1 SV=2 - [WDR12_HUMAN] | 4.26 | 1 | 1 | 47.7 |
| P38432 | Coilin OS=Homo sapiens GN=COIL PE=1 SV=1 - [COIL_HUMAN] | 4.17 | 1 | 1 | 62.6 |
| Q9NY12 | H/ACA ribonucleoprotein complex subunit 1 OS=Homo sapiens GN=GAR1 PE=1 SV=1 - [GAR1_HUMA | 4.15 | 1 | 1 | 22.3 |
| Q96HS1 | Serine/threonine-protein phosphatase PGAM5, mitochondrial OS=Homo sapiens GN=PGAM5 PE=1 SV= | 4.15 | 1 | 1 | 32.0 |
| O95639 | Cleavage and polyadenylation specificity factor subunit 4 OS=Homo sapiens GN=CPSF4 PE=1 SV=1 - | 4.09 | 1 | 1 | 30.2 |
| Q14683 | Structural maintenance of chromosomes protein 1A OS=Homo sapiens GN=SMC1A PE=1 SV=2 - [SMC | 4.06 | 1 | 2 | 143.1 |
| Q10570 | Cleavage and polyadenylation specificity factor subunit 1 OS=Homo sapiens GN=CPSF1 PE=1 SV=2 - | 3.95 | 1 | 3 | 160.8 |
| Q06587 | E3 ubiquitin-protein ligase RING1 OS=Homo sapiens GN=RING1 PE=1 SV=2 - [RING1_HUMAN] | 3.94 | 1 | 1 | 42.4 |
| Q53GS9 | U4/U6.U5 tri-snRNP-associated protein 2 OS=Homo sapiens GN=USP39 PE=1 SV=2 - [SNUT2_HUMAN | 3.89 | 1 | 2 | 65.3 |
| P43307 | Translocon-associated protein subunit alpha OS=Homo sapiens GN=SSR1 PE=1 SV=3 - [SSRA_HUMA | 3.85 | 1 | 1 | 32.2 |
| P46977 | Dolichyl-diphosphooligosaccharide--protein glycosyltransferase subunit STT3A OS=Homo sapiens GN= | 3.83 | 1 | 2 | 80.5 |
| P05198 | Eukaryotic translation initiation factor 2 subunit 1 OS=Homo sapiens GN=EIF2S1 PE=1 SV=3 - [IF2A_ | 3.81 | 1 | 1 | 36.1 |
| P17676 | CCAAT/enhancer-binding protein beta OS=Homo sapiens GN=CEBPB PE=1 SV=2 - [CEBPB_HUMAN] | 3.77 | 1 | 1 | 36.1 |
| P00367 | Glutamate dehydrogenase 1, mitochondrial OS=Homo sapiens GN=GLUD1 PE=1 SV=2 - [DHE3_HUMA | 3.76 | 2 | 1 | 61.4 |
| Q03701 | CCAAT/enhancer-binding protein zeta OS=Homo sapiens GN=CEBPZ PE=1 SV=3 - [CEBPZ_HUMAN] | 3.70 | 1 | 2 | 120.9 |
| O00767 | Acyl-CoA desaturase OS=Homo sapiens GN=SCD PE=1 SV=2 - [ACOD_HUMAN] | 3.62 | 1 | 1 | 41.5 |
| Q9BUJ2 | Heterogeneous nuclear ribonucleoprotein U-like protein 1 OS=Homo sapiens GN=HNRNPUL1 PE=1 SV | 3.62 | 1 | 1 | 95.7 |
| Q5SSJ5 | Heterochromatin protein 1-binding protein 3 OS=Homo sapiens GN=HP1BP3 PE=1 SV=1 - [HP1B3_HU | 3.62 | 1 | 2 | 61.2 |
| Q1KMD3 | Heterogeneous nuclear ribonucleoprotein U-like protein 2 OS=Homo sapiens GN=HNRNPUL2 PE=1 SV | 3.61 | 1 | 1 | 85.1 |
| Q9UHR5 | SAP30-binding protein OS=Homo sapiens GN=SAP30BP PE=1 SV=1 - [S30BP_HUMAN] | 3.57 | 1 | 1 | 33.8 |

| | | | | | |
|---|---|---|---|---|---|
| Q9UIG0 | Tyrosine-protein kinase BAZ1B OS=Homo sapiens GN=BAZ1B PE=1 SV=2 - [BAZ1B_HUMAN] | 3.51 | 1 | 5 | 170.8 |
| P04234 | T-cell surface glycoprotein CD3 delta chain OS=Homo sapiens GN=CD3D PE=1 SV=1 - [CD3D_HUMAN | 3.51 | 1 | 1 | 18.9 |
| Q12996 | Cleavage stimulation factor subunit 3 OS=Homo sapiens GN=CSTF3 PE=1 SV=1 - [CSTF3_HUMAN] | 3.49 | 1 | 1 | 82.9 |
| Q96G21 | U3 small nucleolar ribonucleoprotein protein IMP4 OS=Homo sapiens GN=IMP4 PE=1 SV=1 - [IMP4_H | 3.44 | 1 | 1 | 33.7 |
| O94776 | Metastasis-associated protein MTA2 OS=Homo sapiens GN=MTA2 PE=1 SV=1 - [MTA2_HUMAN] | 3.44 | 3 | 1 | 75.0 |
| O75694 | Nuclear pore complex protein Nup155 OS=Homo sapiens GN=NUP155 PE=1 SV=1 - [NU155_HUMAN] | 3.38 | 1 | 4 | 155.1 |
| Q12824 | SWI/SNF-related matrix-associated actin-dependent regulator of chromatin subfamily B member 1 OS= | 3.38 | 1 | 1 | 44.1 |
| O95218 | Zinc finger Ran-binding domain-containing protein 2 OS=Homo sapiens GN=ZRANB2 PE=1 SV=2 - [ZF | 3.33 | 1 | 1 | 37.4 |
| Q14254 | Flotillin-2 OS=Homo sapiens GN=FLOT2 PE=1 SV=2 - [FLOT2_HUMAN] | 3.27 | 1 | 1 | 47.0 |
| Q69YZ2 | Transmembrane protein 200B OS=Homo sapiens GN=TMEM200B PE=2 SV=1 - [T200B_HUMAN] | 3.26 | 1 | 1 | 32.7 |
| P05455 | Lupus La protein OS=Homo sapiens GN=SSB PE=1 SV=2 - [LA_HUMAN] | 3.19 | 1 | 1 | 46.8 |
| Q9UDY2 | Tight junction protein ZO-2 OS=Homo sapiens GN=TJP2 PE=1 SV=2 - [ZO2_HUMAN] | 3.19 | 1 | 2 | 133.9 |
| Q969G3 | SWI/SNF-related matrix-associated actin-dependent regulator of chromatin subfamily E member 1 OS= | 3.16 | 1 | 1 | 46.6 |
| Q9HAY6 | Beta,beta-carotene 15,15'-dioxygenase OS=Homo sapiens GN=BCO1 PE=1 SV=1 - [BCDO1_HUMAN] | 3.11 | 17 | 1 | 62.6 |
| Q92499 | ATP-dependent RNA helicase DDX1 OS=Homo sapiens GN=DDX1 PE=1 SV=2 - [DDX1_HUMAN] | 3.11 | 1 | 1 | 82.4 |
| P63010 | AP-2 complex subunit beta OS=Homo sapiens GN=AP2B1 PE=1 SV=1 - [AP2B1_HUMAN] | 3.09 | 1 | 3 | 104.5 |
| Q92945 | Far upstream element-binding protein 2 OS=Homo sapiens GN=KHSRP PE=1 SV=4 - [FUBP2_HUMAN | 3.09 | 1 | 2 | 73.1 |
| Q14CX7 | N-alpha-acetyltransferase 25, NatB auxiliary subunit OS=Homo sapiens GN=NAA25 PE=1 SV=1 - [NA/ | 3.09 | 18 | 1 | 112.2 |
| O75369 | Filamin-B OS=Homo sapiens GN=FLNB PE=1 SV=2 - [FLNB_HUMAN] | 3.07 | 1 | 5 | 278.0 |
| Q8IX12 | Cell division cycle and apoptosis regulator protein 1 OS=Homo sapiens GN=CCAR1 PE=1 SV=2 - [CCA | 3.04 | 1 | 1 | 132.7 |
| Q9BY49 | Peroxisomal trans-2-enoyl-CoA reductase OS=Homo sapiens GN=PECR PE=1 SV=2 - [PECR_HUMAN] | 2.97 | 1 | 1 | 32.5 |
| Q9HCS7 | Pre-mRNA-splicing factor SYF1 OS=Homo sapiens GN=XAB2 PE=1 SV=2 - [SYF1_HUMAN] | 2.92 | 1 | 1 | 99.9 |
| O60716 | Catenin delta-1 OS=Homo sapiens GN=CTNND1 PE=1 SV=1 - [CTND1_HUMAN] | 2.89 | 1 | 2 | 108.1 |
| Q14839 | Chromodomain-helicase-DNA-binding protein 4 OS=Homo sapiens GN=CHD4 PE=1 SV=2 - [CHD4_HU | 2.88 | 2 | 3 | 217.9 |
| Q8WTT2 | Nucleolar complex protein 3 homolog OS=Homo sapiens GN=NOC3L PE=1 SV=1 - [NOC3L_HUMAN] | 2.88 | 2 | 2 | 92.5 |
| Q5JTH9 | RRP12-like protein OS=Homo sapiens GN=RRP12 PE=1 SV=2 - [RRP12_HUMAN] | 2.85 | 1 | 2 | 143.6 |
| Q9NVI7 | ATPase family AAA domain-containing protein 3A OS=Homo sapiens GN=ATAD3A PE=1 SV=2 - [ATD3 | 2.84 | 1 | 1 | 71.3 |
| Q86YP4 | Transcriptional repressor p66-alpha OS=Homo sapiens GN=GATAD2A PE=1 SV=1 - [P66A_HUMAN] | 2.84 | 1 | 1 | 68.0 |
| P26373 | 60S ribosomal protein L13 OS=Homo sapiens GN=RPL13 PE=1 SV=4 - [RL13_HUMAN] | 2.84 | 1 | 1 | 24.2 |
| O15427 | Monocarboxylate transporter 4 OS=Homo sapiens GN=SLC16A3 PE=1 SV=1 - [MOT4_HUMAN] | 2.80 | 1 | 1 | 49.4 |
| Q09161 | Nuclear cap-binding protein subunit 1 OS=Homo sapiens GN=NCBP1 PE=1 SV=1 - [NCBP1_HUMAN] | 2.78 | 1 | 1 | 91.8 |
| Q9Y4W6 | AFG3-like protein 2 OS=Homo sapiens GN=AFG3L2 PE=1 SV=2 - [AFG32_HUMAN] | 2.76 | 1 | 2 | 88.5 |
| Q99959 | Plakophilin-2 OS=Homo sapiens GN=PKP2 PE=1 SV=2 - [PKP2_HUMAN] | 2.72 | 1 | 1 | 97.4 |
| Q9H0U3 | Magnesium transporter protein 1 OS=Homo sapiens GN=MAGT1 PE=1 SV=1 - [MAGT1_HUMAN] | 2.69 | 1 | 1 | 38.0 |
| O43395 | U4/U6 small nuclear ribonucleoprotein Prp3 OS=Homo sapiens GN=PRPF3 PE=1 SV=2 - [PRPF3_HUM | 2.64 | 1 | 2 | 77.5 |
| Q5T9A4 | ATPase family AAA domain-containing protein 3B OS=Homo sapiens GN=ATAD3B PE=1 SV=1 - [ATD3 | 2.62 | 1 | 1 | 72.5 |
| Q8WWY3 | U4/U6 small nuclear ribonucleoprotein Prp31 OS=Homo sapiens GN=PRPF31 PE=1 SV=2 - [PRP31_HU | 2.61 | 1 | 1 | 55.4 |
| P21333 | Filamin-A OS=Homo sapiens GN=FLNA PE=1 SV=4 - [FLNA_HUMAN] | 2.53 | 1 | 1 | 280.6 |
| Q13200 | 26S proteasome non-ATPase regulatory subunit 2 OS=Homo sapiens GN=PSMD2 PE=1 SV=3 - [PSMD | 2.53 | 1 | 2 | 100.1 |
| Q8TED0 | U3 small nucleolar RNA-associated protein 15 homolog OS=Homo sapiens GN=UTP15 PE=1 SV=3 - [U | 2.51 | 1 | 1 | 58.4 |

| | | | | | |
|---|---|---|---|---|---|
| P35249 | Replication factor C subunit 4 OS=Homo sapiens GN=RFC4 PE=1 SV=2 - [RFC4_HUMAN] | 2.48 | 1 | 1 | 39.7 |
| Q9Y3B9 | RRP15-like protein OS=Homo sapiens GN=RRP15 PE=1 SV=2 - [RRP15_HUMAN] | 2.48 | 1 | 1 | 31.5 |
| Q9C0J8 | pre-mRNA 3' end processing protein WDR33 OS=Homo sapiens GN=WDR33 PE=1 SV=2 - [WDR33_H | 2.47 | 7 | 1 | 145.8 |
| Q92786 | Prospero homeobox protein 1 OS=Homo sapiens GN=PROX1 PE=1 SV=2 - [PROX1_HUMAN] | 2.44 | 1 | 1 | 83.2 |
| Q0VFZ6 | Coiled-coil domain-containing protein 173 OS=Homo sapiens GN=CCDC173 PE=2 SV=2 - [CC173_HUI | 2.36 | 1 | 1 | 66.4 |
| Q6UN15 | Pre-mRNA 3'-end-processing factor FIP1 OS=Homo sapiens GN=FIP1L1 PE=1 SV=1 - [FIP1_HUMAN] | 2.36 | 1 | 1 | 66.5 |
| Q86XP3 | ATP-dependent RNA helicase DDX42 OS=Homo sapiens GN=DDX42 PE=1 SV=1 - [DDX42_HUMAN] | 2.35 | 1 | 1 | 102.9 |
| Q15059 | Bromodomain-containing protein 3 OS=Homo sapiens GN=BRD3 PE=1 SV=1 - [BRD3_HUMAN] | 2.34 | 3 | 2 | 79.5 |
| Q6IPR3 | tRNA wybutosine-synthesizing protein 3 homolog OS=Homo sapiens GN=TYW3 PE=2 SV=2 - [TYW3_ | 2.32 | 1 | 1 | 29.8 |
| P16615 | Sarcoplasmic/endoplasmic reticulum calcium ATPase 2 OS=Homo sapiens GN=ATP2A2 PE=1 SV=1 - [ | 2.30 | 2 | 2 | 114.7 |
| Q13492 | Phosphatidylinositol-binding clathrin assembly protein OS=Homo sapiens GN=PICALM PE=1 SV=2 - [P | 2.30 | 1 | 1 | 70.7 |
| Q9Y4C8 | Probable RNA-binding protein 19 OS=Homo sapiens GN=RBM19 PE=1 SV=3 - [RBM19_HUMAN] | 2.29 | 1 | 2 | 107.3 |
| Q96GQ7 | Probable ATP-dependent RNA helicase DDX27 OS=Homo sapiens GN=DDX27 PE=1 SV=2 - [DDX27_H | 2.26 | 1 | 1 | 89.8 |
| O00159 | Unconventional myosin-Ic OS=Homo sapiens GN=MYO1C PE=1 SV=4 - [MYO1C_HUMAN] | 2.26 | 1 | 1 | 121.6 |
| P04844 | Dolichyl-diphosphooligosaccharide--protein glycosyltransferase subunit 2 OS=Homo sapiens GN=RPN2 | 2.22 | 1 | 1 | 69.2 |
| Q9BUQ8 | Probable ATP-dependent RNA helicase DDX23 OS=Homo sapiens GN=DDX23 PE=1 SV=3 - [DDX23_H | 2.20 | 1 | 1 | 95.5 |
| Q9UKF6 | Cleavage and polyadenylation specificity factor subunit 3 OS=Homo sapiens GN=CPSF3 PE=1 SV=1 - | 2.19 | 1 | 1 | 77.4 |
| O94973 | AP-2 complex subunit alpha-2 OS=Homo sapiens GN=AP2A2 PE=1 SV=2 - [AP2A2_HUMAN] | 2.13 | 2 | 1 | 103.9 |
| P46087 | Probable 28S rRNA (cytosine(4447)-C(5))-methyltransferase OS=Homo sapiens GN=NOP2 PE=1 SV=2 | 2.09 | 1 | 1 | 89.2 |
| P46013 | Antigen KI-67 OS=Homo sapiens GN=MKI67 PE=1 SV=2 - [KI67_HUMAN] | 2.06 | 1 | 1 | 358.5 |
| P49792 | E3 SUMO-protein ligase RanBP2 OS=Homo sapiens GN=RANBP2 PE=1 SV=2 - [RBP2_HUMAN] | 2.02 | 5 | 4 | 358.0 |
| Q16630 | Cleavage and polyadenylation specificity factor subunit 6 OS=Homo sapiens GN=CPSF6 PE=1 SV=2 - | 2.00 | 1 | 1 | 59.2 |
| Q96FV9 | THO complex subunit 1 OS=Homo sapiens GN=THOC1 PE=1 SV=1 - [THOC1_HUMAN] | 1.98 | 1 | 1 | 75.6 |
| Q14739 | Lamin-B receptor OS=Homo sapiens GN=LBR PE=1 SV=2 - [LBR_HUMAN] | 1.95 | 1 | 1 | 70.7 |
| Q15042 | Rab3 GTPase-activating protein catalytic subunit OS=Homo sapiens GN=RAB3GAP1 PE=1 SV=3 - [RB | 1.94 | 1 | 2 | 110.5 |
| O14647 | Chromodomain-helicase-DNA-binding protein 2 OS=Homo sapiens GN=CHD2 PE=1 SV=2 - [CHD2_HU | 1.91 | 2 | 1 | 211.2 |
| P39900 | Macrophage metalloelastase OS=Homo sapiens GN=MMP12 PE=1 SV=1 - [MMP12_HUMAN] | 1.91 | 1 | 1 | 54.0 |
| O75955 | Flotillin-1 OS=Homo sapiens GN=FLOT1 PE=1 SV=3 - [FLOT1_HUMAN] | 1.87 | 1 | 1 | 47.3 |
| Q13427 | Peptidyl-prolyl cis-trans isomerase G OS=Homo sapiens GN=PPIG PE=1 SV=2 - [PPIG_HUMAN] | 1.86 | 1 | 1 | 88.6 |
| Q8TEM1 | Nuclear pore membrane glycoprotein 210 OS=Homo sapiens GN=NUP210 PE=1 SV=3 - [PO210_HUM | 1.85 | 1 | 2 | 205.0 |
| Q15389 | Angiopoietin-1 OS=Homo sapiens GN=ANGPT1 PE=1 SV=2 - [ANGP1_HUMAN] | 1.81 | 1 | 1 | 57.5 |
| P16435 | NADPH--cytochrome P450 reductase OS=Homo sapiens GN=POR PE=1 SV=2 - [NCPR_HUMAN] | 1.77 | 1 | 1 | 76.6 |
| P12270 | Nucleoprotein TPR OS=Homo sapiens GN=TPR PE=1 SV=3 - [TPR_HUMAN] | 1.74 | 1 | 1 | 267.1 |
| O00505 | Importin subunit alpha-4 OS=Homo sapiens GN=KPNA3 PE=1 SV=2 - [IMA4_HUMAN] | 1.73 | 1 | 1 | 57.8 |
| Q13206 | Probable ATP-dependent RNA helicase DDX10 OS=Homo sapiens GN=DDX10 PE=1 SV=2 - [DDX10_H | 1.71 | 1 | 1 | 100.8 |
| Q9GZU2 | Paternally-expressed gene 3 protein OS=Homo sapiens GN=PEG3 PE=1 SV=1 - [PEG3_HUMAN] | 1.70 | 1 | 1 | 180.7 |
| Q96KR1 | Zinc finger RNA-binding protein OS=Homo sapiens GN=ZFR PE=1 SV=2 - [ZFR_HUMAN] | 1.68 | 1 | 1 | 116.9 |
| Q9BQ04 | RNA-binding protein 4B OS=Homo sapiens GN=RBM4B PE=1 SV=1 - [RBM4B_HUMAN] | 1.67 | 2 | 1 | 40.1 |
| O14776 | Transcription elongation regulator 1 OS=Homo sapiens GN=TCERG1 PE=1 SV=2 - [TCRG1_HUMAN] | 1.64 | 1 | 1 | 123.8 |
| Q12788 | Transducin beta-like protein 3 OS=Homo sapiens GN=TBL3 PE=1 SV=2 - [TBL3_HUMAN] | 1.61 | 1 | 1 | 89.0 |

| | | | | | |
|---|---|---|---|---|---|
| Q13428 | Treacle protein OS=Homo sapiens GN=TCOF1 PE=1 SV=3 - [TCOF_HUMAN] | 1.61 | 1 | 1 | 152.0 |
| Q9ULW0 | Targeting protein for Xklp2 OS=Homo sapiens GN=TPX2 PE=1 SV=2 - [TPX2_HUMAN] | 1.61 | 1 | 1 | 85.6 |
| Q86U86 | Protein polybromo-1 OS=Homo sapiens GN=PBRM1 PE=1 SV=1 - [PB1_HUMAN] | 1.60 | 1 | 1 | 192.8 |
| Q15637 | Splicing factor 1 OS=Homo sapiens GN=SF1 PE=1 SV=4 - [SF01_HUMAN] | 1.56 | 1 | 1 | 68.3 |
| P17735 | Tyrosine aminotransferase OS=Homo sapiens GN=TAT PE=1 SV=1 - [ATTY_HUMAN] | 1.54 | 1 | 1 | 50.4 |
| P33991 | DNA replication licensing factor MCM4 OS=Homo sapiens GN=MCM4 PE=1 SV=5 - [MCM4_HUMAN] | 1.51 | 1 | 1 | 96.5 |
| Q92797 | Symplekin OS=Homo sapiens GN=SYMPK PE=1 SV=2 - [SYMPK_HUMAN] | 1.49 | 1 | 1 | 141.1 |
| Q9NY61 | Protein AATF OS=Homo sapiens GN=AATF PE=1 SV=1 - [AATF_HUMAN] | 1.43 | 1 | 1 | 63.1 |
| Q86SQ6 | Adhesion G protein-coupled receptor A1 OS=Homo sapiens GN=ADGRA1 PE=2 SV=3 - [AGRA1_HUMA | 1.43 | 1 | 1 | 60.8 |
| Q9BVJ6 | U3 small nucleolar RNA-associated protein 14 homolog A OS=Homo sapiens GN=UTP14A PE=1 SV=1 | 1.43 | 1 | 1 | 87.9 |
| Q9BZJ0 | Crooked neck-like protein 1 OS=Homo sapiens GN=CRNKL1 PE=1 SV=4 - [CRNL1_HUMAN] | 1.42 | 1 | 1 | 100.4 |
| Q9UQE7 | Structural maintenance of chromosomes protein 3 OS=Homo sapiens GN=SMC3 PE=1 SV=2 - [SMC3_ | 1.40 | 1 | 1 | 141.5 |
| P42701 | Interleukin-12 receptor subunit beta-1 OS=Homo sapiens GN=IL12RB1 PE=1 SV=1 - [I12R1_HUMAN] | 1.36 | 1 | 1 | 73.1 |
| Q96NH3 | Protein broad-minded OS=Homo sapiens GN=TBC1D32 PE=2 SV=4 - [BROMI_HUMAN] | 1.35 | 6 | 1 | 144.7 |
| Q9Y5Q0 | Fatty acid desaturase 3 OS=Homo sapiens GN=FADS3 PE=2 SV=1 - [FADS3_HUMAN] | 1.35 | 1 | 1 | 51.1 |
| Q92851 | Caspase-10 OS=Homo sapiens GN=CASP10 PE=1 SV=3 - [CASPA_HUMAN] | 1.34 | 1 | 1 | 58.9 |
| Q9BRR9 | Rho GTPase-activating protein 9 OS=Homo sapiens GN=ARHGAP9 PE=1 SV=2 - [RHG09_HUMAN] | 1.33 | 1 | 1 | 83.2 |
| Q8NFH3 | Nucleoporin Nup43 OS=Homo sapiens GN=NUP43 PE=1 SV=1 - [NUP43_HUMAN] | 1.32 | 3 | 1 | 42.1 |
| Q8TAM2 | Tetratricopeptide repeat protein 8 OS=Homo sapiens GN=TTC8 PE=1 SV=2 - [TTC8_HUMAN] | 1.29 | 1 | 1 | 61.5 |
| Q9H6N6 | Putative uncharacterized protein MYH16 OS=Homo sapiens GN=MYH16 PE=1 SV=2 - [MYH16_HUMAI | 1.28 | 3 | 1 | 128.2 |
| Q00266 | S-adenosylmethionine synthase isoform type-1 OS=Homo sapiens GN=MAT1A PE=1 SV=2 - [METK1_I | 1.27 | 3 | 1 | 43.6 |
| Q9Y6W6 | Dual specificity protein phosphatase 10 OS=Homo sapiens GN=DUSP10 PE=1 SV=1 - [DUS10_HUMAN | 1.24 | 1 | 1 | 52.6 |
| P57740 | Nuclear pore complex protein Nup107 OS=Homo sapiens GN=NUP107 PE=1 SV=1 - [NU107_HUMAN] | 1.19 | 1 | 1 | 106.3 |
| Q9NQW1 | Protein transport protein Sec31B OS=Homo sapiens GN=SEC31B PE=1 SV=1 - [SC31B_HUMAN] | 1.19 | 2 | 1 | 128.6 |
| Q15051 | IQ calmodulin-binding motif-containing protein 1 OS=Homo sapiens GN=IQCB1 PE=1 SV=1 - [IQCB1_ | 1.17 | 1 | 1 | 68.9 |
| Q68DV7 | E3 ubiquitin-protein ligase RNF43 OS=Homo sapiens GN=RNF43 PE=1 SV=1 - [RNF43_HUMAN] | 1.15 | 1 | 1 | 85.7 |
| O75691 | Small subunit processome component 20 homolog OS=Homo sapiens GN=UTP20 PE=1 SV=3 - [UTP2 | 1.15 | 1 | 1 | 318.2 |
| P01024 | Complement C3 OS=Homo sapiens GN=C3 PE=1 SV=2 - [CO3_HUMAN] | 1.14 | 1 | 1 | 187.0 |
| Q92621 | Nuclear pore complex protein Nup205 OS=Homo sapiens GN=NUP205 PE=1 SV=3 - [NU205_HUMAN] | 1.14 | 1 | 1 | 227.8 |
| Q92484 | Acid sphingomyelinase-like phosphodiesterase 3a OS=Homo sapiens GN=SMPDL3A PE=1 SV=2 - [ASN | 1.10 | 1 | 1 | 51.2 |
| Q6PI77 | Protein BHLHb9 OS=Homo sapiens GN=BHLHB9 PE=2 SV=1 - [BHLH9_HUMAN] | 1.10 | 1 | 1 | 60.3 |
| P49761 | Dual specificity protein kinase CLK3 OS=Homo sapiens GN=CLK3 PE=1 SV=3 - [CLK3_HUMAN] | 1.10 | 1 | 1 | 73.5 |
| Q9H2P0 | Activity-dependent neuroprotector homeobox protein OS=Homo sapiens GN=ADNP PE=1 SV=1 - [ADI | 1.09 | 1 | 1 | 123.5 |
| P02751 | Fibronectin OS=Homo sapiens GN=FN1 PE=1 SV=4 - [FINC_HUMAN] | 1.09 | 1 | 2 | 262.5 |
| Q9BVP2 | Guanine nucleotide-binding protein-like 3 OS=Homo sapiens GN=GNL3 PE=1 SV=2 - [GNL3_HUMAN] | 1.09 | 1 | 1 | 62.0 |
| Q5UIP0 | Telomere-associated protein RIF1 OS=Homo sapiens GN=RIF1 PE=1 SV=2 - [RIF1_HUMAN] | 1.09 | 1 | 1 | 274.3 |
| Q8IXK0 | Polyhomeotic-like protein 2 OS=Homo sapiens GN=PHC2 PE=1 SV=1 - [PHC2_HUMAN] | 1.05 | 1 | 1 | 90.7 |
| Q8WUM0 | Nuclear pore complex protein Nup133 OS=Homo sapiens GN=NUP133 PE=1 SV=2 - [NU133_HUMAN] | 1.04 | 1 | 1 | 128.9 |
| Q3MJ13 | WD repeat-containing protein 72 OS=Homo sapiens GN=WDR72 PE=2 SV=2 - [WDR72_HUMAN] | 1.00 | 1 | 1 | 123.3 |
| O15078 | Centrosomal protein of 290 kDa OS=Homo sapiens GN=CEP290 PE=1 SV=2 - [CE290_HUMAN] | 0.93 | 2 | 1 | 290.2 |

| | | | | | |
|---|---|---|---|---|---|
| A8K2U0 | Alpha-2-macroglobulin-like protein 1 OS=Homo sapiens GN=A2ML1 PE=1 SV=3 - [A2ML1_HUMAN] | 0.89 | 1 | 1 | 161.0 |
| P49790 | Nuclear pore complex protein Nup153 OS=Homo sapiens GN=NUP153 PE=1 SV=2 - [NU153_HUMAN] | 0.88 | 1 | 1 | 153.8 |
| Q9H2G4 | Testis-specific Y-encoded-like protein 2 OS=Homo sapiens GN=TSPYL2 PE=1 SV=1 - [TSYL2_HUMAN] | 0.87 | 1 | 1 | 79.4 |
| Q9C0G6 | Dynein heavy chain 6, axonemal OS=Homo sapiens GN=DNAH6 PE=1 SV=3 - [DYH6_HUMAN] | 0.84 | 1 | 1 | 475.7 |
| P26358 | DNA (cytosine-5)-methyltransferase 1 OS=Homo sapiens GN=DNMT1 PE=1 SV=2 - [DNMT1_HUMAN] | 0.74 | 1 | 1 | 183.0 |
| Q07157 | Tight junction protein ZO-1 OS=Homo sapiens GN=TJP1 PE=1 SV=3 - [ZO1_HUMAN] | 0.69 | 1 | 1 | 195.3 |
| Q8NFU7 | Methylcytosine dioxygenase TET1 OS=Homo sapiens GN=TET1 PE=1 SV=2 - [TET1_HUMAN] | 0.66 | 1 | 1 | 235.2 |
| Q14571 | Inositol 1,4,5-trisphosphate receptor type 2 OS=Homo sapiens GN=ITPR2 PE=1 SV=2 - [ITPR2_HUM/ | 0.63 | 1 | 1 | 307.9 |
| Q9NYQ6 | Cadherin EGF LAG seven-pass G-type receptor 1 OS=Homo sapiens GN=CELSR1 PE=1 SV=1 - [CELR1 | 0.56 | 1 | 1 | 329.3 |
| P51610 | Host cell factor 1 OS=Homo sapiens GN=HCFC1 PE=1 SV=2 - [HCFC1_HUMAN] | 0.54 | 1 | 1 | 208.6 |
| O15067 | Phosphoribosylformylglycinamidine synthase OS=Homo sapiens GN=PFAS PE=1 SV=4 - [PUR4_HUMA | 0.45 | 2 | 1 | 144.6 |
| Q15154 | Pericentriolar material 1 protein OS=Homo sapiens GN=PCM1 PE=1 SV=4 - [PCM1_HUMAN] | 0.30 | 1 | 1 | 228.4 |
| Q8WXX0 | Dynein heavy chain 7, axonemal OS=Homo sapiens GN=DNAH7 PE=1 SV=2 - [DYH7_HUMAN] | 0.17 | 1 | 1 | 460.9 |

**Figure S1 A. TARDBP left and right arms (1220 bp).**

(Black: Left arm of TARDBP, Black: Right arm of TARDBP, Black: gRNA, White: PAM)

GTTCTTTACACCTGGTTCACTGCCCATATTCAGTCTTCAGGTTCAGCTGATGGATCGC
TTGCTTAGGAAAATCGACTGGGACCTATCACGCCCATGCCTCAGCCAGTTAGGCTCC
TCCTCTGCCTTCAGGATTACTTAACGACTAACCCGATTGTCATAAAATAGCTACTGTTT
ATTGTTCCCTTGCATACCCTAGCTTGTAACCAGCAAGCCACGTTGGACTCACAGTTAC
AGTTTCAGTACCTGACGCATCATAAGCCTTCAGGGAAAGTTTTTTGAATGAATGAAC
TGGCGAGGCATCACATTTTGATAGGAAATCACTACCCTTACCTTCACCTCGTCATTTT
TCAGGGATAACCAATGCATATACGAATCCAGACAAGCATTTTTCTGGAAGTCAGAAC
TCTGACATGGTTTGGGTATTATCATTATAAGGAAACAGTTATTCTGACATGAATGTTGT
TCATTCATATCTCTTTTCTCTTTAGGAAAAGTAAAAGATGTCTGAATATATTCGGGTAA
guide 3
CCGAAGATGAGAACGATGAGCCCATTGAAATACCATCGGAAGACGATGGACGGTG
guide 1
CTGCTCTCCACGGTTACAGCCCAGTTTCCAGGGGCGTGTGGGCTTCGCTACAGGAAT
CCAGTGTCTCAGTGTATGAGAGGTGTCCGGCTGGTAGAAGGAATTCTGCATGCCCCA
GATGCTGGCTGGGGAAATCTGGTGTATGTTGTCAACTATCCAAAAGGTTTGTTACCAT
TTGGTTTTTGTAATCATGCTGAAGTGTGTTCAGGTGTGTGTCTCATCCATGGATCTTA
GCCTCTTTTGGTGGCAGAATGTCTCCTGAAACTTAAGTATTTCCTTGAACTTCAGTGT
TAGACAAGTTTGGAAGACCACGAGTCTAAATTTATGGAAACTGGGGATTGTAGATCA
TTTTAAAAGAGAAAACAATGTATTTAAAAAATATCTGAATAAACTTTGCCAAGTTGAT
GATAAATTTCAGGAATCACTAAGAATGTTTTGTACATAAATCATTGTTGTAAATAGATT
CTTCTGAAGTGCTTCAGTGTTTGGATTTGATCATTGTCCTTTTGGTGTTACATCATTCA
TTCAGCAAATCTGAGTTCATTTTTTCCCTTAAATGAGCAAACAGTTGGAGTAGCCAA
ATCTTGAATTAGTGCAGTTCCAATTACAATTTCGAGGTTCTGGTCTTATCCTTCTCTCT
GCCTC

**Figure S1 B.TARDBP puro-DD knock-in cassette (2252 bp).**

(Black: Left arm of TARDBP, Blue: Puro, Orange: P2A, Red: DD-tag, Sky blue: 3xGGGGS, Black: Right arm of TARDBP, Black: point mutation site of PAM sequences)

GTTCTTTACACCTGGTTCACTGCCCATATTCAGTCTTCAGGTTCAGCTGATGGATCGC
TTGCTTAGGAAAATCGACTGGGACCTATCACGCCCATGCCTCAGCCAGTTAGGCTCC
TCCTCTGCCTTCAGGATTACTTAACGACTAACCCGATTGTCATAAAATAGCTACTGTTT
ATTGTTCCCTTGCATACCCTAGCTTGTAACCAGCAAGCCACGTTGGACTCACAGTTAC
AGTTTCAGTACCTGACGCATCATAAGCCTTCAGGGAAAGTTTTTGAATGAATGAAC
TGGCGAGGCATCACATTTTGATAGGAAATCACTACCCTTACCTTCACCTCGTCATTTT
TCAGGGATAACCAATGCATATACGAATCCAGACAAGCATTTTTCTGGAAGTCAGAAC
TCTGACATGGTTTGGGTATTATCATTATAAGGAAACAGTTATTCTGACATGAATGTTGT
TCATTCATATCTCTTTTCTCTTTAGGAAAAGTAAAAGATGACCGAGTACAAGCCCACG
GTGCGCCTCGCCACCCGCGACGACGTCCCCAGGGCCGTACGCACCCTCGCCGCCGC
GTTCGCCGACTACCCCGCCACGCGCCACACCGTCGATCCGGACCGCCACATCGAGC
GGGTCACCGAGCTGCAAGAACTCTTCCTCACGCGCGTCGGGCTCGACATCGGCAAG
GTGTGGGTCGCGGACGACGGCGCCGCGGTGGCGGTCTGGACCACGCCGGAGAGCG
TCGAAGCGGGGGCGGTGTTCGCCGAGATCGGCCCGCGCATGGCCGAGTTGAGCGGT
TCCCGGCTGGCCGCGCAGCAACAGATGGAAGGCCTCCTGGCGCCGCACCGGCCCAA
GGAGCCCGCGTGGTTCCTGGCCACCGTCGGCGTCTCGCCCGACCACCAGGGCAAGG
GTCTGGGCAGCGCCGTCGTGCTCCCCGGAGTGGAGGCGGCCGAGCGCGCCGGGGT
GCCCGCCTTCCTGGAGACCTCCGCGCCCCGCAACCTCCCCTTCTACGAGCGGCTCGG
CTTCACCGTCACCGCCGACGTCGAGGTGCCCGAAGGACCGCGCACCTGGTGCATGA
CCCGCAAGCCCGGTGCCGGATCCGGAGCCACGAACTTCTCTCTGTTAAAGCAAGCA
GGAGACGTGGAAGAAAACCCCGGTCCCATGGGAGTGCAGGTGGAAACCATCTCCCC
AGGAGACGGGCGCACCTTCCCCAAGCGCGGCCAGACCTGTGTGGTGCACTACACCG
GGATGCTTGAAGATGGAAAGAAAGTCGATTCCTCCCGGGACAGAAACAAGCCCTTT
AAGTTTATGCTAGGCAAGCAGGAGGTGATCCGAGGCTGGGAAGAAGGGGTTGCCCA
GATGAGTGTGGGTCAGAGAGCCAAACTGACTATATCTCCAGATTATGCCTATGGTGCC
ACTGGGCACCCAGGCATCATCCCACCACATGCCACTCTCGTCTTCGATGTGGAGCTT
CTAAAACCGGAAGGTGGAGGTGGATCTGGTGGAGGTGGATCTGGTGGCGGCGGTTC
AATGTCTGAATATATTCGGGTAACCGAAGATGAGAACGATGAGCCCATTGAAATACC
ATCGGAAGACGATGGGACGGTGCTGCTCTCCACAGTTACAGCCCAGTTTCCAGGGG
CGTGTGGGCTTCGCTACAGAAATCCAGTGTCTCAGTGTATGAGAGGTGTCCGGCTGG
TAGAAGGAATTCTGCATGCCCCAGATGCTGGCTGGGGAAATCTGGTGTATGTTGTCA
ACTATCCAAAAGGTTTGTTACCATTTGGTTTTTGTAATCATGCTGAAGTGTGTTCAGG
TGTGTGTCTCATCCATGGATCTTAGCCTCTTTTGGTGGCAGAATGTCTCCTGAAACTT

AAGTATTTCCTTGAACTTCAGTGTTAGACAAGTTTGGAAGACCACGAGTCTAAATTT
ATGGAAACTGGGGATTGTAGATCATTTTAAAAGAGAAAACAATGTATTTAAAAAATAT
CTGAATAAACTTTGCCAAGTTGATGATAAATTTCAGGAATCACTAAGAATGTTTTGTA
CATAAATCATTGTTGTAAATAGATTCTTCTGAAGTGCTTCAGTGTTTGGATTTGATCAT
TGTCCTTTTGGTGTTACATCATTCATTCAGCAAATCTGAGTTCATTTTTTCCCTTAAAT
GAGCAAACAGTTGGAGTAGCCAAATCTTGAATTAGTGCAGTTCCAATTACAATTTCG
AGGTTCTGGTCTTATCCTTCTCTCTGCCTC

**Figure S1 C.TARDBP bla-DD knock-in cassette (2051 bp).**

(Black: Left arm of TARDBP, Blue: bla, Orange: P2A, Red: DD-tag, Sky blue: 3xGGGGS, Black: Right arm of TARDBP, Black: point mutation site of PAM sequences)

GTTCTTTACACCTGGTTCACTGCCCATATTCAGTCTTCAGGTTCAGCTGATGGATCGC
TTGCTTAGGAAAATCGACTGGGACCTATCACGCCCATGCCTCAGCCAGTTAGGCTCC
TCCTCTGCCTTCAGGATTACTTAACGACTAACCCGATTGTCATAAAATAGCTACTGTTT
ATTGTTCCCTTGCATACCCTAGCTTGTAACCAGCAAGCCACGTTGGACTCACAGTTAC
AGTTTCAGTACCTGACGCATCATAAGCCTTCAGGGAAAGTTTTTTGAATGAATGAAC
TGGCGAGGCATCACATTTTGATAGGAAATCACTACCCTTACCTTCACCTCGTCATTTT
TCAGGGATAACCAATGCATATACGAATCCAGACAAGCATTTTTCTGGAAGTCAGAAC
TCTGACATGGTTTGGGTATTATCATTATAAGGAAACAGTTATTCTGACATGAATGTTGT
TCATTCATATCTCTTTTCTCTTTAGGAAAAGTAAAAGATGGCCAAGCCTTTGTCTCAA
GAAGAATCCACCCTCATTGAAAGAGCAACGGCTACAATCAACAGCATCCCCATCTCT
GAAGACTACAGCGTCGCCAGCGCAGCTCTCTCTAGCGACGGCCGCATCTTCACTGGT
GTCAATGTATATCATTTTACTGGGGGACCTTGTGCAGAACTCGTGGTGCTGGGCACTG
CTGCTGCTGCGGCAGCTGGCAACCTGACTTGTATCGTCGCGATCGGAAATGAGAACA
GGGGCATCTTGAGCCCCTGCGGACGGTGCCGACAGGTGCTTCTCGATCTGCATCCTG
GGATCAAAGCCATAGTGAAGGACAGTGATGGACAGCCGACGGCAGTTGGGATTCGT
GAATTGCTGCCCTCTGGTTATGTGTGGGAGGGCGGATCCGGAGCCACGAACTTCTCT
CTGTTAAAGCAAGCAGGAGACGTGGAAGAAAACCCCGGTCCCATGGGAGTGCAGG
TGGAAACCATCTCCCCAGGAGACGGGCGCACCTTCCCCAAGCGCGGCCAGACCTGT
GTGGTGCACTACACCGGGATGCTTGAAGATGGAAAGAAAGTCGATTCCTCCCGGGA
CAGAAACAAGCCCTTTAAGTTTATGCTAGGCAAGCAGGAGGTGATCCGAGGCTGGG
AAGAAGGGGTTGCCCAGATGAGTGTGGGTCAGAGAGCCAAACTGACTATATCTCCA
GATTATGCCTATGGTGCCACTGGGCACCCAGGCATCATCCCACCACATGCCACTCTCG
TCTTCGATGTGGAGCTTCTAAAACCGGAAGGTGGAGGTGGATCTGGTGGAGGTGGA
TCTGGTGGCGGCGGTTCAATGTCTGAATATATTCGGGTAACCGAAGATGAGAACGAT
GAGCCCATTGAAATACCATCGGAAGACGATGGGACGGTGCTGCTCTCCACAGTTACA
GCCCAGTTTCCAGGGGCGTGTGGGCTTCGCTACAGAAATCCAGTGTCTCAGTGTATG
AGAGGTGTCCGGCTGGTAGAAGGAATTCTGCATGCCCCAGATGCTGGCTGGGAAA
TCTGGTGTATGTTGTCAACTATCCAAAAGGTTTGTTACCATTTGGTTTTTGTAATCATG
CTGAAGTGTGTTCAGGTGTGTGTCTCATCCATGGATCTTAGCCTCTTTTGGTGGCAGA
ATGTCTCCTGAAACTTAAGTATTTCCTTGAACTTCAGTGTTAGACAAGTTTGGAAGAC
CACGAGTCTAAATTTATGGAAACTGGGGATTGTAGATCATTTTAAAAGAGAAAACAA
TGTATTTAAAAAATATCTGAATAAACTTTGCCAAGTTGATGATAAATTTCAGGAATCA
CTAAGAATGTTTTGTACATAAATCATTGTTGTAAATAGATTCTTCTGAAGTGCTTCAGT

GTTTGGATTTGATCATTGTCCTTTTGGTGTTACATCATTCATTCAGCAAATCTGAGTTC
ATTTTTTCCCTTAAATGAGCAAACAGTTGGAGTAGCCAAATCTTGAATTAGTGCAGTT
CCAATTACAATTTCGAGGTTCTGGTCTTATCCTTCTCTCTGCCTC

**Figure S2.**

A)

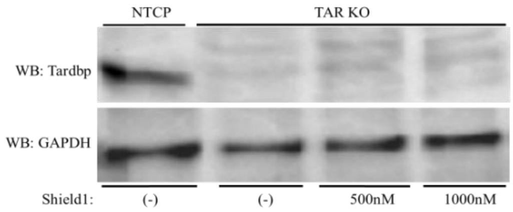
NTCP
TAR KO
WB: Tardbp
WB: GAPDH
Shield1:
(-)
(-)
500nM
1000nM

B)

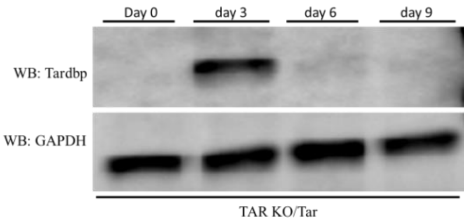
Day 0
day 3
day 6
day 9
WB: Tardbp
WB: GAPDH
TAR KO/Tar

**Figure S3.**

Probe 1 (1804-1849)

S TTCACCAGCACCATGCAACTTTTTCACCTCTGCCTAATCATCTCAT

AS ATGAGATGATTAGGCAGAGGTGAAAAAGTTGCATGGTGCTGGTGAA

Probe 2 (1765-1810)

S GTCTTTGTACTAGGAGGCTGTAGGCATAAATTGGTCTGTTCACCAG

AS CTGGTGAACAGACCAATTTATGCCTACAGCCTCCTAGTACAAAGAC

Probe 3 (1728-1773)

S AAGACTGGGAGGAGTTGGGGGAGGAGATTAGGTTAAAGGTCTTTGT

AS ACAAAGACCTTTAACCTAATCTCCTCCCCCAACTCCTCCCAGTCTT

Probe 4 (1690-1735)

S GACCGACCTTGAGGCATACTTCAAAGACTGTGTGTTTAAAGACTGG

AS CCAGTCTTTAAACACACAGTCTTTGAAGTATGCCTCAAGGTCGGTC

Probe 5(1655-1700)

S CATAAGAGGACTCTTGGACTCTCAGCAATGTCAACGACCGACCTTG

AS CAAGGTCGGTCGTTGACATTGCTGAGAGTCCAAGAGTCCTCTTATG

Probe 6 (1611-1657)

S TGGAGACCACCGTGAACGCCCGCCAGGTCTTGCCCAAGGTCTTACAT

AS ATGTAAGACCTTGGGCAAGACCTGGCGGGCGTTCACGGTGGTCTCCA

**Figure S4.**

A)

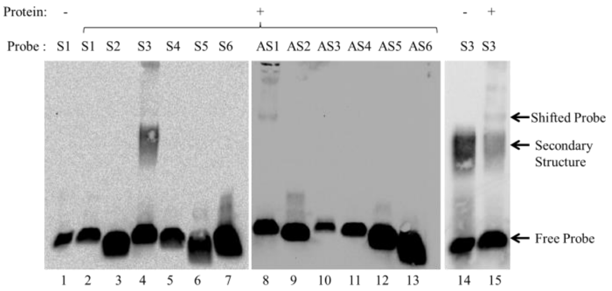


B)

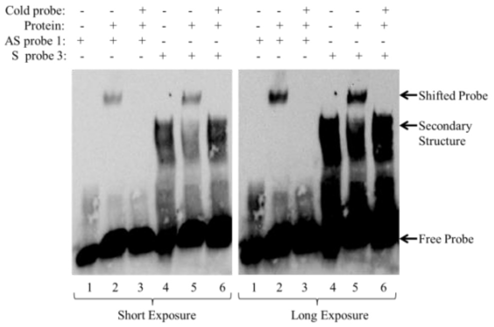


C)

WT(1804-1849 ATGAGATGATTAGGCAGAGGTGAAAAAGTTGCATGGTGCTGGTGAA
s1 (1799-1825) ATGAACATGAGATGATTAGGCAGAGGTG
s2 (1822-1849) GGTGAAAAAGTTGCATGGTGCTGGTGAA
s3 (1818-1841) CAGAGGTGAAAAAGTTGCATGGTG
s4 (1828-1851) AAAGTTGCATGGTGCTGGTGAACA

D)

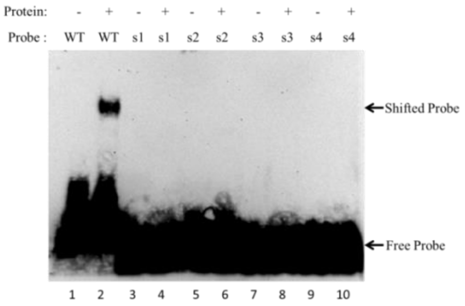

**Figure S5.**

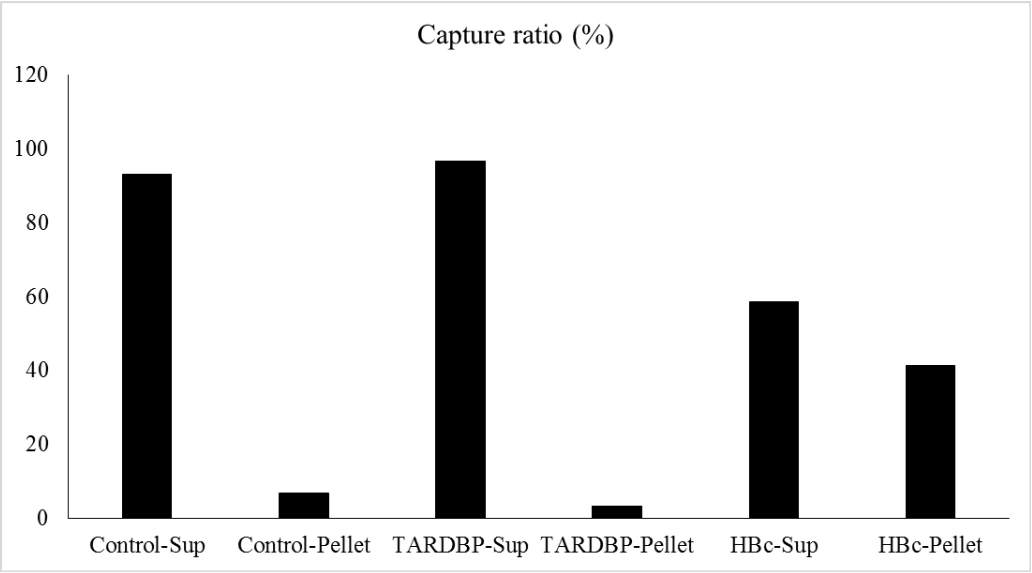
Capture ratio (%)
120
100
80
60
40
20
0
Control-Sup
Control-Pellet
TARDBP-Sup
TARDBP-Pellet
HBc-Sup
HBc-Pellet

**Figure S6.**

A)

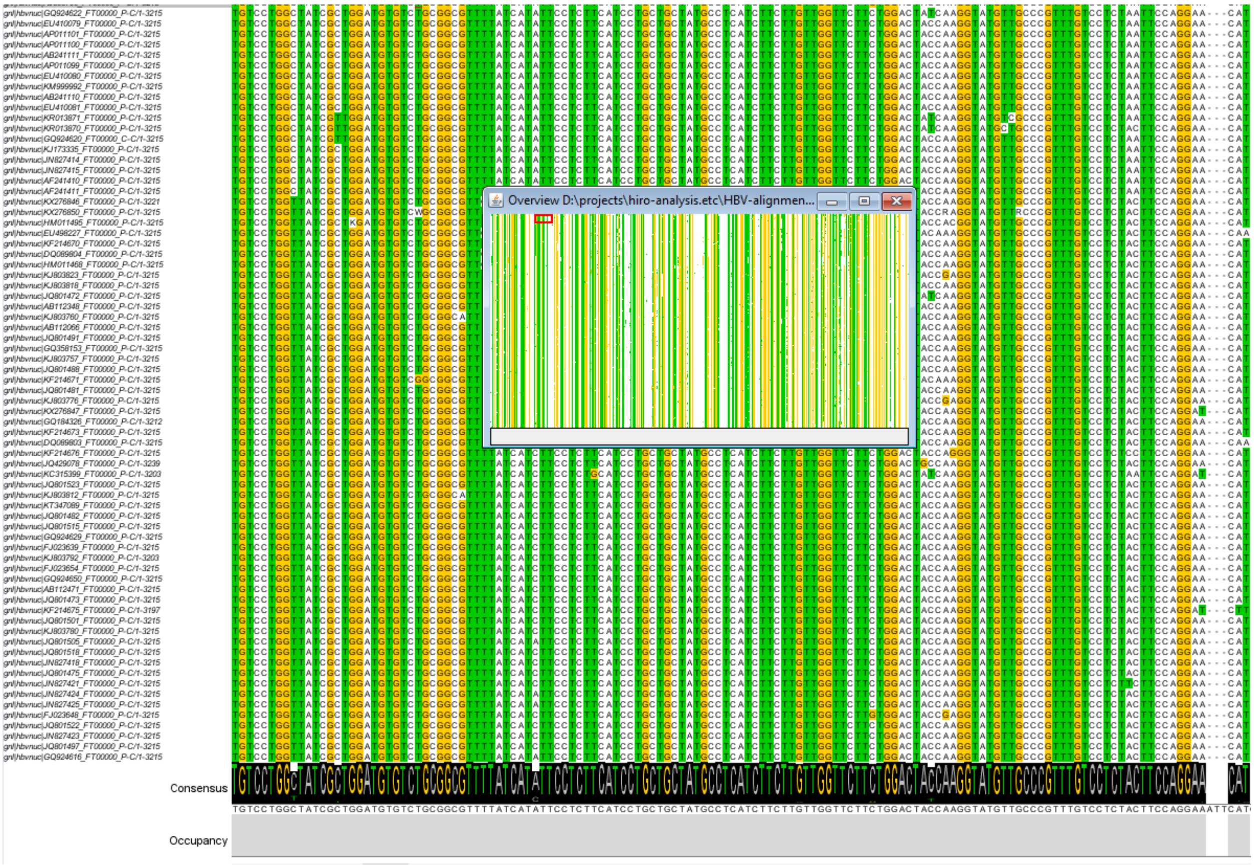


B)

## RBPDB - RNA-binding experiments

Quick tips: Click 'Search' to open search form. Click 'View' to see details. Click column title to sort. Navigation buttons and links to export below table.

| Filter | | | | | |
|---|---|---|---|---|---|
| | PubMed ID | Experiment Type | Reported Sequence or Motif | Gene Name | Reported secondary structure? |
| View | 11470789 | UV cross-linking | UGUGUGUGUGUG | TARDBP | no |
| View | 15826655 | EMSA | GUGUGUGUAAAAAAA | TARDBP | no |
| View | 15826655 | EMSA | GUGUGUGUGUAAAAA | TARDBP | no |
| View | 15826655 | EMSA | AGUGUGUGUGUGU | TARDBP | no |
| View | 16157593 | EMSA | UGUGUGUGUGUGUGUGUGUGUGUG | TARDBP | no |
| View | 16254078 | protein affinity purification | AUACCUUUUUGGGGAGGGGGCAGAGAGC | TARDBP | no |
| View | 19174564 | filter binding assay | UGUGUGUGUGUG | TARDBP | no |
| View | 19174564 | filter binding assay | UGUGUGUGUGUGUGUG | TARDBP | no |
| View | 19429692 | EMSA | UGUGUGUGUGUGA | TARDBP | no |
| View | 20423455 | EMSA | UGAGGUAGUAGGUUGUGUGGUU | TARDBP | no |
| View | 20423455 | EMSA | UGAGGUAGUAGGUUGUGUAGUU | TARDBP | no |

<< < > >> Go to page: 1 ▼ Download displayed records: Page: 1 of 1 Records: 11

C)

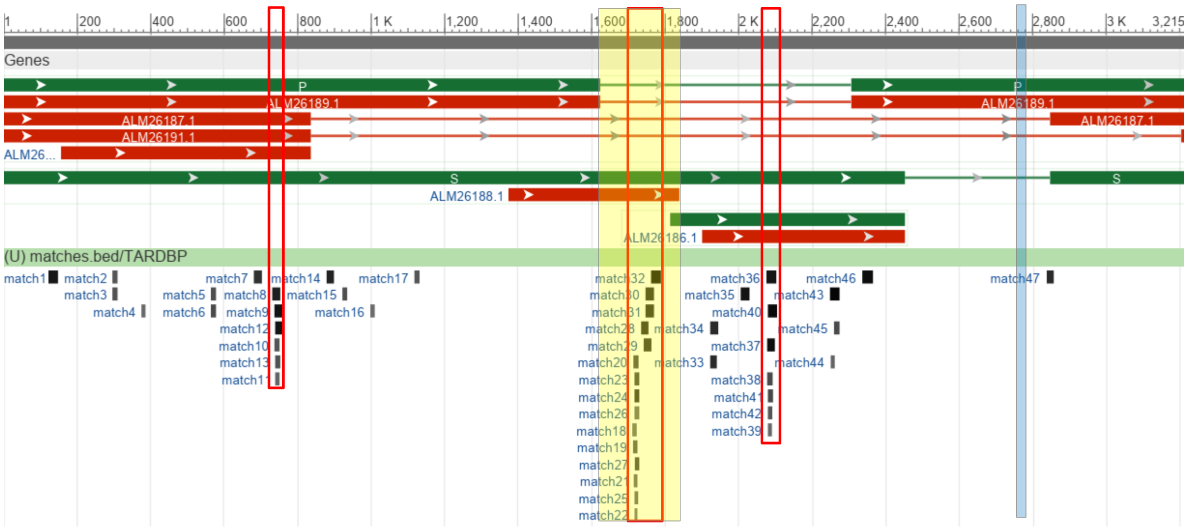

1
200
400
600
800
1 K
1,200
1,400
1,600
1,800
2 K
2,200
2,400
2,600
2,800
3 K
3,215
Genes
P
ALM26189.1
ALM26187.1
ALM26191.1
ALM26...
S
ALM26188.1
ALM26186.1
(U) matches.bed/TARDBP
match1
match2
match3
match4
match5
match6
match7
match8
match9
match10
match11
match12
match13
match14
match15
match16
match17
match18
match19
match20
match21
match22
match23
match24
match25
match26
match27
match28
match29
match30
match31
match32
match33
match34
match35
match36
match37
match38
match39
match40
match41
match42
match43
match44
match45
match46
match47

**Figure S7.**

Full length blots for Fig. 1a

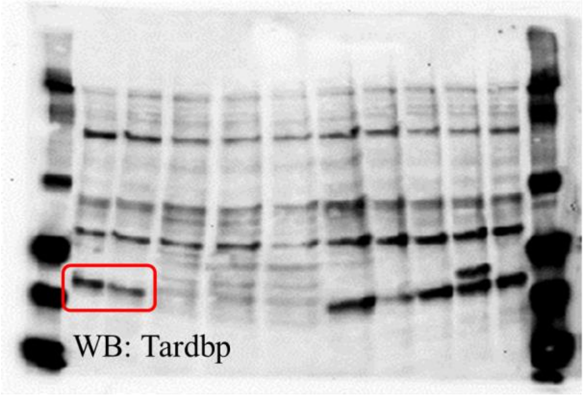


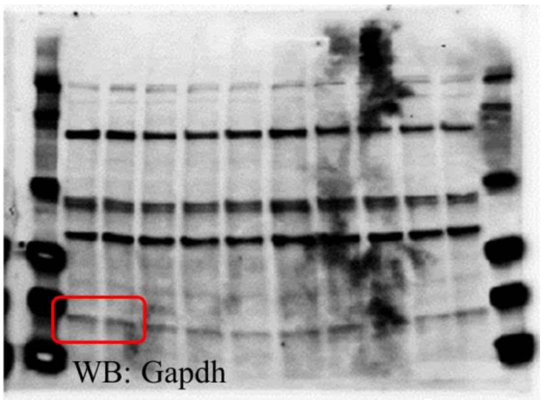


Full length blots for Fig. 1d

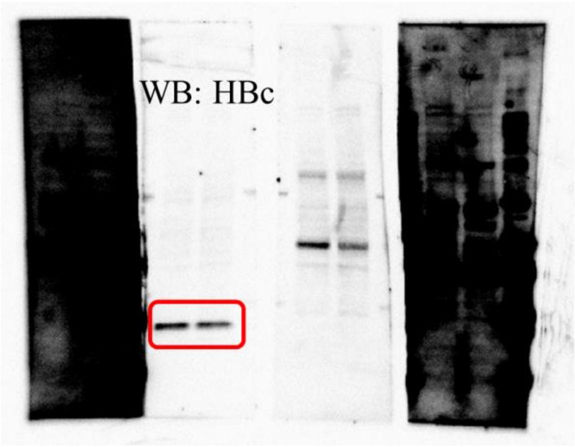


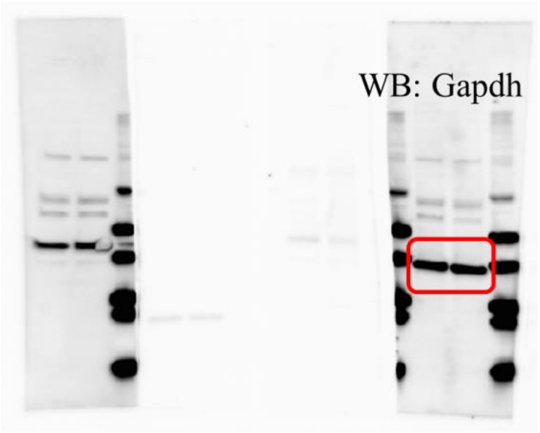


Full length blot for Fig. 1e

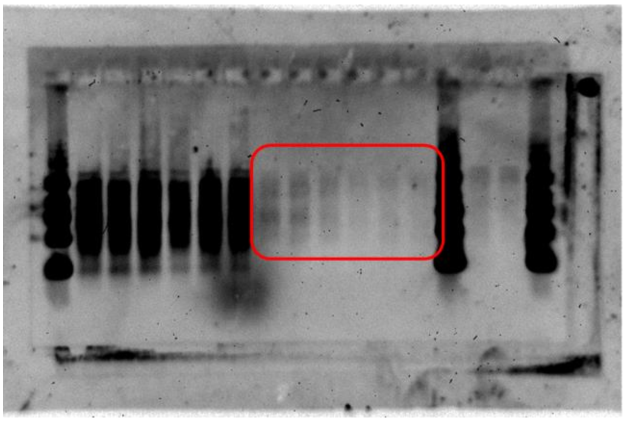

Supplementary data: Full length blots.

The red rectangles indicate the cropped portion of the images used in the figures of the manuscript.

**Figure S8.**

Full length blots for Fig. 2a

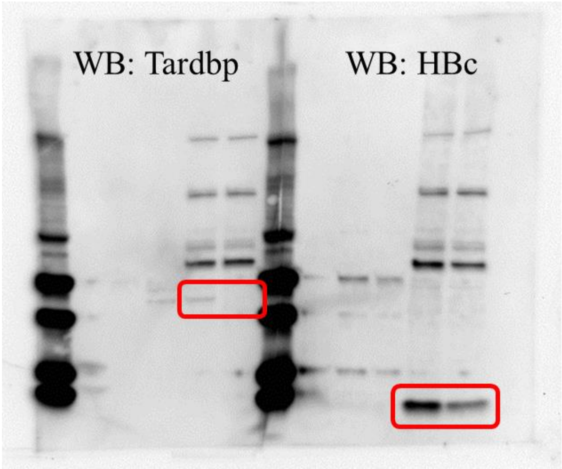


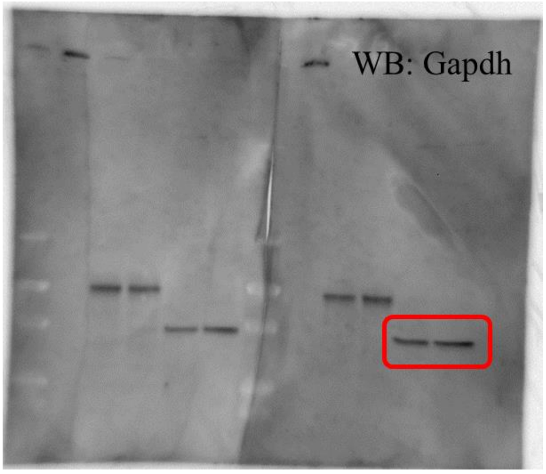


Full length blot for Fig. 2c

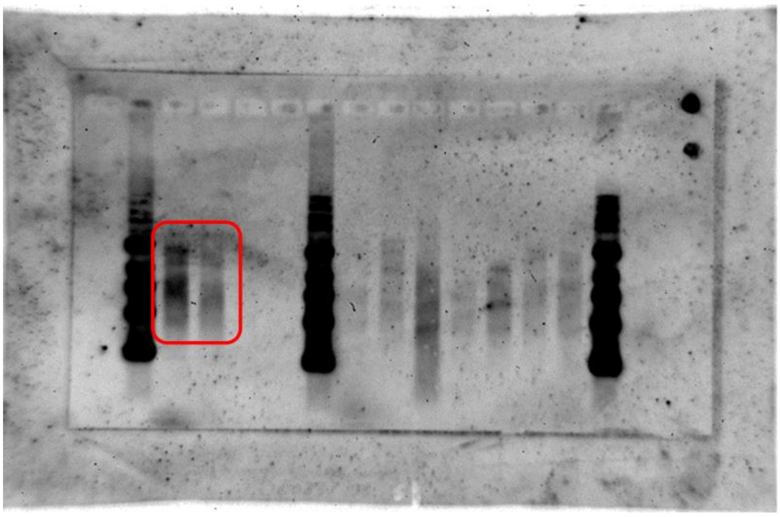

**Figure S9.**

Full length blots for Fig.3c

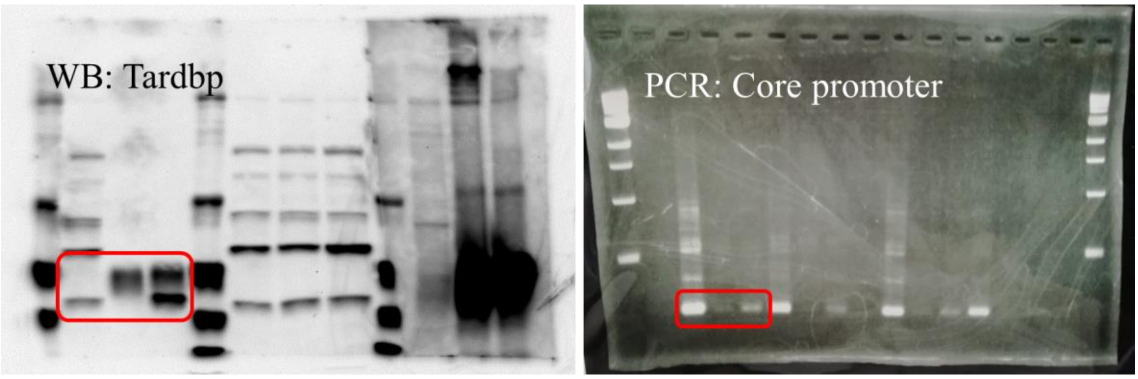


Full length blots for Fig. 3e

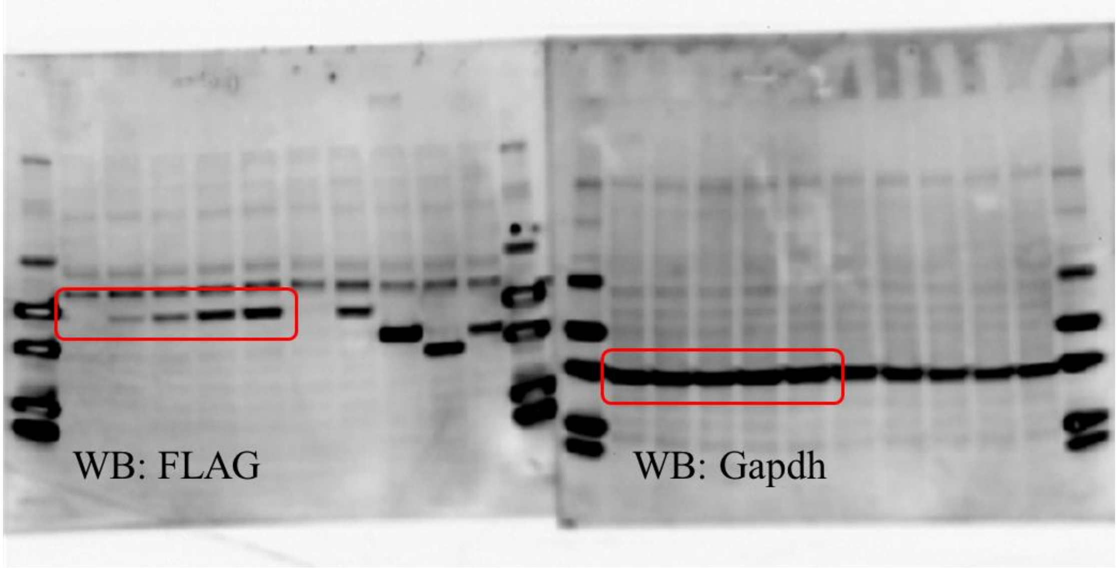

**Figure S10.**

Full length blots for Fig. 4b

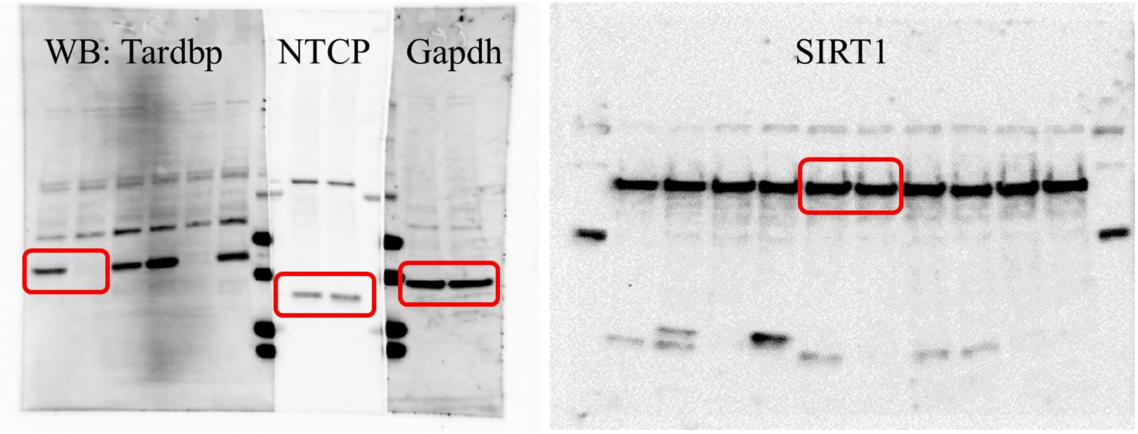


Full length blots for Fig. 4c

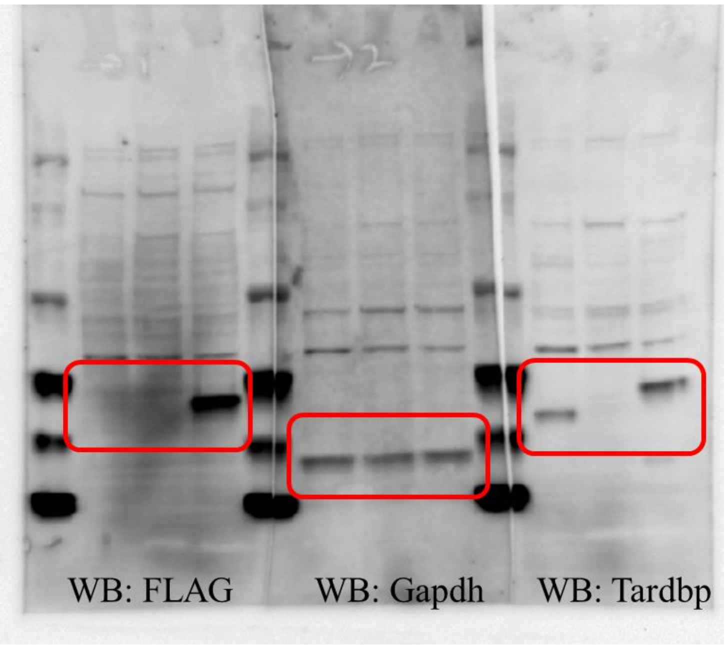

**Figure S11.**

Full length blots for Fig. 5b

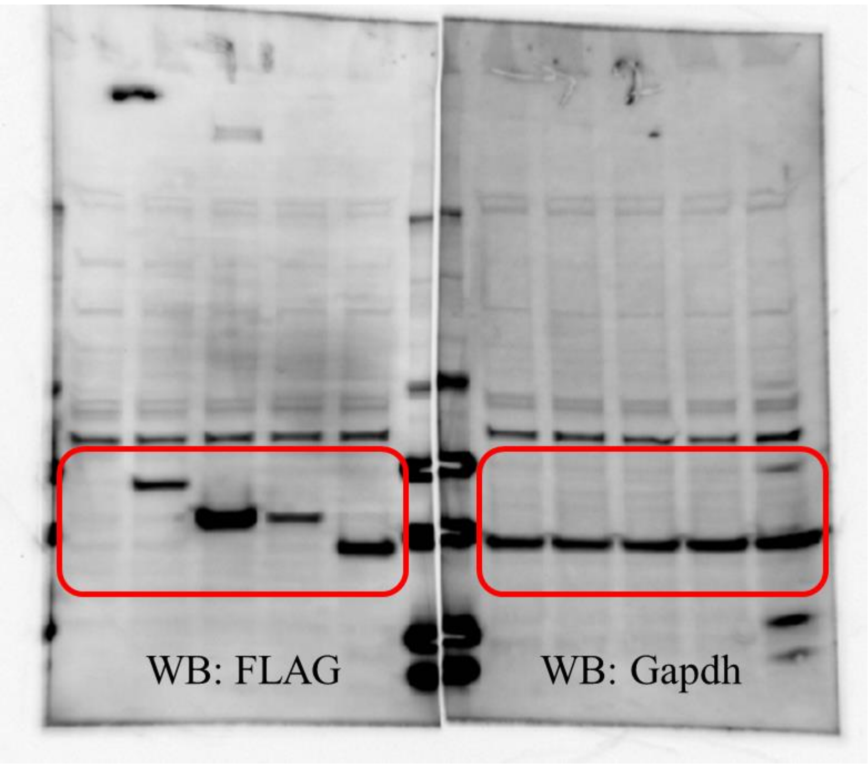

**Figure S12.**

Full length blots for Fig. 6a

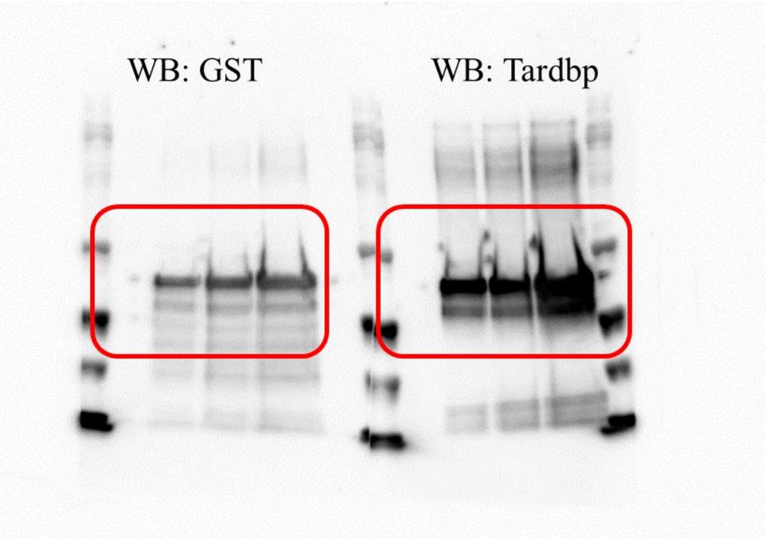


Full length blot for Fig. 6c

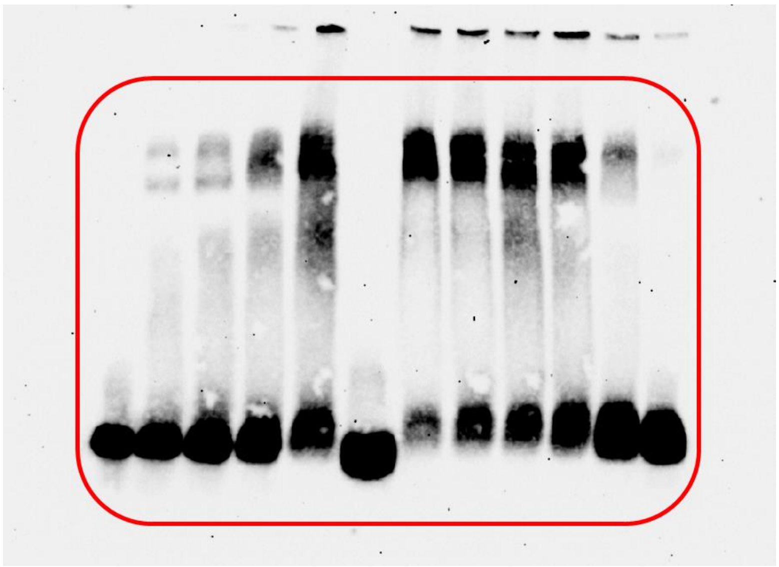

Full length blot Fig. 6d

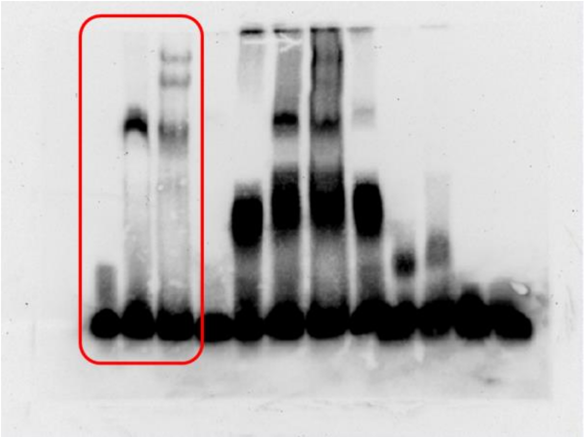

Full length blots Fig. 6e

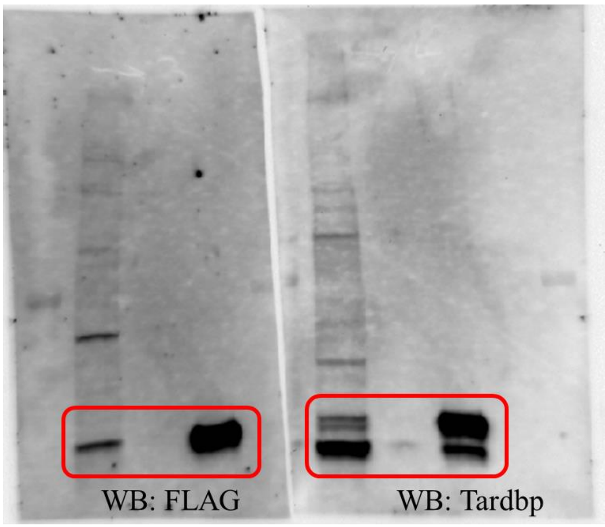


Full length blot Fig. 6f

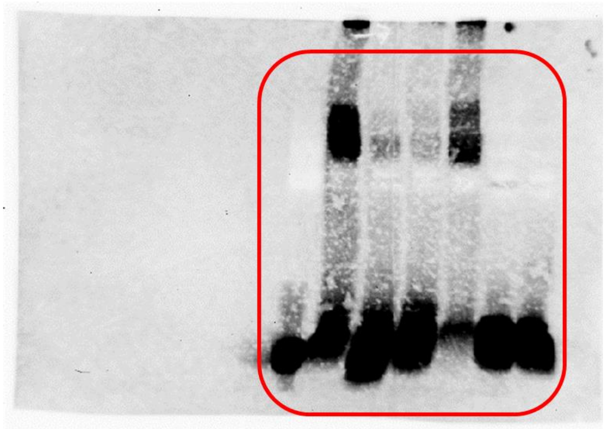

**Figure S13.**

Full length blots for Fig. 7c

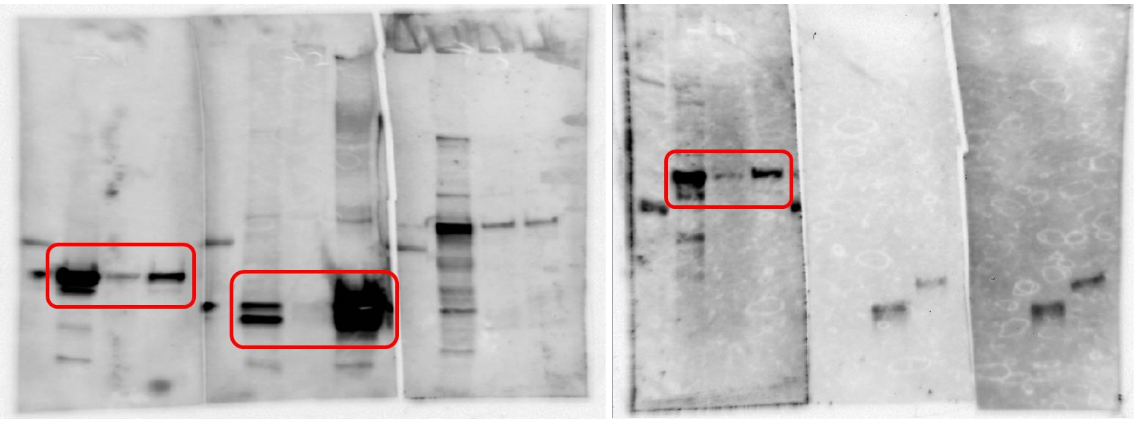

HNRNPK TARDBP SFPQ

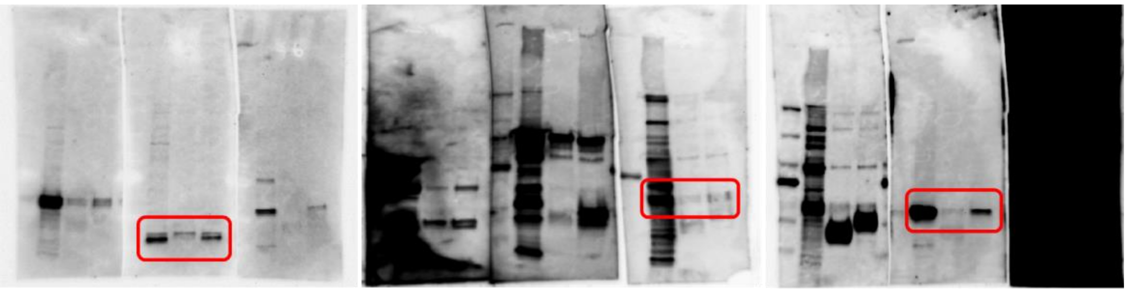

HNRNPC PUF60 PTBP1

**Figure S14.**

Full length blots for Fig. S2a

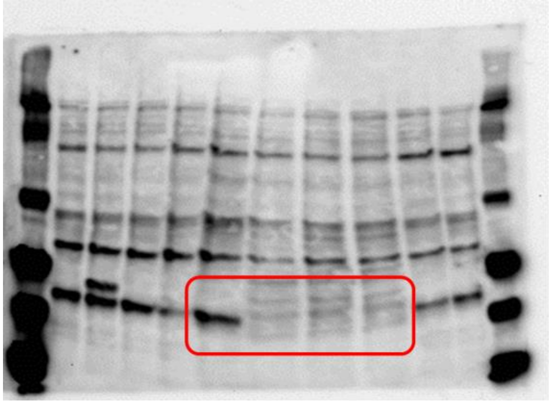

WB: Tardbp

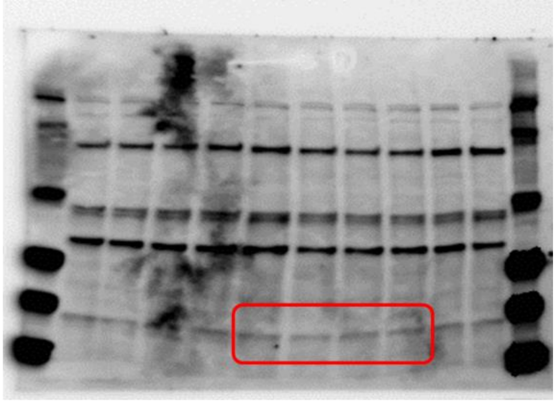

WB: GAPDH

Full length blots for Fig. S2b

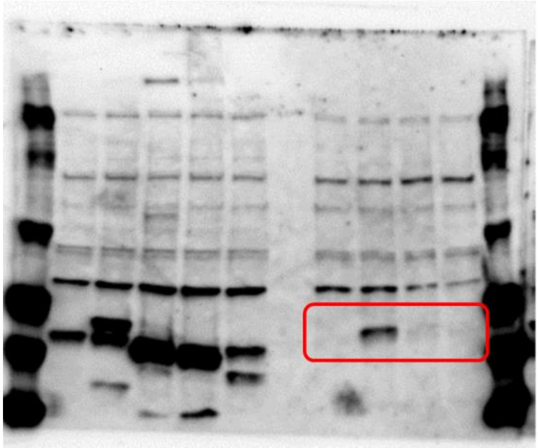

WB: Tardbp

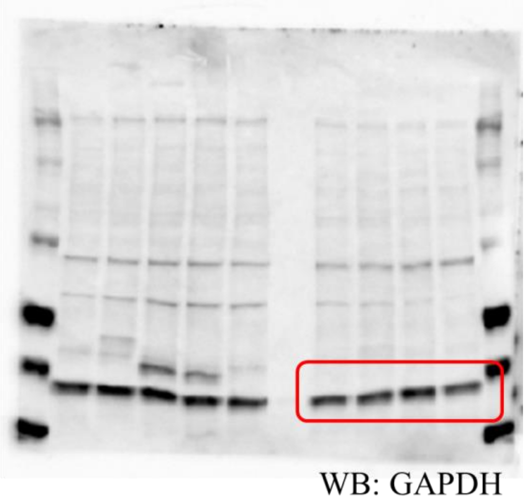

WB: GAPDH
